# The Suppressor of AAC2 Lethality *SAL1* Modulates Sensitivity of Heterologously Expressed *Artemia* ADP/ATP Carrier to Bongkrekate in Yeast

**DOI:** 10.1371/journal.pone.0074187

**Published:** 2013-09-20

**Authors:** Monika Wysocka-Kapcinska, Beata Torocsik, Lilla Turiak, George Tsaprailis, Cynthia L. David, Andrea M. Hunt, Karoly Vekey, Vera Adam-Vizi, Roza Kucharczyk, Christos Chinopoulos

**Affiliations:** 1 Institute of Biochemistry and Biophysics, Polish Academy of Sciences, Warsaw, Poland; 2 Department of Medical Biochemistry, Semmelweis University, Budapest, Hungary; 3 Institute of Organic Chemistry, Research Centre for Natural Sciences, Hungarian Academy of Sciences, Budapest, Hungary; 4 University of Arizona, Center for Toxicology, College of Pharmacy, Tucson, Arizona, United States of America; University of Pittsburgh, United States of America

## Abstract

The ADP/ATP carrier protein (AAC) expressed in *Artemia franciscana* is refractory to bongkrekate. We generated two strains of *Saccharomyces cerevisiae* where *AAC1* and *AAC3* were inactivated and the *AAC2* isoform was replaced with *Artemia AAC* containing a hemagglutinin tag (ArAAC-HA). In one of the strains the suppressor of Δ*AAC2* lethality, *SAL1,* was also inactivated but a plasmid coding for yeast *AAC2* was included, because the *ArAAC*Δ*sal1*Δ strain was lethal. In both strains ArAAC-HA was expressed and correctly localized to the mitochondria. Peptide sequencing of ArAAC expressed in *Artemia* and that expressed in the modified yeasts revealed identical amino acid sequences. The isolated mitochondria from both modified strains developed 85% of the membrane potential attained by mitochondria of control strains, and addition of ADP yielded bongkrekate-sensitive depolarizations implying acquired sensitivity of ArAAC-mediated adenine nucleotide exchange to this poison, independent from *SAL1*. However, growth of ArAAC-expressing yeasts in glycerol-containing media was arrested by bongkrekate only in the presence of *SAL1*. We conclude that the mitochondrial environment of yeasts relying on respiratory growth conferred sensitivity of ArAAC to bongkrekate in a *SAL1*-dependent manner.

## Introduction

Embryos of the brine shrimp *Artemia franciscana* exhibit a type of extremophilia characterized by tolerance of anoxia at room temperature for several years [1] [2] with no evidence of apoptotic or necrotic cell death [3]. Protracted anoxia in mammalian species opens the so-called mitochondrial permeability transition pore (PTP) [4], [5], [6]. This pore is of a sufficient size (cut-off ∼1,5 kDa) to allow passage of solutes and water, causing swelling and ultimately rupture of the organelle. Activation of the PTP by Ca^2+^ overload with the ensuing loss of mitochondrial function results in a severely diminished capacity for energy production and is a final common pathway of cell death [7].

Mitochondria isolated from the embryos of *Artemia franciscana* lack a Ca^2+^-induced PTP [8], despite the capacity for a profound storage for calcium. While the salient components of the PTP have just been unravelled [9], [10] but see [11] the adenine nucleotide carrier (AAC) remains a well-established modulatory component of this pore [12], [13]. All known ligands of the AAC modulate the probability for PTP opening [12]. Relevant to this, *Artemia franciscana* is the only species known in which adenine nucleotide exchange operated by AAC is refractory to the naturally occurring inhibitor, bongkrekic acid (BKA) [14]. In species where Ca^2+^-induced mitochondrial PTP can be demonstrated, BKA inhibits adenine nucleotide exchange mediated by the AAC isoforms they express, and decreases the probability of PTP opening [15], [16], [17], [12]. Recently however, we have reported that mitochondria obtained from brown shrimp (*Crangon crangon*) and common prawn (*Palaemon serratus*) exhibit BKA-sensitive mitochondrial adenine nucleotide transport while lacking a Ca^2+^-induced permeability transition [18].

A plausible hypothesis is that AAC interacts with a yet to be identified protein that initiates PTP opening, and that this protein interaction is affected by BKA. An obvious impediment in the elucidation of the role of AAC in PTP and its inhibition by BKA is the lack of knowledge regarding the binding site of BKA on AAC [12], [19]. In the AAC protein sequence found in *Artemia*, the region between residues 198 and 225 exhibits a low degree of similarity with AAC sequences from other species [14]. We therefore postulated that the BKA binding site may reside somewhere within this *Artemia* AAC region. To verify this hypothesis, a base-by-base alteration is warranted to pinpoint residues that are critical for the binding of BKA. Insufficient information regarding the genetic background of *Artemia franciscana* prompted us to express *Artemia AAC (ArAAC)* in a heterologous environment amenable to genetic manipulations. Yeast is an excellent platform for such experiments. However, as it will become evident from the ‘Results’ section below, adenine nucleotide exchange mediated by heterologously expressed *ArAAC* expressed in *Saccharomyces cerevisiae* was sensitive to BKA. In addition, due to substitution of endogenous yeast AAC2 carriers which are also important for cell respiration and viability with ArAAC, it was necessary to manipulate the presence of the suppressor of *AAC2* lethality, *SAL1*. *SAL1* is required for growth of yeasts when *AAC2* is absent or inhibited by BKA [20]. Contrary to our expectation, the viability of yeasts expressing *ArAAC* under non-fermenting conditions was arrested by BKA only when *SAL1* was coexpressed while in the absence of Sal1p, growth of yeasts expressing *ArAAC* was BKA resistant. In direct contrast, under fermenting conditions the viability of yeasts expressing *ArAAC* was arrested by BKA only when *SAL1* was absent, indicating the lethality of *ArAAC sal1Δ*.

## Results

### Expression of ArAAC in Yeast Cells

**Figure 1:**
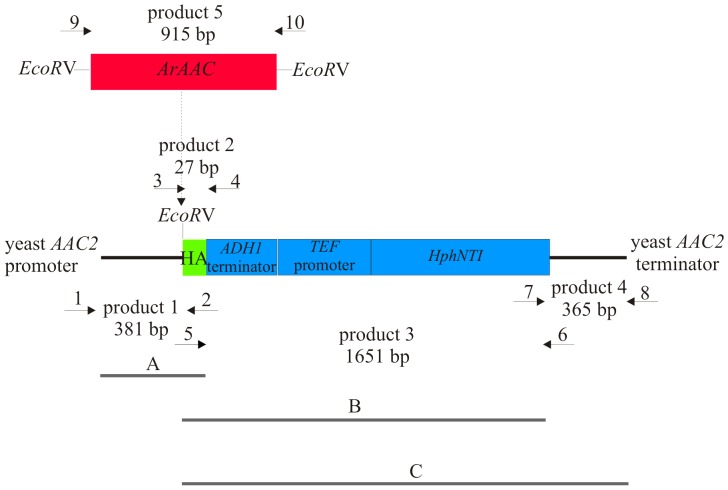
*ArAAC* integration cassette including coding sequence of *ArACC* gene fused to HA tag and hygromycin cassette (*HphNTI*) flanked by promoter and terminator sequences of yeast *AAC2* gene. The order of PCR reactions is described in ‘Materials and Methods’ section. The positions of the primers used for PCR reactions (marked by numbers corresponding to the numbered primers listed in Table 2) and the length of DNA fragments are also indicated.

We integrated the *ArAAC* gene into the locus of the main yeast AAC gene – *AAC2* (Figure 1). As the double *aac2 sal1* deletion strain is lethal and a functional Sal1p is required for growth of yeast in the presence of BKA which blocks the operation of AAC2 protein [20], the *ArAAC* was expressed in *SAL1* and *sal1::NatMX4* deletion background. We first amplified *ArAAC* using cDNA from reverse transcribed total *Artemia franciscana* RNA as template and cloned it into a TOPO-TA Cloning Vector (TOPO TA Cloning*®* Kits for Sequencing, Invitrogen). The *ArAAC* integration cassette containing the *ArAAC*-*HA* tagged gene and the hygromycin resistance gene *HphNTI* was constructed as described in ‘Materials and Methods’. The cassette DNA was transformed into a strain bearing deletions of two other AAC genes present in yeast, *AAC1* and *AAC3* (RKY67-1C), resulting in MWY79/15 and MWY79/17 clones bearing *ArAAC*-*HA* gene in the locus of *AAC2* (Table 1). We then deleted the *SAL1* gene in control and *ArAAC* expressing strains MR6, RKY67-1C and MWY79/15 by transforming them with *sal1::NatMX4* cassette (see under ‘Materials and Methods’), resulting in strains: MWY85/9, MWY84/3, MWY83/1 and 5 (Table 1). ArAAC could not rescue yeasts in *sal1* background, thus the deletion of *SAL1* gene in *ArAAC* background was done in the presence of a wild type copy of yeast *AAC2* on a Yep352 plasmid (MWY83 strains). A most appropriate isogenic control for our ArAAC-expressing constructs would be to reintroduce the AAC2 gene in the same manner, with the HA tag and the resistance gene cassette in the same positions, as performed in [21] and [22]. However, proteomic analysis of yeast mitochondria expressing ArAAC verified that the sequence is identical to that expressed in *Artemia franciscana* (see below). Moreover, mitochondria from the MWY79/15 strain, where no endogenous AAC was present, achieved 85% of total attainable membrane potential, and ADP-ATP exchange was readily demonstrated (see below). These results afford a reasonable degree of assurance that the genetic manipulations in our strains did not confer confounding variables to the results.

**Table 1:** Strains used in this work.

Strain	Nuclear genotype	Source
MR6	*MATa ade2-1 his3-11,15 trp1-1 leu2-3,112 ura3-1 arg8::HIS3*	[63]
RKY67-1C	*MATa ade2-1 his3-11,15 trp1-1 leu2-3,112 ura3-1 arg8::HIS3 aac1ΔKanMX4 aac3ΔKanMX4 AAC2*	This study
RKY67-1D	*MATa ade2-1 his3-11,15 trp1-1 leu2-3,112 ura3-1 arg8::HIS3 aac1ΔKanMX4 aac3ΔKanMX4 AAC2*	This study
MWY80	*MATa ade2-1 his3-11,15 trp1-1 leu2-3,112 ura3-1 arg8::HIS3 aac1ΔKanMX4 aac3ΔKanMX4 aac2ΔHphNTI*	This study
MWY85/9	*MATa ade2-1 his3-11,15 trp1-1 leu2-3,112 ura3-1 arg8::HIS3 sal1Δ::NatMX4*	This study
MWY79/15	*MATa ade2-1 his3-11,15 trp1-1 leu2-3,112 ura3-1 arg8::HIS3 aac1ΔKanMX4 aac3ΔKanMX4 aac2Δ::ArAAC-HA-HphNTI*	This study
MWY79/17	*MATa ade2-1 his3-11,15 trp1-1 leu2-3,112 ura3-1 arg8::HIS3 aac1ΔKanMX4 aac3ΔKanMX4 aac2Δ::ArAAC-HA-HphNTI*	This study
MWY84/3	*MATa ade2-1 his3-11,15 trp1-1 leu2-3,112 ura3-1 arg8::HIS3 aac1ΔKanMX4 aac3ΔKanMX4 sal1Δ::NatMX4*	This study
MWY83/1	*MATa ade2-1 his3-11,15 trp1-1 leu2-3,112 ura3-1 arg8::HIS3 aac1ΔKanMX4 aac3ΔKanMX4 aac2Δ::ArAAC-HA-HphNTI sal1Δ::NatMX4 [AAC2/pYep352]*	This study
MWY83/5	*MATa ade2-1 his3-11,15 trp1-1 leu2-3,112 ura3-1 arg8::HIS3 aac1ΔKanMX4 aac3ΔKanMX4 aac2Δ::ArAAC-HA-HphNTI sal1Δ::NatMX4 [AAC2/pYep352]*	This study

**Figure 2:**
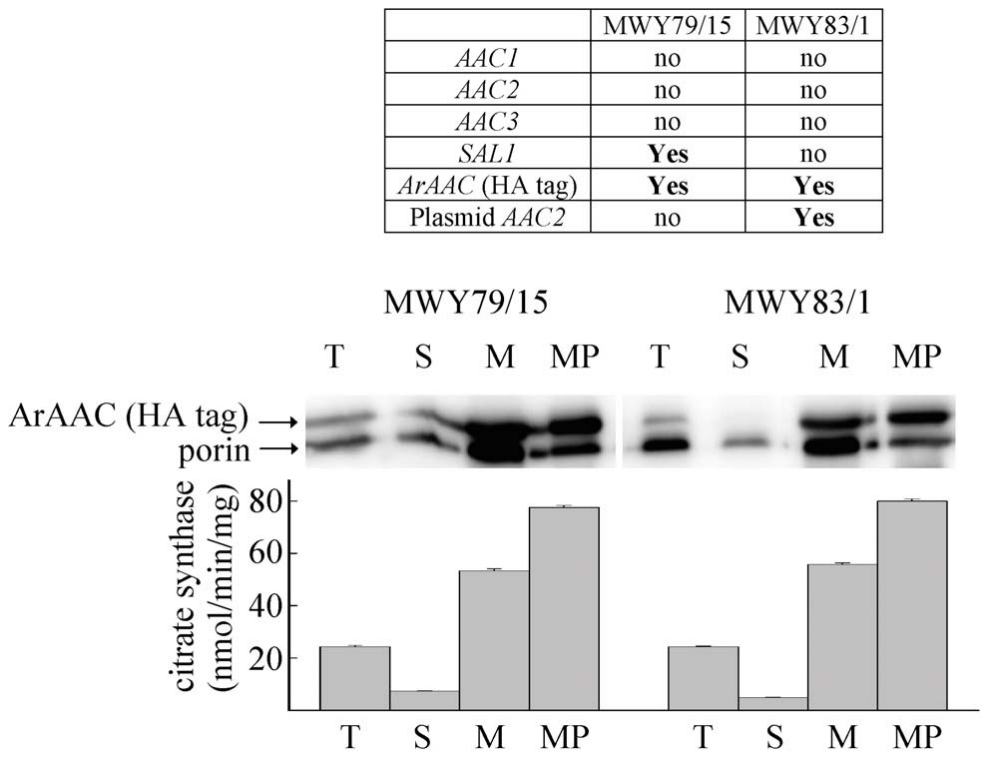
The ArAac protein is expressed and targeted to yeast mitochondria. Total extract (T), mitochondrial (M), post-mitochondrial supernatant (S) and mitoplast (MP) fractions were prepared from strains expressing heterologous *Artemia* Aac proteins as indicated in the table on the top: ‘no’ signifies absence of the gene indicated in the left-most column; ‘**Yes**’ signifies the presence of the gene indicated in the left-most column. Samples of 25 µg of each fraction were separated on 10% polyacrylamide gel electrophoresis. Blots were probed for ArAAC (HA tag) and porin in the same solutions. The Western blot results shown are typical from three independent experiments. In the lower bar graph, citrate synthase activities of the same fractions are depicted.

The proper localization of ArAAC protein in mitochondria was verified by yeast cell extract fractionation, Western blotting and estimations of citrate synthase activity in the various fractions (Figure 2). Although there is an extramitochondrial citrate synthase in yeasts [23], the overall activity is mainly due to that residing in the mitochondrial matrix. During preparation of the mitochondria, fractions from the two strains expressing ArAAC with (MWY79/15) and without (MWY83/1) Sal1p were probed for the presence of ArAAC (through its HA tag) and porin by Western blotting and citrate synthase activity. Furthermore, mitoplasts were also generated from mitochondria and these were also probed for the presence of ArAAC (through its HA tag) and porin by Western blotting and citrate synthase activity. The fractions were: T (total yeast homogenates), S (supernatant), M (mitochondria), and MP (mitoplasts). The probing for ArAAC (through its HA tag) and porin was performed in the same blot, thus the bands appear very close to each other due to the similar molecular weights of ArAAC and porin. As shown in Figure 2, the band representing ArAAC in the Western blot and the corresponding citrate synthase activity in the MP fraction is greater than the M fraction, but at the same time the band corresponding to porin in the M fraction is greater than the signal from the MP fraction. This is consistent with the notion that porin resides in the outer mitochondrial membrane, which is partially removed during the preparation of the MP fraction, while ArAAC is in the inner membrane/matrix side. Thus, ArAAC protein was expressed and targeted into the yeast inner membrane/matrix side. The experiments shown below (especially for the strain MWY79/15 where no endogenous AAC is present) argue that there was indeed ADP-ATP exchange, and validate the claim that ArAAC was correctly targeted to the inner mitochondrial membrane.

### Effect of BKA on Viability of the Modified Strains

**Figure 3:**
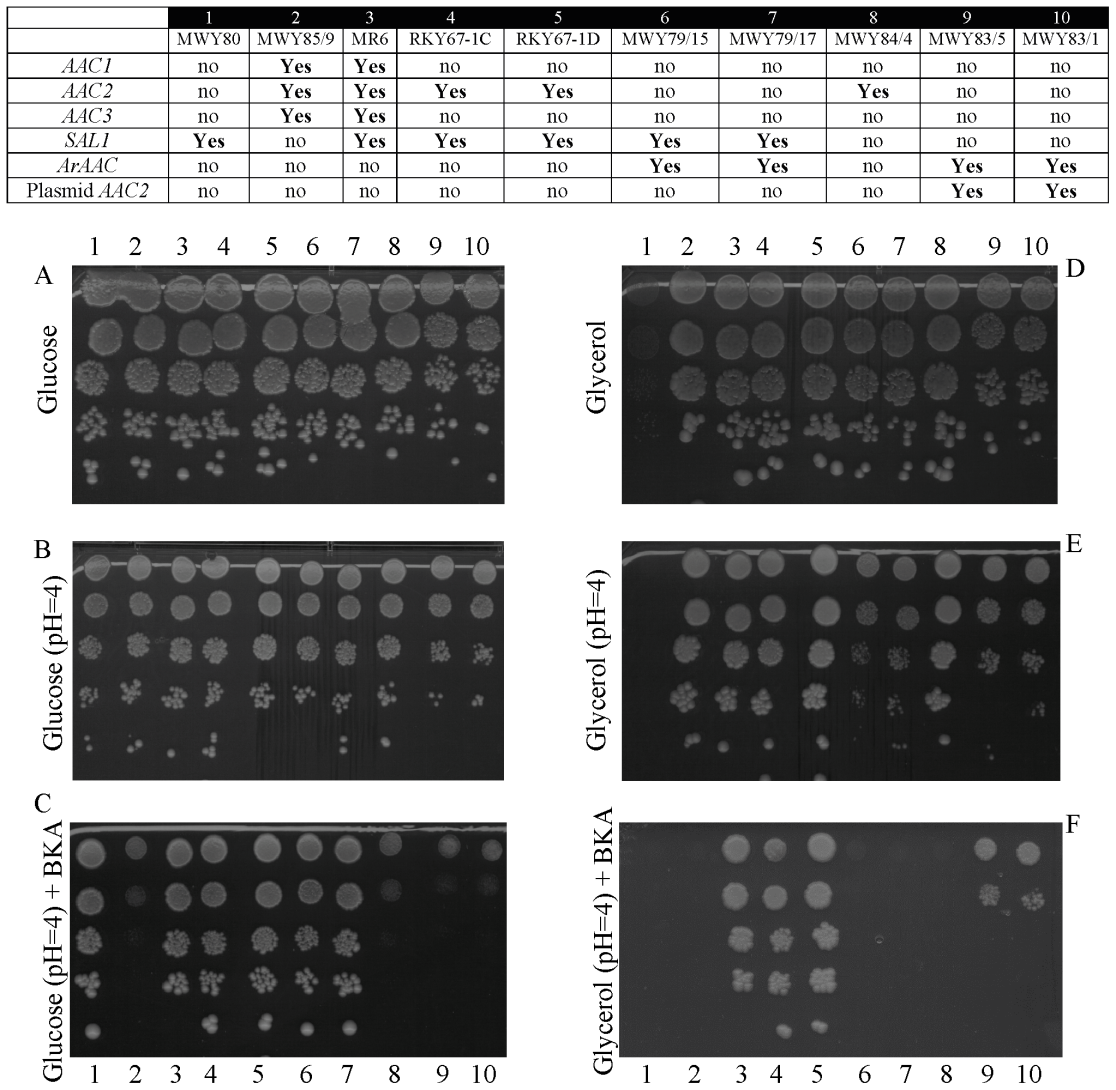
Viability of modified and control yeast strains in response to BKA (1 M for the glucose-containing plates, and 20 nM for the glycerol-containing plates). Yeast strains were grown on media indicated in the panels in the absence or presence of BKA at 28°C. Each lane contains spots with initially 10^6^, 10^5^, 10^4^, 10^3^ and 10^2^ cells (top to bottom). The genotypes of the strains are given in the table shown on the top of the figure: ‘no’ signifies absence of the gene indicated in the left-most column; ‘Yes’ signifies the presence of the gene indicated in the left-most column. 1 (MWY80) - *aac1Δ aac2Δ aac3Δ*, 2 (MWY85/9) - *sal1Δ*, 3 (MR6) – *WT*, 4 (RKY67-1C), 5 (RKY67-1D) – two independent clones *aac1Δ aac3Δ AAC2*, 6 (MWY79/15), 7 (MWY79/17) - two independent clones *aac1Δ aac3Δ ArAAC*, 8 (MWY84/4) - *aac1Δ aac3Δ AAC2 sal1Δ*, 9 (MWY83/5), 10 (MWY83/1) - two independent clones *aac1Δ aac3Δ ArAAC sal1Δ [AAC2].* Plates were scanned after 4 (glucose) or 6 (glycerol) days of incubation.

*AAC2* is responsible not only for yeast growth in non-fermentable sources, termed the respiratory function (R function), but also possesses an essential role in maintaining cell mitotic viability on fermentable carbon sources, termed the V function [24]. Mindful that a functional Sal1p is required for growth of yeasts when Aac2p is absent or inhibited by BKA [20], we addressed the function of ArAAC and its response to BKA by performing growth analysis on fermentable (glucose) and non-fermentable (glycerol) carbon sources in the presence or absence of BKA. BKA enters intact yeast cells at pH 4, thus the growth was tested at this pH [20]. Two different clones harboring the same genetic manipulations were used in order to gain additional reinforcement that the observed results were genuine, and not confounded by the genetic background: therefore, experiments shown in lanes 4/5, 6/7, and 9/10 harbor the same genetic manipulations but were performed in different yeast strains (top table within Figure 3). As shown in Figure 3, yeast cells expressing *AAC2* required a functional *SAL1* gene for surviving on glucose-containing media (V function) in the presence of BKA (compare lanes 4 and 5 with lane 8 of panels B and C). The strains expressing *ArAAC* in lieu of *AAC2* also required a functional *SAL1* gene for surviving on glucose-containing media in the presence of BKA, indicating that *ArAAC* together with the *sal1::NatMX4* deletion is lethal (compare lanes 6 and 7 with lanes 9 and 10 in panels B and C). We confirmed this phenotype by testing the ability of MWY83 strains to grow on 5-Fluoroorotic Acid (5-FOA) plates. 5-FOA is converted to a toxic form (i.e., 5-flurouracil) in strains expressing the functional *URA3* gene coding for orotine-5-monophosphate decarboxylase that is involved in the synthesis of uracil. Yeast strains that are phenotypically Ura+ become Ura- and 5-FOA(R) (resistant) after selection. We considered that the *URA3* plasmid-born copy of *AAC2* gene in the presence of 5-FOA in the medium could be lost. However, the MWY83 strains were not able to grow on this medium, confirming the *ArAAC sal1::NatMX4* lethality (data not shown). Regarding yeast viability in glycerol media where cells rely on oxidative phosphorylation (R function) in relation to *SAL1*, *ArAAC* complemented the R function of yeast *AAC2*. This can be seen in panels D and E when comparing the triple deletion lane 1 (where no *AAC* isoform was expressed and despite the presence of Sal1p no yeast growth was observed), and lanes 4 and 5 (where both *AAC2* and *SAL1* were expressed), to lanes 6 and 7 (where *ArAAC* was expressed in lieu of *AAC2*). It is noted though, that yeast expressing *ArAAC* exhibited slower respiratory growth than those expressing *AAC2* (compare the growth of strains in lanes 1, 4 and 5 with lanes 6 and 7 on glycerol medium of panels D and E in Figure 3). In the presence of Sal1p, *ArAAC* expressed instead of *AAC2* conferred sensitivity to BKA; this is reflected in lanes 6 and 7 of panel F, whereas in the absence of Sal1p, it conferred resistance to BKA, inferred from the growth of cells shown in lanes 9 and 10 of panel F where *ArAAC* and *AAC2* (from the plasmid) were expressed. Because *sal1* yeasts expressing ArAAC can grow in the presence of BKA in glycerol but not glucose media, we concluded that ArAAC could not complement the viability (V) function of yeast Aac2p.

### Adenine Nucleotide Exchange Mediated by Yeast-expressed ArAAC is Sensitive to BKA

**Figure 4:**
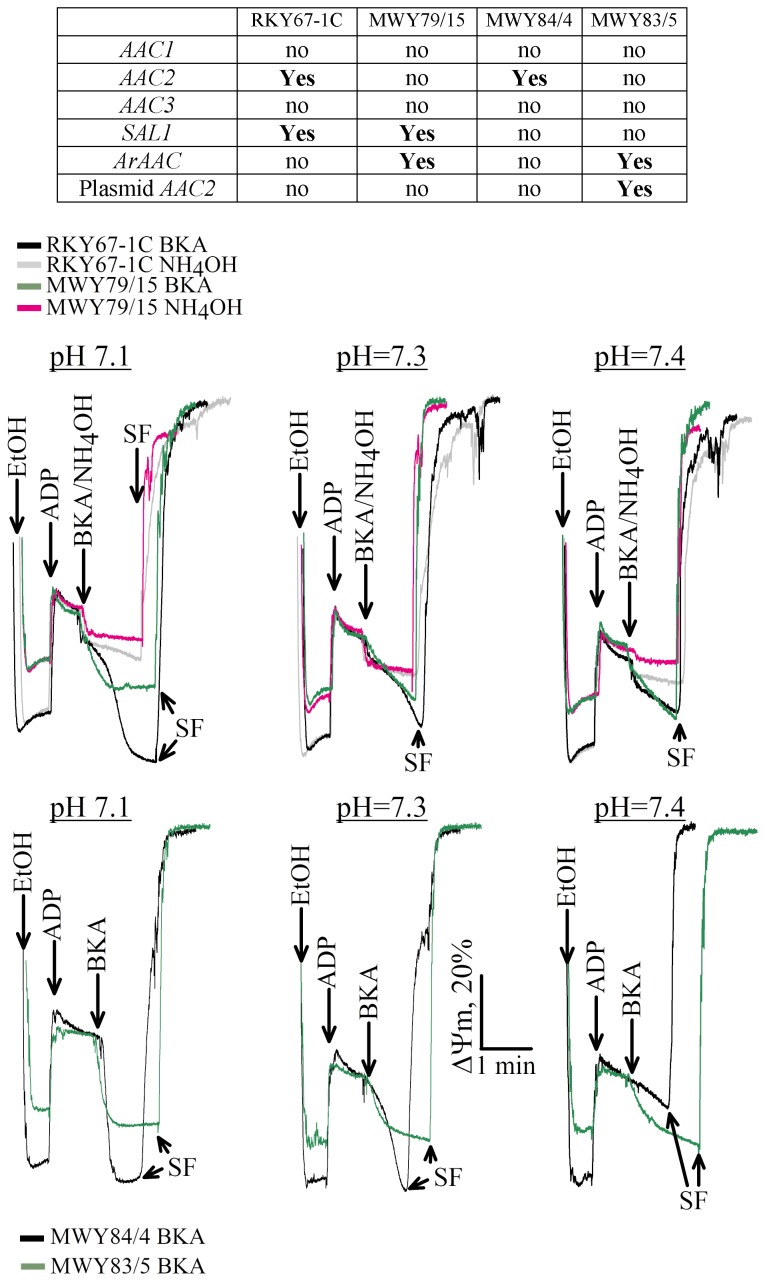
The ArAac protein expressed in yeasts is sensitive to BKA. In the table on top the yeast strains and their expression of adenine nucleotide carriers and *SAL1* is indicated; ‘no’ signifies absence of the gene indicated in the left-most column; ‘**Yes**’ signifies the presence of the gene indicated in the left-most column. In the panels, reconstructed time courses of rhodamine 123 fluorescence (expressed as % of m) as functions of time are shown. In the top three panels mitochondria from strains RKY67-1C (black and grey traces) and MWY79/15 (green and magenta traces) express *SAL1*. In the bottom three panels, mitochondria from strains MWY84/4 (black traces) and MWY83/5 (green traces) do not express *SAL1*. The pH of the media is indicated on the top of each panel. EtOH signifies ethanol.

To test the effect of BKA on ADP/ATP exchange by *ArAAC* expressed in yeasts, we relied on the electrogenic property of the carrier during adenine nucleotide exchange. Mitochondria isolated from the yeast strains detailed below were energized by ethanol, and the effect of BKA during ADP-induced depolarization was evaluated by recording membrane potential (m) with rhodamine 123. To calibrate the rhodamine fluorescence signal, at the end of all experiments the uncoupler SF 6847 (SF, 1 M) was added to induce complete depolarization. The yeast strains used for these experiments are shown in the table on the top of Figure 4. As shown, they either express *AAC2* plus *SAL1* (RKY67-1C), or *ArAAC* plus *SAL1* (MWY79/15) or only *AAC2* (MWY84/4) or *ArAAC* plus *AAC2* from a plasmid (MWY83/5). Because BKA needs to be protonated in order to exert its action on the carrier [25], [26], it becomes less effective at increasing pH. We therefore tested the effect of BKA versus its vehicle (NH_4_OH) in the pH range of 7.1–7.4. Isolated mitochondria (1 mg) were added in a buffer the composition detailed in ‘Materials and Methods’ with a pH indicated in the panels of Figure 4, in the presence of rhodamine 123 (0.5 M). Ethanol (20 l of 96%) was immediately added in order to energize mitochondria, and this was reflected by a decrease in rhodamine fluorescence. In *ArAAC*-expressing cells (green or magenta traces) there was a 15% decrease in mitochondrial polarization as compared to other strains (black or grey traces). After 50 sec, 2 mM ADP was added as indicated in Figure 4, resulting in an immediate depolarization due to the electrogenic exchange of ATP^4−^ for ADP^3−^ through the adenine nucleotide carrier. As soon as a new plateau of m was established, BKA (1 M) or NH_4_OH (1 mM) was added as indicated in Figure 4. In accordance with the results reported previously [14], NH_4_OH exerted a minor depolarizing effect. It is also apparent from all panels that mitochondria obtained from all strains exhibited BKA-induced repolarizations. As expected, the extent of BKA-induced repolarization was dependent on pH, but even at a condition at pH 7.4 where BKA had only a minimal effect on Aac2p-mediated adenine nucleotide exchange (black trace bottom right), it potently abolished ADP-induced depolarization in *ArAAC*-expressing mitochondria (same panel, green trace). Since BKA induced repolarization in mitochondria obtained from all strains, we concluded that the *ArAAC* expressed in yeasts became sensitive to BKA. Moreover, in contrast to the viability results shown above, ArAAC was sensitive to BKA in a manner independent of *SAL1*.

### Mass-spectrometry of Mitochondria Isolated from Artemia Franciscana Embryos and from Yeasts Expressing ArAAC

**Figure 5:**
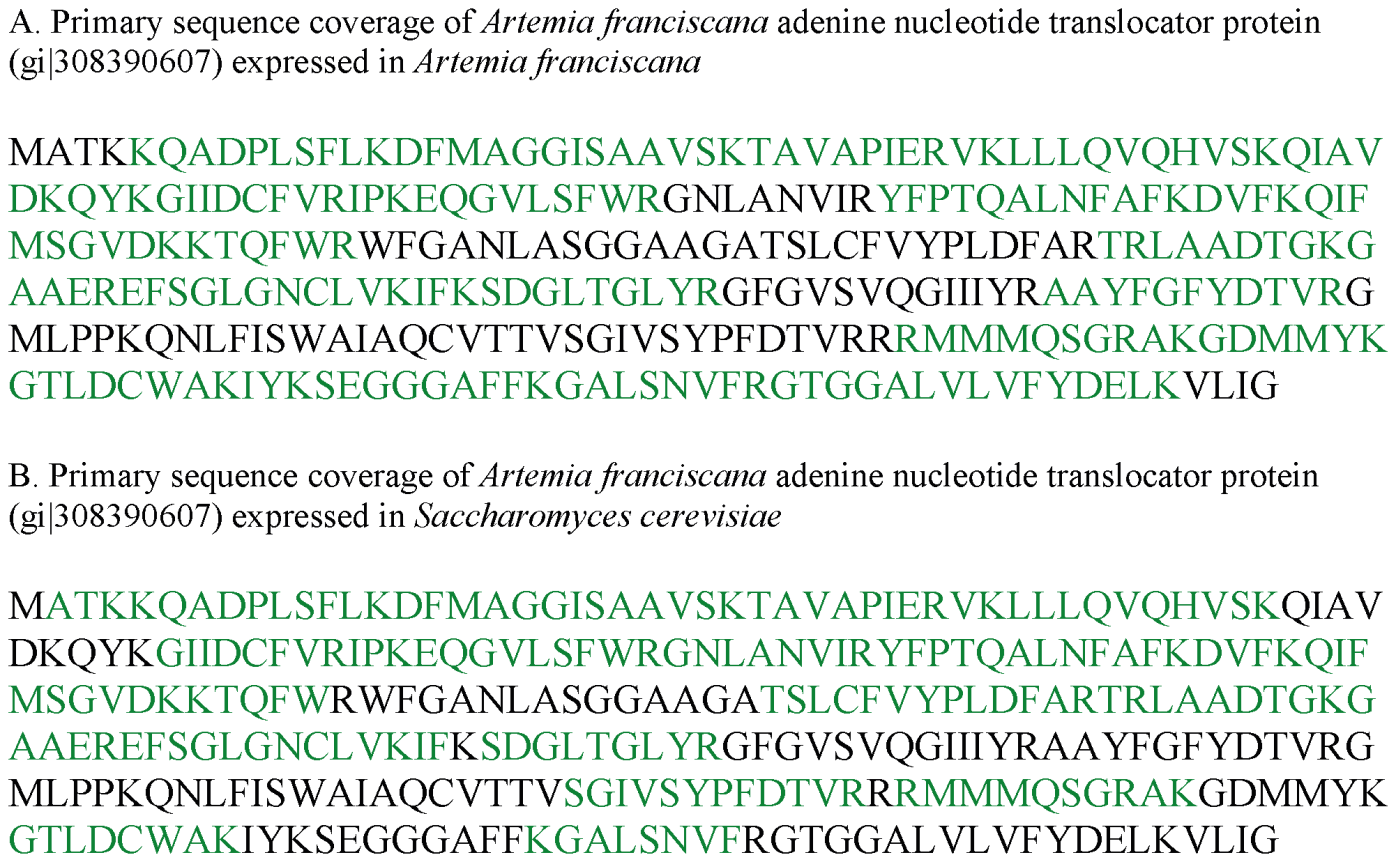
Primary sequence coverage of *ArAAC* expressed in yeast and *ArAAC* expressed in *Artemia franciscana* as determined from all mass spectrometry experiments described in ‘Materials and Methods’. Sequence coverage was based on searching peptide tandem spectra against the *Artemia franciscana* protein from NCBI, as described in the text. A: Primary sequence coverage of *Artemia franciscana* adenine nucleotide translocator protein (gi|308390607) expressed in *Artemia franciscana*. 70% Sequence coverage was obtained by identifying 210/301 residues (green) in the protein. B: Primary sequence coverage of *Artemia franciscana* adenine nucleotide translocator protein (gi|308390607) expressed in *Saccharomyces cerevisiae* (strain MWY79/15). 62% Sequence coverage was obtained by identifying 188/301 residues (green).

The apparent discrepancy between the above observation showing sensitivity of *ArAAC* expressed in yeasts to inhibition by BKA, while *Artemia* mitochondria are refractory to this poison [14], prompted us to investigate if *ArAAC* expressed in yeast is indeed identical to that expressed in *Artemia*. To answer this question, we performed mass spectrometric analysis of isolated mitochondria from strain MWY79/15 expressing the *ArAAC*, and from isolated mitochondria from embryos of *Artemia franciscana*, see Table S1 in the Supplemental Material. Samples were treated as detailed in ‘Materials and Methods’. As shown in Figure 5, we detected 62% sequence coverage of the ArAAC protein expressed in yeast mitochondria, and 70% of the ArAAC found in *Artemia*. Despite the fact that sequence coverage was not 100%, peptide fragments of ArAAC expressed in either yeasts or *Artemia* were detected near the N- and C-termini, arguing against a truncated form of *ArAAC* expressed in yeasts. By comparing the ArAAC expressed in yeasts to that expressed in *Artemia*, it could be concluded that the proteins are identical. The homologues of Sal1p, SCaMC-2 (isoform 1) and SCaMC-3 were identified in the mass spectrometric analysis of the *Artemia* mitochondria (see Excel Sheet S1 in the Supplemental Material). However, the searches of the spectra obtained by mass spectrometry were made using the mouse MitoCarta database [27]. Ongoing efforts are under way to sequence the transcriptome of *Artemia franciscana* which would be optimal for correctly identifying all proteins from the *Artemia* mitochondria peptide fragments detected by mass spectrometry.

## Discussion

We investigated the effect of heterologously expressing AAC of *Artemia franciscana* in *Saccharomyces cerevisiae*. The most important findings of this study are as follows: i) respiratory growth of yeasts expressing *ArAAC* was arrested by BKA only when *SAL1* was coexpressed; ii) fermentative growth of yeasts expressing *ArAAC* was arrested by BKA only when *SAL1* was absent, and iii) adenine nucleotide exchange mediated by ArAAC expressed in yeasts became sensitive to BKA, in a manner independent of *SAL1*.

In order to explain the sensitivity of adenine nucleotide exchange mediated by ArAAC expressed in yeasts to BKA, the challenge of reconciling two findings emerges: the first is that ArAAC expressed in *Artemia franciscana* is refractory to BKA [14], [18] and the second is that BKA binds directly on the carrier [28], [29], [reviewed in [12]], making it unlikely that an as yet to be identified protein conferring AAC sensitivity to BKA is absent in mitochondria from *Artemia franciscana*. However, the opposite scenario seems possible: that an as yet to be identified protein conferring ArAAC resistance to BKA is present in mitochondria from *Artemia franciscana*. As mentioned in the ‘Results’ section, ongoing efforts are under way to sequence the transcriptome of *Artemia franciscana* which would allow the identification of more proteins from the *Artemia* mitochondria peptide fragments. After this database is assembled, proteins interacting with ArAAC expressed in *Artemia* mitochondria can be sought.

The acquired sensitivity of ArAAC expressed in yeast to BKA outlines a limitation in a common strategy of heterologously expressing proteins in this organism for the purpose of genetic manipulation; it is neither necessary nor expected for a heterologously expressed protein to behave exactly like in its native environment. Relevant to this, the lipid environment in which AAC is embedded is a critical component for exchange activity in both yeast and mammals [30], [31], [32]. Indeed, a high sensitivity of yeast AAC2 to the cardiolipin content has been previously demonstrated [33], [34]. The lipid composition of the inner mitochondrial membrane of *Artemia* in which AAC is embedded may be very different from that in yeasts or any other organism to the extent that affords BKA resistance.

The *ArAAC-SAL1* interaction suggests a departure from what has been established for *AAC2-SAL1* interaction: in yeast cells with no *AAC2* expression the presence of *SAL1* is required for survival in glucose-containing media and *vice versa* [24]. This is because besides the respiratory function, *AAC2* exhibits an essential role for maintaining cell mitotic viability on fermentable carbon sources; co-inactivation of *SAL1* and *AAC2* leads to defects in mitochondrial translation and mitochondrial DNA (mtDNA) maintenance [35]. In the results presented above, *ArAAC*-expressing yeasts in lieu of *AAC2* and also coexpressing *SAL1* failed to exhibit respiratory growth in the presence of BKA; on the other hand, coexpression of *SAL1* in *ArAAC*-expressing yeasts in lieu of *AAC2* in glucose-containing media rescued cell growth from BKA. In the absence of BKA, expression of *ArAAC* in lieu of *AAC2* did not result in growth arrest irrespective of *SAL1* and independent from growth conditions. These results suggest that *ArAAC* restored the R function but not the V function, originally attributed to *AAC2*.

Based on the observations that Sal1p allowed yeast growth in the presence of bongkrekic acid on respiratory media, while BKA blocked ADP-ATP exchange in isolated mitochondria from the same strains, one might propose that Sal1 prevents BKA from hindering AAC in maintaining growth during respiratory conditions in a manner unrelated to adenine nucleotide exchange. This conclusion is at odds with earlier reports with yeast mutants exhibiting BKA resistance during respiration [36], in view of the presumption that these strains possessed Sal1p in their mitochondria. The reason(s) for this discrepancy is unknown; however, the exact role of Sal1p regarding yeast viability has not been unequivocally determined and is discussed below.

Sal1p mediates a Ca^2+^-dependent import of ATP-Mg from the cytosol to the mitochondria under conditions where these organelles are ATP consumers [37], [38], thereby maintaining or promoting cell survival. The mammalian homologue SCaMC (small calcium-binding mitochondrial carrier protein) also operates as a Ca^2+^-dependent ATP-Mg/P_i_ carrier. It has been shown that SCaMC-1 promotes cancer cell survival by desensitizing mitochondrial permeability transition via ATP/ADP-mediated matrix Ca^2+^ buffering [39]. SCaMC-3 is critical for the increase in oxidative phosphorylation in rodent liver mitochondria in response to glucagon and Ca^2+^-mobilizing agents, an effect that that was likely due to allowing Ca^2+^-dependent accumulation of mitochondrial adenine nucleotides [40]. Furthermore, although it has not been explicitly sought, the presence of SCaMC isoforms in the livers of transgenic mice engineered to lack their own adenine nucleotide carriers is probably the reason why these mice did not exhibit embryonic lethality [13]. However, the requisite nature of SCaMC in mammalian systems has not been attributed to maintenance of matrix adenine nucleotide levels. Likewise, in yeast it has not been established if Sal1p complements for Aac2p by means of transporting adenine nucleotides, hence the opposing conclusions by the groups of Chen and Kolarov [35], [20]. Nonetheless, yeast as a model system enjoys greater versatility because its growth can be examined during fermenting (V function) versus respiratory (R function) conditions. With such a comparison, we addressed the contribution of *SAL1* and *AAC2*. The availability of our yeast strains expressing *ArAAC* in lieu of *AAC2* and the effects it exhibits in relation to *SAL1* may help to elucidate the role of *SAL1* to cell survival.

Finally, mindful that mitochondria obtained from *Artemia franciscana* do not exhibit the Ca^2+^-induced PTP [8], it would be desirable to test yeast mitochondria expressing *ArAAC* for Ca^2+^-induced PTP. Unfortunately, *Saccharomyces cerevisiae* do not express the mitochondrial calcium uniporter [41], [42], [43], even though they exhibit a channel with characteristics reminiscent of PTP, known as ‘yeast mitochondrial unselective channel’ (YMUC) [44].

## Materials and Methods

### Strains and Genotypes

The *S. cerevisiae* strains and their genotypes are listed in Table 1.

### Construction of Yeast Strains

To construct *AAC1* and *AAC3* deletions in MR6 background the *aac1::KanMX4* and *aac3::KanMX4* cassettes were amplified by PCR using as templates total DNA isolated from strains bearing deletions of *AAC1* and *AAC3* genes from the EUROSCARF collection. MR6 strain was transformed with the deletion cassettes as described previously [45] and transformants were selected on YPGA medium supplemented with 200 µg/ml geneticin G418. The correct integration of the cassettes was verified by PCR using AAC1Ver, AAC3Ver and KanMX-Up primers, and by phenotypic analysis. The RKY67-1C double *aac1::KanMX4 aac3::KanMX4* strain was constructed by crossing the single deletion strains *aac1::KanMX4* and *aac3::KanMX4,* sporulation of the diploid *AAC1*/*aac1::KanMX4 AAC3/aac3::KanMX4* strain followed by tetrad dissection. To integrate *ArAAC* into yeast genome the *ArACC* gene was first amplified with primers ArAnt F and ArAnt R (all primers are listed in Table 2) using cDNA from reverse transcribed total *Artemia franciscana RNA* as template and cloned into TOPO-TA Cloning Vector. The ArACC cassette for integration into yeast DNA was constructed by multiple PCR reactions; all cloning steps were verified by sequencing [46]. The cassette included *ArACC-HA* and *HphNTI* genes flanked by yeast *ACC2* gene promoter and terminator sequences, see Figure 1. The *HA*, *HphNTI*, *AAC2* gene promoter and terminator sequences were amplified separately using pYM16 or total DNA from yeast as templates, respectively. Before the *HphNTI* gene the sequences of ADH terminator and TEF promoter are present [46]. The *AAC2* gene promoter was then fused to *HA* by one PCR reaction (product A), *HA* was fused to ADH terminator and *HphNTI* gene by a second PCR reaction (product B) and *HA* and *HphNTI* gene were fused to *AAC2* gene terminator in a third PCR reaction (product C). The final PCR product was assembled using products A, B and C as templates and cloned into pGEM T-easy vector resulting in plasmid pMW77/11. The EcoRV restriction site was engineered before the *HA* tag sequence. The *ArACC* gene was amplified from the *pCR 2.1*-*TOPO* vector (Invitrogen) using primers 9upANT and 10upANT, introducing EcoRV restriction sites. The 915-bp product digested by EcoRV was cloned into EcoRV site of pMW77/11 resulting in pMW83 plasmid. The integration cassette was cut off from pMW83 by XbaI enzyme and used to transform RKY67-1C strain. Transformants were selected on YPGA medium supplemented with 300 µg/ml hygromycin B. Homologous integration of *ArACC* in MWY79/15 strain was verified by PCR using primers AAC2veri and AAC2lov and by phenotypic analysis. To construct *sal1*::*NatMX4* deletion, the *SAL1* coding sequence and its promoter and terminator region (−193 bp upstream and 226 bp downstream) was PCR-amplified using primers Sal1-Up and Sal1-Low. The PCR product was cloned into pJet vector resulting in pMW100/6 plasmid. The construct *sal1Δ::NatMX4* gene and the *Pvu*II/*Eco*RV fragment from plasmid pAG25 [47] including *NatMX4* drug resistance gene was cloned into *Kpn*I blunt-ended with Klenow and *Eco*RV sites of *SAL1* gene on pMW100/6 plasmid resulting in pMW105/5 plasmid. A linear DNA fragment including *sal1Δ::NatMX4* cassette was cut off from this plasmid and introduced by transformation into RKY67-1C and MWY79/15 strain bearing the wild type yeast *AAC2* gene on a Yep352 URA3 plasmid. Transformants were selected on YPGA medium supplemented with nourseothricin (nat), 100ug/ml. Homologous integration of *sal1::NatMX4* cassette in MWY84/3 and MWY83/5 strains was verified by PCR using the primers SalVerif and SalR and by phenotypic analysis.

**Table 2:** Oligonucleotides used in this work.

Oligonucleotide	Sequence	Goal
AAC1Up	GCGACTCAGCGTACGTAGG	*AAC1* deletion
AAC1Low	CGTAAACGGTTCCCTTCCGC	*AAC1* deletion
AAC1Ver	ACTACATGCACGAGGCCTTGGC	*AAC1* deletion verification
AAC3Up	TTTCATTGTTTGGTTGCCTTC	*AAC3* deletion
AAC3Low	ATCCAACCATCTGAAAGCCG	*AAC3* deletion
AAC3Ver	GCTTCCAATGGCCTCCTCACCG	*AAC3* deletion verification
KanMX-Up	GGATGTATGGGCTAAATGTA	*AAC3* deletion verification
ArAnt F:	ATG GCA ACC AAG AAG CAA GCG G	ArACC amplification
ArAnt R:	CTA ACC AAT AAG CAC TTT AAG CTC	ArACC amplification
1 ANTcon	GTCTAGAAACATCACGATGC ACGAGCACTGT	*ACC2* promoter amplification
2 ANTcon	GTAAGCGTAATCTGGAACATCGTATGGGTAGATATCGGC TATTTGCTTATATGTATGTTAATG 3′	*ACC2* promoter amplification
3 ANTcon	CAT TAA CAT ACA TAT AAG CAA ATA GCCGAT ATC TAC CCA TAC GAT GTT CCA GAT TAC GCT TACCC	HA tag encodingsequence amplification
4 ANTcon	CATAAATCATAAGAAATTCGCTTATTTAGAAGTGCTAA GCGTAATCTGGAACATCGTATGGGTA	HA tag encodingsequence amplification
5 ANT con	CCCATACGATGTTCCAGATTACGCTTAGCACTTCTAAAT AAGCGAATTTCTTATGATTTATG	*HphNTI* gene sequencesamplification
6 ANT con	GATTAAGAATCAAGCCAGATTAGACTTATTCCTTT GCCCTCGGACGAGTGCTG	*HphNTI* gene sequencesamplification
7 ANT con	CAGCACTCGTCCGAGGGCAAAGGAATAAGTCTA ATCTGGCTTGATTCTTAATC	*ACC2* terminator amplification
8 ANT con	GTCTAGACGGCACAAAGAG TGATAGACCTATTTGGC	*ACC2* terminator amplification
9upANT	CGATATCATGGCAACCAA GAAGCAAGCGGATCCCCTC	*ArACC* gene amplification
10forANT	CGATATCACCAATAAGC ACTTTAAGCTCGTCATAGAA	*ArACC* gene amplification
AAC2veri	AACATCACGATGCACGAGCACTG	Cassette integration verification
AAC2low	GAGTGATAGACCTATTTGGCGGTG	Cassette integration verification
Sal1-Up	CAGGCAATTAACCTTGTGTTTCTGACG	*SAL1* gene amplification
Sal1-Low	CCACAAACCGCAGCAGCGGTTTATAA	*SAL1* gene amplification
SalVerif	GTTCCAACTTGGGCATTTTTCAGAG	*Sal1*::NatMX4 integrationverification
SalR	CCACAAACCGCAGCAGCGGTTTATAA	*Sal1*::NatMX4 integrationverification

Restriction sites for XbaI and EcoRI sites are underlined.

### Growth and Media

The media used for yeast growth were: YPGA (1% (w/v) yeast extract, 1% (w/v) peptone, 2% (w/v) glucose, and 40 mg/liter adenine); N3 (1% (w/v) yeast extract, 1% (w/v) peptone, 2% (w/v) glycerol; YPGALA (1% (w/v) yeast extract, 1% (w/v) peptone, 2% (w/v) galactose, and 40 mg/liter adenine); W0 (0.17% (w/v) yeast nitrogen base without amino acids, 2% (w/v) glucose, amino acids, adenine and uracil (media manufactured by Sunrisescience, Erpent, Belgium). Solid media contained 2% (w/v) agar. The YPGA or YPGLY BKA plates additionally contained 50 mM sodium citrate, BKA 1 µM (if yeasts were grown on glucose) or 0.02 µM (if yeasts were grown on glycerol) where indicated, and the pH 4 was adjusted by 1 M citric acid. For mitochondrial isolation yeast cells were cultured to the mid-logarithmic phase on YPGALA medium (OD = 4) at 28°C while shaking at 180 rpm. The 5-FOA plates contained W0 medium supplemented with all required amino acids and 0,1% 5-fluooroorotic acid.

### Yeast Mitochondrial Isolation

Mitochondria from the yeast strains were prepared as described previously [48]. Typical yield was ∼20 mg of mitochondria from 2 liters of yeast cultures (OD = 4). Protein was determined by the method of Lowry.

### Isolation of Mitochondria from Artemia Franciscana

No permits were required for the described study, which complied with all relevant regulations. Mitochondria from embryos of *Artemia franciscana* were prepared as described elsewhere, with minor modifications [2]. Dehydrated, encysted gastrulae of *Artemia franciscana* were obtained from Salt Lake, Utah through Artemia International LLC (Fairview, Texas 75069, USA) and stored at 4°C until used. Embryos (15 g) were hydrated in 0.25 M NaCl at room temperature for at least 24 h. After this developmental incubation, the embryos were dechorionated in modified antiformin solution (1% hypochlorite from bleach, 60 mM NaCO3, and 0.4 M NaOH) for 30 min, followed by a rinse in 1% Na^+^-thiosulfate (5 min) and multiple washings in ice-cold 0.25 M NaCl as previously described [49]. After the embryos were filtered through filter paper, ∼10 g were homogenized in ice-cold isolation buffer consisting of 0.5 M sucrose, 150 mM KCl, 1 mM EGTA, 0.5% (wt/vol) fatty acid-free BSA, and 20 mM K^+^-HEPES, pH 7.5, using a glass-Teflon homogenizer at 850 rpm for ten passages. The homogenate was centrifuged for 10 min at 300 g at 4°C, the upper fatty layer of the supernatant was aspirated and the remaining supernatant was centrifuged at 11,300 g for 10 min. The resulting pellet was gently resuspended in the same buffer, avoiding the green core. The green core was discarded, and the resuspended pellet was centrifuged again at 11,300 g for 10 min. The final pellet was resuspended in 0.4 ml of ice-cold isolation buffer consisting of 0.5 M sucrose, 150 mM KCl, 0.025 mM EGTA, and 20 mM K^+^-HEPES, pH 7.5 and contained ∼80 mg protein/ml.

### Determination of Mitochondrial Membrane Potential (m)

m of isolated yeast mitochondria was estimated as described previously [50], by monitoring the quenching of rhodamine 123 fluorescence (0.5 µM) using a λ_ex_ of 485 nm and a λ_em_ of 533 nm at an acquisition rate of 10 Hz with a Cary Eclipse Fluorescence Spectrophotometer (Agilent Technologies, Santa Clara, CA, USA) during constant stirring. 1 mg of yeast mitochondria was added in a 2 ml buffer consisting of 0.65 M mannitol, 0.36 mM EGTA, 1 mM MgCl_2_, 15 mM Trizma, 11 mM NaH_2_PO_4_, and 10 mM malate; the pH was set in a range of 7.1–7.4 as detailed in the Results section. All experiments were performed at 28°C.

### Determination of Citrate Synthase Activity

Citrate synthase activity for the fractions obtained during mitochondrial and mitoplast isolations was performed as detailed in [51]. Citrate synthase specific activity is expressed as nmol of DTNB (5,5′-dithiobis-(2-nitrobenzoic acid) reduced to NTB (2-nitro-5-thiobenzoate) per min per mg of protein. Mitoplasts were generated by subjecting 10 mg of mitochondria in a 2 ml buffer composed of 20 mM Tris pH 6.8 for 30 min, in the presence of 0.05% digitonin. After spinning at 10,000 g for 10 min, the supernatant was removed, and the pellet consisting of mitoplasts was resuspended in 0.1 ml of the same buffer.

### Western Blotting

SDS-PAGE was performed according to Laemmli [52], with the modifications elaborated in [53]. Monoclonal anti-HA antibody (16B12) against HA tag (Berkeley Antibody Company, Richmond, CA, USA) was used at a 1∶5,000 dilution. Rabbit polyclonal anti-porin was used at a 1∶10,000 dilution. Immunoreactivity was detected in the nitrocellulose papers using peroxidase-linked secondary antibodies (1∶10,000, DAKO, Agilent Technologies) and enhanced chemiluminescence detection reagent (Immobilon Western Chemiluminescent HRP Substrate, Millipore).

### Mass Spectrometry: Yeast Mitochondria

2 µl of yeast mitochondria (6 mg/ml) containing 5 pmol beta-lactoglobulin as an internal standard was incubated for 30 min at 60°C with 1 µl reagent mixture containing 0.33 w/w% RapiGest SF and 33 mM dithiothreitol in a total volume of 20 µl. This was followed by alkylation for 30 min in the dark at room temperature in the presence of 1 µl 200 mM iodoacetamide and 5 µl 200 mM NH_4_HCO_3._ Digestion was performed either by trypsin (1 µl, 20 µM) at 37°C for 90 min or 180 min or by chymotrypsin (1 µL, 39 µM) at 37°C for 90 min or overnight. Digestion was quenched by 1 µl formic acid (30 min at 37°C) and the reaction product was centrifuged at 17,000 g for 10 min. The sample was diluted two times prior to analysis and a 2 µL sample was injected on to the capillary LC column. LC–MS/(MS) experiments were carried out using a nanoflow UPLC system (nanoAcquity UPLC, Waters, Milford, MA, USA) coupled to a Q-TOF Premier mass spectrometer (Waters, Milford, MA, USA). Before separating the peptides on a reverse phase analytical column (C18, 75 µm i.d. × 150 mm, 1.7 µm BEH300 particles, Waters, Milford, MA, USA), samples were desalted online on a Symmetry C18 trap column (180 µm i.d. × 20 mm, Waters, Milford, MA, USA). A gradient was applied using a ﬂow rate of 450 nanol/min and column temperature 55°C for 100 or 200 min, as described previously [54] using aqueous and acetonitrile-containing solvents, both in the presence of 0.1% formic acid. Peptides were identified by tandem mass spectrometry in two separate data dependent acquisition modes (DDA). In the first case 2 sec cycles were used, consisting of a full scan spectrum (m/z: 500–1999) and MS/MS spectra of the three most abundant ions. In the second case 3 sec cycles were applied, consisting of a full scan spectrum (m/z: 400–1500) and MS/MS spectra of the three most abundant ions included in the predefined inclusion list. The inclusion list contained the masses of the expected tryptic or chymotryptic peptides of ArAAC. Ar collision gas was used in the tandem mass spectrometry measurements. ProteinLynx Global Server v.2.3 (Waters, Milford, MA, USA) was used to process data of DDA experiments. Mascot Server version 2.2 (Matrix Science, London, UK) was used to analyze samples searching against *Artemia franciscana* taxonomy assuming digestion enzymes trypsin or chymotrypsin and allowing for two missed cleavages. Iodoacetamide derivatives of cysteines and oxidation of methionines were specified as fixed and variable modifications, respectively. Fragment ion mass tolerance of 0.15 Da and a parent ion tolerance of 50 ppm were used and peptides were considered if they could be established at greater than 95.0% probability. To identify further peptide fragments, additional error-tolerant Mascot searches were performed against *Artemia franciscana* taxonomy.

### Sample Preparation for Artemia Franciscana Mitochondria Lysed in RIPA Buffer

*Artemia franciscana* mitochondria in RIPA (radioimmunoassay) buffer were thawed and centrifuged at 16,000 g for 10 min. The pellet was solubilized in 5 M urea, 2 M thiourea, 40 mM DTT and 0.1% SDS, and both the pellet and the supernatant were cleaned up using the GE Healthcare 2-D Clean-Up Kit per the manufacturer’s instructions (GE Healthcare Bio-Sciences Corp., Piscataway NJ). After the clean up, the pellets were extracted with 100 mM ammonium bicarbonate, pH 7.8 and sonicated for 10 min in a bath sonicator. After centrifugation, the extraction and sonication steps were repeated on pellets still remaining. Supernatants were combined and the protein concentration was determined using the Pierce 660 nm assay as per the manufacturer’s instructions (Pierce, Rockford IL). Three 60 g protein portions were electrophoresed by SDS-PAGE using a 7 cm 10% Mini-Protean TGX gel from BioRad (BioRad, Hercules CA) and stained with BioSafe Coomassie Blue (BioRad, Hercules CA). The gel lanes were digested with trypsin following reduction with DTT and alkylation with iodoacetamide, followed by peptide extraction from the gel lane [55]. The peptide digest was cleaned up using Spec-C18 cartridges (Varian, Lake Forest, CA).

### LC-LC-MS/MS Analysis of the RIPA-lysed Mitochondria

For LC-LC-MS/MS analysis, a microbore HPLC system (Surveyor, Thermo Fisher Scientific, San Jose, CA) was used with separate strong cation exchange (SCX) and reversed phase (RP) columns: a 100 µm I.D. capillary packed with 3.5 cm of 5 µm PolySulfoethyl-Asp strong cation exchanger (PolyLC Inc., Columbia, MD) and a separate 100 µm I.D. capillary packed with 7.5 cm of 5 µm Zorbax Eclipse XDB-C18 material (Agilent, Santa Clara, CA). Peptides were acidified using trifluoroacetic acid and 30 g was manually loaded by pressure packing onto the SCX column. Peptides were eluted at 400 nl/min by a reverse phase gradient using Buffer A (water/0.1% formic acid), Buffer B (acetonitrile/0.1% formic acid), preceded by a salt bump using Buffer C (250 mM ammonium acetate), and Buffer D (1.5 M ammonium acetate). Twelve steps were then performed as follows: (step 1) 0% C with an RP gradient of 5–50% B over 90 minutes followed by a column clean-up of 5 minutes using 50–98% B and an equilibration of 20 min with 5% B. (steps 2–11) X% C (where X = 10–100% C increased in increments of 10%; the remaining % was Buffer A) loaded over 4 min and then washed with 5% B for 7 min followed by a RP gradient of 5–50% B over 60 min. Each RP gradient was followed by a column clean-up of 5 min using 50–98% B and an equilibration of 20 min with 5% B. Step 12∶50% D loaded over 4 min and then washed with 5% B for 7 min followed by a gradient of 5–50% B over 60 min. The flow rate was 1000 nl/min for the 7-minute washes following each salt bump and for each final 5% B equilibration step. Peptides were directly sprayed into a ThermoFinnigan LTQ linear ion trap mass spectrometer (Thermo Fisher Scientific, San Jose, Ca) using a custom-built nanoelectrospray ionization source. Electrospray voltage of 2.0 kV was applied using a gold electrode via a liquid junction upstream of the column. Spectra were scanned over the range 400–1500 atomic mass units (amu). Automated peak recognition, dynamic exclusion (45 seconds), and daughter ion scanning of the top seven most intense ions were performed using the Xcalibur v 1.4 SR1 data system (Thermo Fisher Scientific, San Jose CA) [56]. The LC-LC-MS/MS analysis was repeated twice for a total of three replicates and the data were combined. Tandem MS spectra of peptides were analyzed with TurboSEQUEST™ v 3.1, a program that allows the correlation of experimental tandem MS data with theoretical spectra generated from known protein sequences [57]. The peak list for the search was generated by Bioworks 3.1 (Thermo Fisher Scientific, San Jose, CA) considering fully tryptic peptides with up to two missed cleavages. Iodoacetamide derivatives of cysteines and oxidation of methionines were specified as variable modifications. Parent peptide mass error tolerances were set at 1.5 amu and fragment ion mass tolerance set at 0.5 amu during the search. Preliminary peptide identifications were made using the following Xcorr filters: peptide precursor ions with a +1 charge having a Xcorr >1.8, +2 Xcorr >2.5 and +3 Xcorr >3.5. A deltaCn score >0.08 was also used as filtering criteria for reliable matched peptide identification [58], [59]. Tandem mass spectra were searched against mouse genes encoding proteins with strong support of mitochondrial localization downloaded from the Broad Institute (http://www.broadinstitute.org/pubs/MitoCarta/index.html), on July 27, 2012. At the time of the search this Mouse MitoCarta protein database contained 1,173 entries. Additionally, the tandem mass spectra were searched against Crustacea proteins downloaded from NCBI on July 25, 2012 and appended with common contaminant proteins (eg., human keratins, trypsin, etc). At the time of the search this custom protein database contained 108,788 entries. The results were also validated using the search engine X!Tandem [60], and displayed with Scaffold v 3.6.1 (Proteome Software Inc., Portland OR), a program that relies on various search engine results (i.e: Sequest, X!Tandem, MASCOT) and which uses Bayesian statistics to reliably identify more spectra [61].

### Sample Preparation for Artemia Franciscana Mitochondria Lysed by Freeze/Thaw in Water

SDS was added to the lysate at a final concentration of 0.1% and the lysate was sonicated for 5 sec using a probe sonicator. Proteins were precipitated with acetone. After centrifugation at 16,000 g for 10 min the topmost lipid layer and the supernatant were removed and discarded. The pellet was extracted with 8 M urea/1 M guanadine HCl, sonicated as described above, and subjected to a round of freeze/thawing. After centrifugation the supernatant was saved and the pellet was extracted with 8 M urea then centrifuged and the supernatant was removed and saved. The combined supernatants were assayed for protein concentration using the Pierce 660 nm reagent as per the manufacturer’s instructions (Pierce, Rockford IL). The pellets from the processing steps described above were dissolved in Laemmli sample buffer, combined, and assayed for protein concentration as described previously. One mg portions of the supernatant fraction were either delipidated ([62] or cleaned up using the GE Healthcare 2-D Clean-Up Kit as per the manufacturer’s instructions (GE Healthcare, Piscataway NJ). The samples were fractionated by SDS-PAGE; 10 g each of the delipidated sample, the kit-processed sample, and the pellets was loaded on a 15% acrylamide Criterion gel (BioRad, Hercules, CA), and after electrophoresis the gel was stained with BioSafe Coomassie Blue (BioRad, Hercules CA). Each gel lane was cut into 8 sections and the sections were digested with trypsin following reduction with DTT and alkylation with iodoacetamide, followed by peptide extraction from the gel sections [55].

### LC MS/MS Analysis of the Freeze/Thaw Lysed Artemia Franciscana Mitochondria

LC-MS/MS analysis of trypsin-digested gel sections was carried out using a LTQ Orbitrap Velos mass spectrometer (Thermo Fisher Scientific, San Jose, CA) equipped with an Advion nanomate ESI source (Advion, Ithaca, NY), following ZipTip C18 sample clean up according to the manufacturer’s instructions (Millipore, Billerica, MA). Peptides were eluted from a C18 precolumn (100-µm id×2 cm, Thermo Fisher Scientific) onto an analytical column (75-µm ID × 10 cm, C18, Thermo Fisher Scientific) using a 5% hold of solvent B (acetonitrile, 0.1% formic acid) for 5 min, followed by a 5–7% gradient of solvent B over 5 min, 7–15% gradient of solvent B over 45 min, 15–35% gradient of solvent B over 60 min, 35–40% gradient of solvent B over 28 min, 40–85% gradient of solvent B over 5 min, 85% hold of solvent B for 10 min and finally a return to 5% in 1 minute and another 10 minute hold of 5% solvent B. All flow rates were 400 nl/min. Solvent A consisted of water and 0.1% formic acid. Replicate runs were also performed using a shorter 125 minute RP gradient (5% solvent B hold for 10 min, 5–20% gradient of solvent B over 65 min, followed by a 20–35% gradient of solvent B over 25 min, 35% solvent B hold for 9 min, 35–95% gradient of solvent B over 5 min, and finally by a 95% solvent B hold for another 5 minutes). Data dependent scanning was performed by the Xcalibur v 2.1.0 software [56] using a survey mass scan at 60,000 resolution in the Orbitrap analyzer scanning mass/charge (m/z) 400–1600, followed by collision-induced dissociation (CID) tandem mass spectrometry (MS/MS) of the 14 most intense ions in the linear ion trap analyzer. Precursor ions were selected by the monoisotopic precursor selection (MIPS) setting with selection or rejection of ions held to a +/−10 ppm window. Dynamic exclusion was set to place any selected m/z on an exclusion list for 45 seconds after a single MS/MS. All MS/MS spectra were searched against the protein databases as described above using Thermo Proteome Discoverer 1.3 (Thermo Fisher Scientific) considering fully tryptic peptides with up to two missed cleavages. Iodoacetamide derivatives of cysteines and oxidation of methionines were specified as variable modifications. Proteins were identified at 99% confidence with XCorr score cut-offs [59] as determined by a reversed database search. The protein and peptide identification results were also visualized with Scaffold v 3.6.1 (Proteome Software Inc., Portland OR), a program that relies on various search engine results (*i.e*.: Sequest, X!Tandem, MASCOT) and which uses Bayesian statistics to reliably identify more spectra [61]. Proteins were accepted that passed a minimum of two peptides identified at 95% peptide confidence and 99.9% protein confidence by the Peptide and Protein Profit algorithms, respectively, within Scaffold.

## Supporting Information

Table S1 ArAAC peptides identified by various mass-spectrometric methods. Samples were purified Artemia franciscana mitochondria treated by various approaches as described in the headers of the tables and detailed in Materials and Methods.
(PDF)

Excel Sheet S1 Mass spectrometric analysis of *Artemia* mitochondria. Homologues of identified Sal1p, SCaMC-2 (isoform 1) and SCaMC-3 are listed in six sheets, each representing the results of an independent experiment.
(PDF)
