# Supplementary material for: The Suppressor of AAC2 Lethality SAL1 Modulates Sensitivity of Heterologously Expressed Artemia ADP/ATP Carrier to Bongkrekate in Yeast
Source: PLoS One. 2013 Sep 20;8(9):e74187. doi: 10.1371/journal.pone.0074187 (PMC3779231; doi:10.1371/journal.pone.0074187)
Supplement: Table S1 — ArAAC peptides identified by various mass-spectrometric methods. Samples were purified Artemia franciscana mitochondria treated by various approaches as described in the headers of the tables and detailed in Materials and Methods. (PDF) [file pone.0074187.s001.pdf]

Supporting Table S1

ArAAC peptides identified by LC-MS/MS on the LTQ Orbtrap Velos from gel bands of *Artemia franciscana* purified mitochondria in RIPA as described in Materials and Methods

Sequence	Prob*	SEQUEST XCorr	SEQUEST deltaCn	Actual Mass	Charge	Delta PPM	Start	Stop
(R)AAYFGFYDTVR(G)	95%	3.0163724	0.25418755	1,308.62	1	1.451	193	203
(R)AAYFGFYDTVR(G)	95%	3.3941338	0.40535244	1,308.61	1	0.7058	193	203
(R)AAYFGFYDTVR(G)	95%	2.9873574	0.43730178	1,308.61	2	0.5557	193	203
(R)AAYFGFYDTVR(G)	95%	3.3862085	0.4345924	1,308.62	2	1.395	193	203
(R)AAYFGFYDTVR(G)	95%	3.1920612	0.4688232	1,308.61	2	0.3693	193	203
(R)AAYFGFYDTVR(G)	95%	3.268941	0.4965996	1,308.62	2	1.022	193	203
(R)AAYFGFYDTVR(G)	95%	3.330099	0.49018052	1,308.62	2	0.8353	193	203
(R)AAYFGFYDTVR(G)	95%	3.3456209	0.5133169	1,308.61	2	0.5557	193	203
(R)AAYFGFYDTVR(G)	95%	3.4890506	0.5419574	1,308.62	2	1.115	193	203
(R)AKGDMMYK(G)	95%	2.2146318	0.2990061	942.4305	2	0.04847	247	254
(R)AKGDMMYK(G)	95%	2.4631445	0.33896124	942.4288	2	-1.763	247	254
(R)AKGDMMYK(G)	95%	2.0648665	0.5011398	942.4295	2	-1.051	247	254
(R)AKGDMMYK(G)	95%	2.6334202	0.51865804	942.4285	2	-2.086	247	254
(R)AKGDMmYK(G)	95%	2.3655164	0.5439363	958.4246	2	-0.8231	247	254
(R)AKGDmMYK(G)	95%	2.6210814	0.50192755	958.425	2	-0.4414	247	254
(R)AKGDmMYK(G)	95%	2.1529179	0.60710883	958.4245	2	-0.8867	247	254
(R)AKGDmMYK(G)	95%	2.5997615	0.56987196	958.4248	2	-0.5686	247	254
(R)AKGDmmYKGTLDcWAK(I)	95%	2.4847996	0.37556446	1,905.84	3	-0.3162	247	262
(R)AKGDmmYKGTLDcWAK(I)	95%	2.562325	0.37055445	1,905.84	3	0.06788	247	262
(K)DFmAGGISAAVSK(T)	95%	2.4548216	0.47839406	1,268.61	1	-0.2562	15	27
(K)DFmAGGISAAVSK(T)	95%	2.648362	0.4559059	1,268.61	1	0.513	15	27
(K)DFmAGGISAAVSK(T)	95%	2.796475	0.48568726	1,268.61	1	0.7053	15	27
(K)DFmAGGISAAVSK(T)	95%	2.6931791	0.5078229	1,268.61	1	0.1284	15	27
(K)DFmAGGISAAVSK(T)	95%	2.9922383	0.5659426	1,268.61	1	1.571	15	27
(K)DFMAGGISAAVSK(T)	95%	4.058552	0.47833154	1,252.61	1	0.6504	15	27
(K)DFMAGGISAAVSK(T)	95%	4.513816	0.45082054	1,252.61	1	0.7478	15	27

(K)DFmAGGISAAVSK(T)	95%	3.4210382	0.5965188	1,268.61	1	1.378	15	27
(K)DFmAGGISAAVSK(T)	95%	3.872974	0.5578232	1,268.61	1	0.2245	15	27
(K)DFmAGGISAAVSK(T)	95%	3.6262233	0.58254534	1,268.61	1	0.513	15	27
(K)DFmAGGISAAVSK(T)	95%	4.15499	0.6473489	1,268.61	1	0.7053	15	27
(K)DFMAGGISAAVSK(T)	95%	3.8885736	0.53256094	1,252.62	2	3.123	15	27
(K)DFMAGGISAAVSK(T)	95%	3.6931484	0.5497092	1,252.62	2	2.538	15	27
(K)DFMAGGISAAVSK(T)	95%	4.3239393	0.4865814	1,252.61	2	0.591	15	27
(K)DFMAGGISAAVSK(T)	95%	4.8879204	0.51132524	1,252.61	2	0.6883	15	27
(K)DFMAGGISAAVSK(T)	95%	4.9311256	0.5265583	1,252.61	2	0.591	15	27
(K)DFMAGGISAAVSK(T)	95%	4.7758336	0.5394209	1,252.61	2	1.273	15	27
(K)DFmAGGISAAVSK(T)	95%	4.307295	0.5833172	1,268.61	2	0.7428	15	27
(K)DFMAGGISAAVSK(T)	95%	4.953354	0.5356949	1,252.61	2	0.1041	15	27
(K)DFmAGGISAAVSK(T)	95%	4.3853593	0.5800293	1,268.61	2	0.7428	15	27
(K)DFmAGGISAAVSK(T)	95%	4.265657	0.60480124	1,268.61	2	-0.3149	15	27
(K)DFMAGGISAAVSK(T)	95%	4.8505282	0.56116563	1,252.61	2	0.4936	15	27
(K)DFMAGGISAAVSK(T)	95%	5.2380495	0.5455726	1,252.61	2	0.3962	15	27
(K)DFmAGGISAAVSK(T)	95%	4.61892	0.6163612	1,268.61	2	1.416	15	27
(K)DFmAGGISAAVSK(T)	95%	4.807017	0.6026257	1,268.61	2	-0.6994	15	27
(K)DFMAGGISAAVSK(T)	95%	5.2576137	0.58525354	1,252.61	2	0.3962	15	27
(K)DFmAGGISAAVSK(T)	95%	4.5021496	0.64112747	1,268.61	2	-0.7956	15	27
(K)DFmAGGISAAVSK(T)	95%	4.676969	0.62932795	1,268.61	2	-0.3149	15	27
(K)DFmAGGISAAVSK(T)	95%	4.3004966	0.661033	1,268.61	2	-0.02642	15	27
(K)DFmAGGISAAVSK(T)	95%	4.8959613	0.6225434	1,268.61	2	0.6466	15	27
(K)DFmAGGISAAVSK(T)	95%	4.6174526	0.6531184	1,268.61	2	-0.8917	15	27
(K)DFmAGGISAAVSK(T)	95%	4.622409	0.6531431	1,268.61	2	-0.6033	15	27
(K)DFmAGGISAAVSK(T)	95%	4.5799417	0.657482	1,268.61	2	0.4543	15	27
(K)DFmAGGISAAVSK(T)	95%	5.024012	0.63501257	1,268.61	2	0.4543	15	27
(K)DFmAGGISAAVSK(T)	95%	4.6856766	0.66337454	1,268.61	2	-0.02642	15	27
(K)DFmAGGISAAVSK(T)	95%	4.841867	0.6548432	1,268.61	2	-0.7956	15	27
(K)DFmAGGISAAVSK(T)	95%	5.165389	0.6459415	1,268.61	2	0.262	15	27
(K)DVFKQIFmSGVDKK(T)	95%	3.1467474	0.48520455	1,656.85	3	-0.05332	97	110
(K)DVFKQIFmSGVDKK(T)	95%	3.9446192	0.4598254	1,656.86	3	0.8303	97	110

(R)EFSGLGNcLVK(I)	95%	2.4741926	0.26260996	1,222.60	1	1.494	157	167
(R)EFSGLGNcLVK(I)	95%	2.9244885	0.21546887	1,222.60	2	0.1366	157	167
(R)EFSGLGNcLVK(I)	95%	2.6928163	0.27222866	1,222.60	2	-0.5617	157	167
(R)EFSGLGNcLVK(I)	95%	2.4502087	0.31220648	1,222.60	2	0.5357	157	167
(R)EFSGLGNcLVK(I)	95%	2.87748	0.27154154	1,222.60	1	1.395	157	167
(R)EFSGLGNcLVK(I)	95%	3.08266	0.3419584	1,222.60	1	2.093	157	167
(R)EFSGLGNcLVK(I)	95%	2.6398022	0.29518142	1,222.60	2	0.9347	157	167
(R)EFSGLGNcLVK(I)	95%	3.082898	0.286313	1,222.60	1	1.395	157	167
(R)EFSGLGNcLVK(I)	95%	2.6521163	0.30387732	1,222.60	2	0.1366	157	167
(R)EFSGLGNcLVK(I)	95%	2.6242316	0.32306674	1,222.60	2	0.5357	157	167
(R)EFSGLGNcLVK(I)	95%	3.2675056	0.29683352	1,222.60	1	1.295	157	167
(R)EFSGLGNcLVK(I)	95%	3.0318687	0.28877845	1,222.60	2	1.234	157	167
(R)EFSGLGNcLVK(I)	95%	3.05495	0.31823164	1,222.60	2	2.132	157	167
(R)EFSGLGNcLVK(I)	95%	3.0147083	0.38956228	1,222.60	1	1.594	157	167
(R)EFSGLGNcLVK(I)	95%	2.7084417	0.31962645	1,222.60	2	-0.1627	157	167
(R)EFSGLGNcLVK(I)	95%	2.6949575	0.36707097	1,222.60	2	1.533	157	167
(R)EFSGLGNcLVK(I)	95%	2.6423342	0.35743362	1,222.60	2	0.8349	157	167
(R)EFSGLGNcLVK(I)	95%	2.729069	0.3446595	1,222.60	2	0.1366	157	167
(R)EFSGLGNcLVK(I)	95%	3.3132672	0.2865033	1,222.60	2	0.03684	157	167
(R)EFSGLGNcLVK(I)	95%	2.9934254	0.33810744	1,222.60	2	0.3361	157	167
(R)EFSGLGNcLVK(I)	95%	2.812743	0.3806756	1,222.60	2	0.2364	157	167
(R)EFSGLGNcLVK(I)	95%	2.929068	0.38352954	1,222.60	2	1.134	157	167
(R)EFSGLGNcLVK(I)	95%	3.1538224	0.35172182	1,222.60	2	0.6354	157	167
(R)EFSGLGNcLVK(I)	95%	2.96593	0.3791198	1,222.60	2	0.5357	157	167
(R)EFSGLGNcLVK(I)	95%	3.047904	0.35797697	1,222.60	2	-0.06292	157	167
(R)EFSGLGNcLVK(I)	95%	3.017869	0.3844685	1,222.60	2	-0.06292	157	167
(R)EFSGLGNcLVK(I)	95%	3.402223	0.35413593	1,222.60	2	0.4359	157	167
(R)EFSGLGNcLVK(I)	95%	3.0564399	0.39888725	1,222.60	2	0.2364	157	167
(R)EFSGLGNcLVK(I)	95%	3.2274601	0.4521215	1,222.59	2	-8.942	157	167
(R)EFSGLGNcLVK(I)	95%	3.181719	0.40725976	1,222.60	2	0.8349	157	167
(K)EQGVLSFWR(G)	95%	2.6354656	0.18282624	1,120.57	1	1.53	68	76
(K)GAAEREFSGLGNcLVK(I)	95%	2.0751126	0.23814724	1,706.84	3	-0.6008	152	167

(K)GAAEREFSGLGNcLVK(I)	95%	4.248781	0.27756792	1,706.85	2	4.518	152	167
(K)GAAEREFSGLGNcLVK(I)	95%	3.78152	0.24001144	1,706.84	2	0.8009	152	167
(K)GAAEREFSGLGNcLVK(I)	95%	3.5886607	0.29286268	1,706.84	2	0.6579	152	167
(K)GAAEREFSGLGNcLVK(I)	95%	3.6671538	0.27338696	1,706.84	2	0.4435	152	167
(K)GAAEREFSGLGNcLVK(I)	95%	3.3048964	0.21739435	1,706.84	3	-0.4936	152	167
(K)GAAEREFSGLGNcLVK(I)	95%	4.594034	0.32596523	1,706.84	2	1.802	152	167
(K)GAAEREFSGLGNcLVK(I)	95%	4.5628014	0.34021795	1,706.84	2	1.373	152	167
(K)GAAEREFSGLGNcLVK(I)	95%	4.1736765	0.3725511	1,706.84	2	1.73	152	167
(K)GAAEREFSGLGNcLVK(I)	95%	4.662947	0.32619226	1,706.84	2	0.6579	152	167
(K)GAAEREFSGLGNcLVK(I)	95%	4.103069	0.3676039	1,706.84	2	0.3005	152	167
(K)GAAEREFSGLGNcLVK(I)	95%	4.4050136	0.37205523	1,706.84	2	1.659	152	167
(K)GAAEREFSGLGNcLVK(I)	95%	3.2923512	0.28647918	1,706.84	3	0.2569	152	167
(K)GAAEREFSGLGNcLVK(I)	95%	3.3524122	0.34767473	1,706.84	3	-0.2792	152	167
(K)GAAEREFSGLGNcLVK(I)	95%	3.6093342	0.40454334	1,706.84	3	0.3641	152	167
(K)GAAEREFSGLGNcLVK(I)	95%	4.267701	0.37927717	1,706.84	3	-0.4936	152	167
(K)GAAEREFSGLGNcLVK(I)	95%	4.9932413	0.3490622	1,706.84	3	1.329	152	167
(K)GAAEREFSGLGNcLVK(I)	95%	3.8584995	0.41060683	1,706.84	3	1.115	152	167
(K)GAAEREFSGLGNcLVK(I)	95%	3.5681164	0.43363008	1,706.84	3	-0.6008	152	167
(K)GAAEREFSGLGNcLVK(I)	95%	3.630801	0.4352496	1,706.84	3	-0.1719	152	167
(K)GAAEREFSGLGNcLVK(I)	95%	3.361742	0.45744517	1,706.84	3	0.2569	152	167
(K)GAAEREFSGLGNcLVK(I)	95%	3.8154376	0.50554705	1,706.86	3	9.156	152	167
(K)GAAEREFSGLGNcLVK(I)	95%	4.1165347	0.4938088	1,706.84	3	0.3641	152	167
(K)GAAEREFSGLGNcLVK(I)	95%	4.2268305	0.51835835	1,706.84	3	0.04248	152	167
(K)GALSNVFR(G)	95%	2.1093903	0.13997164	862.4672	1	1.322	275	282
(K)GALSNVFR(G)	95%	2.0947425	0.11859368	862.4669	1	0.9683	275	282
(K)GALSNVFR(G)	95%	2.733253	0.14200634	862.4657	2	-0.3903	275	282
(K)GALSNVFR(G)	95%	2.1409523	0.1356731	862.4663	1	0.2615	275	282
(K)GALSNVFR(G)	95%	2.7649665	0.16401793	862.4673	2	1.448	275	282
(K)GALSNVFR(G)	95%	2.1520994	0.17565547	862.4671	1	1.18	275	282
(K)GALSNVFR(G)	95%	1.7787877	0.25916785	862.4662	1	0.1908	275	282
(K)GALSNVFR(G)	95%	2.0535228	0.19064933	862.4664	1	0.4028	275	282
(K)GALSNVFR(G)	95%	2.218067	0.20015265	862.4672	1	1.322	275	282

(K)GALSNVFR(G)	95%	2.2280908	0.20371583	862.467	1	1.11	275	282
(K)GALSNVFR(G)	95%	2.425722	0.21972187	862.4666	2	0.5993	275	282
(K)GALSNVFR(G)	95%	3.1151867	0.2209658	862.4576	2	-9.862	275	282
(K)GALSNVFR(G)	95%	2.7255828	0.21644837	862.4653	2	-0.8851	275	282
(K)GALSNVFR(G)	95%	2.4948916	0.19512574	862.4669	1	0.9683	275	282
(K)GALSNVFR(G)	95%	2.757164	0.21872035	862.4674	2	1.518	275	282
(K)GALSNVFR(G)	95%	2.2557042	0.23844631	862.4661	1	0.04942	275	282
(K)GALSNVFR(G)	95%	2.8252585	0.19372973	862.4665	2	0.458	275	282
(K)GALSNVFR(G)	95%	3.061198	0.19322766	862.4669	2	0.9528	275	282
(K)GALSNVFR(G)	95%	3.2624168	0.21325316	862.4646	2	-1.663	275	282
(K)GALSNVFR(G)	95%	3.1136992	0.19409928	862.4666	2	0.67	275	282
(K)GALSNVFR(G)	95%	3.2151332	0.18275958	862.4666	2	0.67	275	282
(K)GALSNVFR(G)	95%	3.0585017	0.22709861	862.4668	2	0.8821	275	282
(K)GALSNVFR(G)	95%	3.2718217	0.20998053	862.4671	2	1.236	275	282
(K)GALSNVFR(G)	95%	3.0068066	0.23887621	862.4662	2	0.1752	275	282
(K)GALSNVFR(G)	95%	3.2513294	0.23256597	862.4673	2	1.377	275	282
(K)GALSNVFR(G)	95%	3.1556017	0.2292613	862.4668	2	0.8114	275	282
(K)GALSNVFR(G)	95%	3.0569818	0.23194608	862.4661	2	0.03385	275	282
(K)GALSNVFR(G)	95%	3.2641237	0.22653866	862.4655	2	-0.673	275	282
(K)GALSNVFR(G)	95%	3.0313997	0.2607574	862.4668	2	0.8114	275	282
(K)GALSNVFR(G)	95%	3.2454224	0.2512568	862.4674	2	1.589	275	282
(K)GALSNVFR(G)	95%	3.107881	0.24505328	862.466	2	-0.1075	275	282
(K)GALSNVFR(G)	95%	3.2728086	0.25559923	862.4675	2	1.66	275	282
(K)GALSNVFR(G)	95%	3.2426593	0.24648887	862.4659	2	-0.2489	275	282
(K)GALSNVFR(G)	95%	3.2792556	0.25424832	862.466	2	-0.03684	275	282
(K)GALSNVFR(G)	95%	3.2667885	0.2598676	862.466	2	-0.1075	275	282
(K)GDMMYK(G)	95%	1.5126622	0.44148973	743.2978	1	-0.7958	249	254
(K)GDMMYK(G)	95%	1.484158	0.5020471	743.2978	1	-0.7138	249	254
(K)GDMmYK(G)	95%	1.7284627	0.40603203	759.2943	2	1.396	249	254
(K)GDmMYK(G)	95%	2.3176954	0.37457898	759.2937	2	0.5931	249	254
(K)GDMmYK(G)	95%	1.7853979	0.5106333	759.2937	2	0.5128	249	254
(K)GDmMYK(G)	95%	2.1041937	0.47269508	759.294	2	0.9945	249	254

(K)GDmmYKGTLDcWAK(I)	95%	3.9733982	0.6197515	1,706.71	3	-0.2699	249	262
(K)GIIDcFVR(I)	95%	2.1546512	0.14961213	978.4969	1	1.061	57	64
(K)GIIDcFVR(I)	95%	1.8638796	0.2516322	978.4971	1	1.31	57	64
(K)GIIDcFVR(I)	95%	2.2514117	0.26940984	978.4972	2	1.421	57	64
(K)GIIDcFVR(I)	95%	1.6974087	0.3068577	978.4962	1	0.3754	57	64
(K)GIIDcFVR(I)	95%	1.727987	0.3215536	978.4966	1	0.7493	57	64
(K)GIIDcFVR(I)	95%	2.4785297	0.28077647	978.4968	2	0.9848	57	64
(K)GIIDcFVR(I)	95%	2.3074877	0.30378306	978.496	2	0.1747	57	64
(K)GIIDcFVR(I)	95%	2.5028763	0.33070144	978.4963	2	0.4863	57	64
(K)GIIDcFVR(I)	95%	2.5119596	0.35313565	978.4962	2	0.3617	57	64
(K)GIIDcFVR(I)	95%	2.4858432	0.37027666	978.4957	2	-0.1368	57	64
(K)GIIDcFVR(I)	95%	2.5201297	0.3841698	978.4963	2	0.4863	57	64
(K)GIIDcFVR(I)	95%	2.5692525	0.38164243	978.4963	2	0.424	57	64
(K)GIIDcFVR(I)	95%	2.6291032	0.37255466	978.4963	2	0.4863	57	64
(K)GIIDcFVR(I)	95%	2.6802676	0.3742613	978.4967	2	0.9225	57	64
(K)GIIDcFVR(I)	95%	2.6806183	0.38289022	978.4968	2	0.9848	57	64
(K)GIIDcFVRIPK(E)	95%	2.8032215	0.2966433	1,316.73	2	-0.1689	57	67
(K)GIIDcFVRIPK(E)	95%	2.4280086	0.36248276	1,316.73	2	-0.1689	57	67
(R)GTGGALVLVFYDELK(V)	95%	2.6325128	0.48991275	1,580.85	2	2.572	283	297
(K)GTLDcWAK(I)	95%	1.7364905	0.18059073	949.4335	1	0.6151	255	262
(K)GTLDcWAK(I)	95%	2.0755312	0.11373073	949.4331	1	0.1656	255	262
(K)GTLDcWAK(I)	95%	2.2286165	0.3046208	949.4303	2	-2.738	255	262
(K)GTLDcWAK(I)	95%	1.9990743	0.23527741	949.4323	1	-0.6693	255	262
(K)GTLDcWAK(I)	95%	2.154222	0.20821857	949.4324	1	-0.5408	255	262
(K)GTLDcWAK(I)	95%	1.9888573	0.36078155	949.4309	2	-2.096	255	262
(K)GTLDcWAK(I)	95%	2.134292	0.22964199	949.4327	1	-0.2198	255	262
(K)GTLDcWAK(I)	95%	2.1461556	0.20538472	949.4332	1	0.294	255	262
(K)GTLDcWAK(I)	95%	2.3855078	0.24094924	949.4328	1	-0.09132	255	262
(K)GTLDcWAK(I)	95%	2.0112195	0.4465137	949.4304	2	-2.674	255	262
(K)GTLDcWAK(I)	95%	2.277892	0.39194986	949.4315	2	-1.518	255	262
(K)GTLDcWAK(I)	95%	2.3227966	0.426429	949.4317	2	-1.261	255	262
(K)GTLDcWAK(I)	95%	2.1938019	0.47174695	949.4311	2	-1.904	255	262

(K)GTLDcWAK(I)	95%	2.0853653	0.48018357	949.4316	2	-1.454	255	262
(K)GTLDcWAK(I)	95%	2.1751792	0.4982586	949.4309	2	-2.096	255	262
(K)GTLDcWAK(I)	95%	2.229626	0.48887327	949.4313	2	-1.711	255	262
(K)GTLDcWAK(I)	95%	2.2443306	0.5112629	949.431	2	-2.032	255	262
(K)GTLDcWAK(I)	95%	2.1879344	0.51291114	949.4315	2	-1.518	255	262
(K)GTLDcWAK(I)	95%	2.1451464	0.51163846	949.4325	2	-0.4908	255	262
(K)GTLDcWAK(I)	95%	2.311002	0.5423342	949.4316	2	-1.454	255	262
(K)GTLDcWAK(I)	95%	2.4497602	0.52807117	949.4317	2	-1.261	255	262
(K)GTLDcWAK(I)	95%	2.2401235	0.53336793	949.4328	2	-0.1055	255	262
(K)GTLDcWAK(I)	95%	2.6432385	0.5401359	949.4305	2	-2.61	255	262
(K)GTLDcWAK(I)	95%	2.3842313	0.56283015	949.4323	2	-0.6834	255	262
(K)IFKSDGLTGLYR(G)	95%	2.8993323	0.37424907	1,368.74	2	1.397	168	179
(K)IFKSDGLTGLYR(G)	95%	3.2701888	0.51624084	1,368.74	2	3.268	168	179
(K)IFKSDGLTGLYR(G)	95%	3.2909892	0.5889296	1,368.74	3	0.3176	168	179
(K)IFKSDGLTGLYR(G)	95%	3.7007022	0.6348265	1,368.74	3	0.5181	168	179
(R)IPKEQGVLSFWR(G)	95%	3.5445368	0.07845205	1,458.80	2	0.4782	65	76
(R)IPKEQGVLSFWR(G)	95%	2.4234061	0.1574323	1,458.80	3	0.6989	65	76
(R)IPKEQGVLSFWR(G)	95%	4.0057025	0.08240045	1,458.80	2	0.1437	65	76
(R)IPKEQGVLSFWR(G)	95%	3.1981993	0.18422621	1,458.80	3	0.5108	65	76
(R)IPKEQGVLSFWR(G)	95%	3.418151	0.17774004	1,458.80	3	0.1972	65	76
(K)IYKSEGGGAFFK(G)	95%	1.7680788	0.32071903	1,302.66	3	-1.263	263	274
(K)IYKSEGGGAFFK(G)	95%	3.4623637	0.35458684	1,302.66	2	-1.487	263	274
(K)IYKSEGGGAFFK(G)	95%	2.2949436	0.538915	1,302.66	3	-2.246	263	274
(K)IYKSEGGGAFFK(G)	95%	3.1496255	0.46863353	1,302.67	3	9.622	263	274
(K)KQADPLSFLK(D)	95%	2.3935509	0.3080361	1,145.64	2	-0.1439	5	14
(K)KQADPLSFLK(D)	95%	2.6585102	0.37022975	1,145.65	2	0.6013	5	14
(K)KQADPLSFLK(D)	95%	2.9007037	0.3838272	1,145.64	2	-0.03745	5	14
(K)KQADPLSFLK(D)	95%	2.9948764	0.37955305	1,145.65	3	1.415	5	14
(K)KQADPLSFLK(D)	95%	2.8886328	0.47599858	1,145.65	3	1.175	5	14
(K)KQADPLSFLK(D)	95%	2.982048	0.49558163	1,145.65	3	1.494	5	14
(K)KQADPLSFLKDFmAGGISAAVSK(T)	95%	2.1164587	0.20274751	2,396.24	4	0.9653	5	27
(K)KQADPLSFLKDFmAGGISAAVSK(T)	95%	2.5371196	0.40829968	2,396.25	3	2.015	5	27

(K)KTQFWR(W)	95%	2.0874546	0.23042022	864.461	2	0.4198	110	115
(R)LAADTGKGAAER(E)	95%	1.8207656	0.24444056	1,158.60	3	0.8491	145	156
(R)LAADTGKGAAER(E)	95%	2.4473157	0.20296234	1,158.60	3	-0.6509	145	156
(R)LAADTGKGAAER(E)	95%	2.3398294	0.19744463	1,158.60	3	0.3754	145	156
(R)LAADTGKGAAER(E)	95%	3.1931424	0.34268218	1,158.60	2	-1.903	145	156
(R)LAADTGKGAAER(E)	95%	2.6201637	0.41179547	1,158.60	2	-0.2183	145	156
(R)LAADTGKGAAER(E)	95%	3.2196705	0.41602543	1,158.60	2	-2.639	145	156
(R)LAADTGKGAAER(E)	95%	3.958531	0.41223434	1,158.60	2	-1.903	145	156
(R)LAADTGKGAAER(E)	95%	3.5869803	0.46878248	1,158.60	2	-2.218	145	156
(R)LAADTGKGAAER(E)	95%	4.1655025	0.44191933	1,158.60	2	-1.587	145	156
(R)LAADTGKGAAER(E)	95%	3.9827065	0.45847896	1,158.60	2	-1.06	145	156
(R)LAADTGKGAAEREFSGLGNcLVK(I)	95%	2.5863118	0.30835155	2,363.19	3	1.006	145	167
(R)LAADTGKGAAEREFSGLGNcLVK(I)	95%	3.319415	0.40470487	2,363.19	4	-0.006188	145	167
(K)LLLQVQHVSK(Q)	95%	1.4754753	0.271618	1,163.70	3	1.345	38	47
(K)LLLQVQHVSK(Q)	95%	2.7368836	0.24348676	1,163.70	2	-2.128	38	47
(K)LLLQVQHVSK(Q)	95%	2.6868424	0.24933648	1,163.70	2	-1.604	38	47
(K)LLLQVQHVSK(Q)	95%	2.1053	0.22349757	1,163.70	3	-0.5416	38	47
(K)LLLQVQHVSK(Q)	95%	2.8045108	0.22479695	1,163.70	2	-0.8706	38	47
(K)LLLQVQHVSK(Q)	95%	1.9638305	0.35679767	1,163.70	1	-0.8067	38	47
(K)LLLQVQHVSK(Q)	95%	2.3432963	0.2738962	1,163.70	1	1.08	38	47
(K)LLLQVQHVSK(Q)	95%	3.0942981	0.2762408	1,163.70	2	-1.709	38	47
(K)LLLQVQHVSK(Q)	95%	2.3515673	0.4772153	1,163.70	1	-1.645	38	47
(K)LLLQVQHVSK(Q)	95%	1.4810398	0.3255623	1,163.70	3	0.4803	38	47
(K)LLLQVQHVSK(Q)	95%	3.2235718	0.2846502	1,163.70	2	-2.338	38	47
(K)LLLQVQHVSK(Q)	95%	3.0686843	0.32609633	1,163.70	2	-2.548	38	47
(K)LLLQVQHVSK(Q)	95%	3.0671775	0.324124	1,163.70	2	-1.919	38	47
(K)LLLQVQHVSK(Q)	95%	2.8915157	0.3310712	1,163.70	2	-0.7658	38	47
(K)LLLQVQHVSK(Q)	95%	1.6190548	0.35731462	1,163.70	3	-0.1485	38	47
(K)LLLQVQHVSK(Q)	95%	2.9756415	0.37010607	1,163.70	2	-1.395	38	47
(K)LLLQVQHVSK(Q)	95%	3.0522633	0.37548876	1,163.70	2	-1.709	38	47
(K)LLLQVQHVSK(Q)	95%	2.8915844	0.4128757	1,163.70	2	-1.395	38	47
(K)LLLQVQHVSK(Q)	95%	3.0115836	0.41509122	1,163.70	2	-1.919	38	47

(K)LLLQVQHVSK(Q)	95%	3.535905	0.36458668	1,163.70	2	-1.709	38	47
(K)LLLQVQHVSK(Q)	95%	1.4231187	0.43414038	1,163.70	3	0.3231	38	47
(K)LLLQVQHVSK(Q)	95%	3.347499	0.37036115	1,163.70	2	-0.4514	38	47
(K)LLLQVQHVSK(Q)	95%	2.9226754	0.49585915	1,163.70	2	-2.024	38	47
(K)LLLQVQHVSK(Q)	95%	3.594784	0.4226729	1,163.70	2	-1.185	38	47
(K)LLLQVQHVSK(Q)	95%	3.5751622	0.45092395	1,163.70	2	-1.604	38	47
(K)LLLQVQHVSK(Q)	95%	3.815086	0.43014836	1,163.70	2	-1.395	38	47
(K)LLLQVQHVSK(Q)	95%	3.1745296	0.4866386	1,163.70	2	-0.3466	38	47
(K)LLLQVQHVSK(Q)	95%	3.328596	0.51398623	1,163.70	2	-1.395	38	47
(R)MMMQSGR(A)	95%	2.0869684	0.33135536	839.3455	2	0.2663	240	246
(R)mMMQSGRAKGDMMYK(G)	95%	1.8477952	0.3092696	1,779.75	3	-7.125	240	254
(K)QADPLSFLK(D)	95%	2.093247	0.18537295	1,017.55	1	0.9586	6	14
(K)QADPLSFLK(D)	95%	2.230648	0.25168014	1,017.55	2	-0.4928	6	14
(K)QADPLSFLK(D)	95%	2.0276616	0.29245678	1,017.55	2	-0.7325	6	14
(K)QADPLSFLK(D)	95%	2.4565835	0.2135228	1,017.55	2	0.2263	6	14
(K)QADPLSFLK(D)	95%	1.8583659	0.4324873	1,017.54	2	-8.283	6	14
(K)QADPLSFLK(D)	95%	2.2112777	0.27919084	1,017.55	2	1.185	6	14
(K)QADPLSFLK(D)	95%	2.3817923	0.30870324	1,017.55	2	0.7057	6	14
(K)QADPLSFLK(D)	95%	1.9469761	0.3884806	1,017.55	2	0.466	6	14
(K)QADPLSFLKDFmAGGISAAVSK(T)	95%	3.3865712	0.5217803	2,268.15	3	1.039	6	27
(K)QIAVDKQYK(G)	95%	2.1142523	0.23857214	1,091.60	2	0.0751	48	56
(K)QIAVDKQYK(G)	95%	1.9657215	0.41103673	1,091.60	2	-1.824	48	56
(K)QIAVDKQYK(G)	95%	2.1590104	0.38945273	1,091.60	2	-0.8187	48	56
(K)QIFmSGVDK(K)	95%	1.6199824	0.36894557	1,039.50	2	-0.03336	101	109
(K)QIFmSGVDK(K)	95%	1.9372901	0.31383723	1,039.50	1	0.03822	101	109
(K)QIFmSGVDK(K)	95%	1.657301	0.44113243	1,039.50	2	-0.5026	101	109
(K)QIFmSGVDK(K)	95%	2.2795851	0.33424854	1,039.50	2	-1.089	101	109
(K)QIFmSGVDKK(T)	95%	2.0199926	0.312402	1,167.59	2	-0.9949	101	110
(K)QIFmSGVDKK(T)	95%	2.2035015	0.16736995	1,167.60	1	0.008964	101	110
(K)QIFmSGVDKK(T)	95%	2.454099	0.2192597	1,167.60	2	0.1541	101	110
(K)QIFmSGVDKK(T)	95%	2.0346835	0.2621284	1,167.60	1	0.5313	101	110
(K)QIFMSGVDKK(T)	95%	1.5806884	0.34133703	1,151.60	3	1.425	101	110

(K)SDGLTGLYR(G)	95%	1.8944916	0.32653877	980.4945	1	1.655	171	179
(K)SDGLTGLYR(G)	95%	1.9732593	0.36168957	980.4945	1	1.655	171	179
(K)SDGLTGLYR(G)	95%	2.0716112	0.4539919	980.4951	1	2.276	171	179
(K)SDGLTGLYR(G)	95%	1.8881284	0.3371118	980.4932	1	0.2865	171	179
(K)SDGLTGLYR(G)	95%	1.8924822	0.37301728	980.4938	1	0.9706	171	179
(K)SDGLTGLYR(G)	95%	1.9931561	0.4683837	980.4939	1	1.033	171	179
(K)SDGLTGLYR(G)	95%	2.2332523	0.45096633	980.4941	1	1.219	171	179
(K)SDGLTGLYR(G)	95%	2.8463807	0.31259373	980.4942	2	1.33	171	179
(K)SDGLTGLYR(G)	95%	3.1078343	0.3250659	980.4942	2	1.392	171	179
(K)SDGLTGLYR(G)	95%	3.1197035	0.33238906	980.494	2	1.143	171	179
(K)SDGLTGLYR(G)	95%	3.0121412	0.35681894	980.4938	2	0.9569	171	179
(K)SDGLTGLYR(G)	95%	2.8992865	0.38255253	980.4939	2	1.019	171	179
(K)SDGLTGLYR(G)	95%	2.9916782	0.3812662	980.4934	2	0.5838	171	179
(K)SDGLTGLYR(G)	95%	3.0679457	0.36475766	980.493	2	0.08629	171	179
(K)SDGLTGLYR(G)	95%	2.9264944	0.3882519	980.4929	2	0.02411	171	179
(K)SDGLTGLYR(G)	95%	3.2026637	0.39623982	980.4936	2	0.7081	171	179
(K)SDGLTGLYR(G)	95%	3.2684362	0.40333486	980.4934	2	0.5216	171	179
(K)SDGLTGLYR(G)	95%	3.7459607	0.3648864	980.4929	2	0.02411	171	179
(K)SDGLTGLYR(G)	95%	3.1860924	0.4489598	980.4939	2	1.019	171	179
(K)SDGLTGLYR(G)	95%	3.1725945	0.45934334	980.4925	2	-0.4112	171	179
(K)SDGLTGLYR(G)	95%	3.0595362	0.46616444	980.4926	2	-0.2246	171	179
(K)SDGLTGLYR(G)	95%	3.5653825	0.41325665	980.4932	2	0.335	171	179
(K)SDGLTGLYR(G)	95%	3.1185906	0.4897259	980.4921	2	-0.7843	171	179
(K)SDGLTGLYR(G)	95%	3.1708715	0.4812774	980.4934	2	0.5216	171	179
(K)SEGGGAFFK(G)	95%	2.5530674	0.16366597	898.419	2	0.4559	266	274
(K)SEGGGAFFK(G)	95%	2.0059695	0.17326008	898.4194	1	0.8781	266	274
(K)SEGGGAFFK(G)	95%	2.1073635	0.17893317	898.4194	1	0.8781	266	274
(K)SEGGGAFFK(G)	95%	2.148703	0.17720199	898.4189	1	0.3352	266	274
(K)SEGGGAFFK(G)	95%	2.1039927	0.20575237	898.4192	1	0.6745	266	274
(K)SEGGGAFFK(G)	95%	2.1327548	0.20212182	898.4186	1	0.06373	266	274
(K)SEGGGAFFK(G)	95%	2.18095	0.1952799	898.4187	1	0.1995	266	274
(K)SEGGGAFFK(G)	95%	2.1857061	0.19979474	898.4193	1	0.8102	266	274

(K)SEGGGAFFK(G)	95%	2.1584034	0.21565874	898.4194	1	0.9459	266	274
(K)SEGGGAFFK(G)	95%	2.3015492	0.18217489	898.419	1	0.4709	266	274
(K)SEGGGAFFK(G)	95%	2.2408853	0.20282476	898.4191	1	0.6066	266	274
(K)SEGGGAFFK(G)	95%	2.3124275	0.20142739	898.4188	1	0.2673	266	274
(K)SEGGGAFFK(G)	95%	2.3130224	0.20516488	898.4187	1	0.1316	266	274
(K)SEGGGAFFK(G)	95%	2.7564723	0.19943765	898.419	2	0.4559	266	274
(K)SEGGGAFFK(G)	95%	2.506864	0.27495208	898.4191	2	0.5917	266	274
(K)SEGGGAFFK(G)	95%	2.8956842	0.25591558	898.4176	2	-1.037	266	274
(K)SEGGGAFFK(G)	95%	2.844472	0.2597162	898.4193	2	0.7952	266	274
(K)SEGGGAFFK(G)	95%	2.8614779	0.253084	898.4186	2	-0.01908	266	274
(K)SEGGGAFFK(G)	95%	2.9150205	0.30493572	898.4173	2	-1.444	266	274
(K)SEGGGAFFK(G)	95%	2.7983398	0.3087139	898.4179	2	-0.7655	266	274
(K)SEGGGAFFK(G)	95%	2.984172	0.27617595	898.4188	2	0.2524	266	274
(K)SEGGGAFFK(G)	95%	3.1342516	0.29812703	898.4186	2	0.04878	266	274
(K)SEGGGAFFK(G)	95%	3.2637327	0.3048895	898.4184	2	-0.2227	266	274
(K)SEGGGAFFK(G)	95%	3.3751757	0.3281688	898.4177	2	-0.9691	266	274
(K)SEGGGAFFK(G)	95%	3.3854392	0.32756576	898.4188	2	0.2524	266	274
(K)SEGGGAFFK(G)	95%	3.191428	0.34685618	898.4184	2	-0.1548	266	274
(K)SEGGGAFFK(G)	95%	3.341646	0.3460699	898.4187	2	0.1166	266	274
(K)TAVAPIER(V)	95%	1.6638585	0.2165509	855.4822	1	0.8114	28	35
(K)TAVAPIER(V)	95%	1.9591287	0.37335697	855.49	2	9.917	28	35
(K)TAVAPIER(V)	95%	1.9311104	0.26721448	855.4817	2	0.2256	28	35
(K)TAVAPIER(V)	95%	2.0038016	0.20124407	855.4822	1	0.8114	28	35
(K)TAVAPIER(V)	95%	1.7806921	0.2531279	855.4821	1	0.7401	28	35
(K)TAVAPIER(V)	95%	1.9824091	0.27324882	855.4813	2	-0.202	28	35
(K)TAVAPIER(V)	95%	1.988988	0.28020105	855.4815	2	0.01177	28	35
(K)TAVAPIER(V)	95%	1.8907385	0.23421769	855.482	1	0.5976	28	35
(K)TAVAPIER(V)	95%	2.2176168	0.18933325	855.4822	1	0.8114	28	35
(K)TAVAPIER(V)	95%	2.2425036	0.28180626	855.4807	2	-0.8434	28	35
(K)TAVAPIER(V)	95%	1.9460136	0.25704804	855.4817	1	0.3125	28	35
(K)TAVAPIER(V)	95%	2.231076	0.2678379	855.4814	1	-0.1151	28	35
(K)TAVAPIER(V)	95%	2.2163718	0.24841684	855.4814	1	-0.04379	28	35

(K)TAVAPIER(V)	95%	2.1727836	0.30940548	855.4811	2	-0.4158	28	35
(K)TAVAPIER(V)	95%	2.2319655	0.2827716	855.4823	1	0.9539	28	35
(K)TAVAPIER(V)	95%	2.2720864	0.3082388	855.4814	1	-0.1151	28	35
(K)TAVAPIER(V)	95%	2.2161617	0.29722184	855.4816	1	0.17	28	35
(K)TAVAPIER(V)	95%	1.9887035	0.4500097	855.4898	2	9.703	28	35
(K)TAVAPIER(V)	95%	2.1957834	0.31203052	855.4812	2	-0.2733	28	35
(K)TAVAPIER(V)	95%	2.2527611	0.30855027	855.4813	2	-0.202	28	35
(K)TAVAPIER(V)	95%	2.2533267	0.33379123	855.4819	1	0.455	28	35
(K)TAVAPIER(V)	95%	2.3892305	0.34529173	855.481	2	-0.5583	28	35
(K)TAVAPIERVK(L)	95%	2.63552	0.34184346	1,082.64	2	-0.5621	28	37
(K)TAVAPIERVK(L)	95%	1.815985	0.37115753	1,082.65	3	1.65	28	37
(R)VKLLLQVQHVSK(Q)	95%	1.7999562	0.25207207	1,390.87	3	0.7902	36	47
(R)VKLLLQVQHVSK(Q)	95%	3.4553676	0.48220983	1,390.87	2	0.2736	36	47
(R)VKLLLQVQHVSK(Q)	95%	4.0575843	0.45467943	1,390.87	2	0.01054	36	47
(R)VKLLLQVQHVSK(Q)	95%	2.4454072	0.6047882	1,390.86	3	-1.644	36	47
(R)VKLLLQVQHVSK(Q)	95%	3.1039727	0.5548949	1,390.87	3	0.0008825	36	47
(R)VKLLLQVQHVSK(Q)	95%	2.6154797	0.59583706	1,390.86	3	-0.9858	36	47
(R)VKLLLQVQHVSKQIAVDK(Q)	95%	1.0935079	0.3645995	2,045.22	4	-7.682	36	53
(R)YFPTQALNFAFK(D)	95%	2.262077	0.43250582	1,445.74	1	1.64	85	96
(R)YFPTQALNFAFK(D)	95%	3.0393176	0.42814085	1,445.74	1	2.231	85	96
(R)YFPTQALNFAFK(D)	95%	2.156211	0.44674215	1,445.74	2	1.167	85	96
(R)YFPTQALNFAFK(D)	95%	2.7430909	0.47866663	1,445.73	2	-0.7741	85	96
(R)YFPTQALNFAFK(D)	95%	2.8202622	0.48975176	1,445.74	2	0.7447	85	96
(R)YFPTQALNFAFK(D)	95%	2.9271374	0.48068848	1,445.73	2	-0.09907	85	96
(R)YFPTQALNFAFK(D)	95%	2.6286888	0.5371303	1,445.73	2	-0.521	85	96
(R)YFPTQALNFAFK(D)	95%	2.5356147	0.5958509	1,445.74	2	1.42	85	96
(R)YFPTQALNFAFK(D)	95%	3.2520263	0.53660214	1,445.73	2	-0.521	85	96
(R)YFPTQALNFAFK(D)	95%	3.2859042	0.54680014	1,445.74	2	0.7447	85	96
(R)YFPTQALNFAFK(D)	95%	3.6676724	0.5188281	1,445.74	2	0.9134	85	96
(R)YFPTQALNFAFK(D)	95%	3.4329646	0.56301296	1,445.73	2	0.1541	85	96
(R)YFPTQALNFAFK(D)	95%	3.357703	0.57379436	1,445.73	2	0.1541	85	96
(R)YFPTQALNFAFK(D)	95%	3.7794917	0.5778266	1,445.73	2	0.06968	85	96

(R)YFPTQALNFAFK(D)	95%	3.5880592	0.59680265	1,445.73	2	0.06968	85	96
(R)YFPTQALNFAFK(D)	95%	3.6605148	0.58982116	1,445.74	2	0.6603	85	96
(R)YFPTQALNFAFK(D)	95%	3.5151956	0.6110178	1,445.73	2	-0.0147	85	96
(R)YFPTQALNFAFK(D)	95%	3.7105825	0.61407876	1,445.74	2	2.348	85	96

c denotes Cys alkylation by iodoacetamide during trypsin digestion
m denotes Met oxidation
* As determined by Peptide Profit Algorithm embedded within Scaffold v 3.6.1

ArAAC peptides identified by triplicate LC-LC-MS/MS on the LTQ from *Artemia franciscana* purified mitochondria in RIPA as described in Materials and Methods

**Sequence**	**Prob***	**SEQUEST XCorr**	**SEQUEST deltaCn**	**X! Tandem**	**Actual Mass**	**Charge**	**Delta AMU**	**Start**	**Stop**
(R)AAYFGFYDTVR(G)	95%	2.685237	0.4411779		1,308.66	2	0.04748	193	203
(R)AAYFGFYDTVR(G)	95%	2.648569	0.4726893		1,308.58	2	-0.02979	193	203
(R)AAYFGFYDTVR(G)	95%	2.8610587	0.46747932		1,309.20	2	0.5841	193	203
(R)AAYFGFYDTVR(G)	95%	2.8352282	0.48776382		1,309.43	2	0.8115	193	203
(R)AAYFGFYDTVR(G)	95%	2.7590458	0.5124591		1,309.50	2	0.8905	193	203
(R)AAYFGFYDTVR(G)	95%	3.0130107	0.5448527		1,310.35	2	1.738	193	203
(R)AAYFGFYDTVR(G)	95%	3.026287	0.5151303		1,309.18	2	0.5669	193	203
(R)AAYFGFYDTVR(G)	95%	2.8009727	0.5377877		1,309.36	2	0.748	193	203
(R)AAYFGFYDTVR(G)	95%	2.9766085	0.57261056	2.3767507	1,310.31	2	1.693	193	203
(R)AAYFGFYDTVR(G)	95%	3.2005208	0.5482896	1.9208188	1,309.14	2	0.5256	193	203
(K)DFMAGGISAAVSK(T)	95%	3.0665588	0.42747465		1,253.05	2	0.4336	15	27
(K)DFMAGGISAAVSK(T)	95%	3.460446	0.4538525		1,252.48	2	-0.1307	15	27
(K)DFMAGGISAAVSK(T)	95%	3.878824	0.48875752	3.9208188	1,253.41	2	0.7985	15	27
(K)DFmAGGISAAVSK(T)	95%	4.094514	0.5444428	6.5850267	1,270.42	2	1.814	15	27
(K)DFMAGGISAAVSK(T)	95%	4.087361	0.5094583	4.49485	1,253.28	2	0.6691	15	27
(K)DFMAGGISAAVSK(T)	95%	4.244789	0.50896347	5.853872	1,253.21	2	0.593	15	27
(K)DFmAGGISAAVSK(T)	95%	4.045934	0.5752531	4.5086384	1,268.96	2	0.3517	15	27

(K)DFmAGGISAAVSK(T)	95%	3.9557106	0.578401	4.537602	1,269.29	2	0.681	15	27
(K)DFmAGGISAAVSK(T)	95%	4.2690477	0.5873821	5.481486	1,269.40	2	0.7928	15	27
(R)EFSGLGNcLVK(I)	95%	2.6782773	0.3143434		1,224.44	2	1.838	157	167
(R)EFSGLGNcLVK(I)	95%	2.6059082	0.32313314		1,222.87	2	0.2644	157	167
(R)EFSGLGNcLVK(I)	95%	2.5606873	0.33335125		1,223.55	2	0.9526	157	167
(R)EFSGLGNcLVK(I)	95%	2.5287132	0.43562016		1,224.77	2	2.166	157	167
(R)EFSGLGNcLVK(I)	95%	2.578121	0.3864073		1,223.26	2	0.6613	157	167
(R)EFSGLGNcLVK(I)	95%	2.5671163	0.35663262	1.5850266	1,223.23	2	0.6241	157	167
(R)EFSGLGNcLVK(I)	95%	2.7588027	0.37443274		1,223.49	2	0.8905	157	167
(R)EFSGLGNcLVK(I)	95%	2.8093235	0.3415122	1.031517	1,223.65	2	1.049	157	167
(R)EFSGLGNcLVK(I)	95%	2.5766175	0.40706018	0.3665315	1,223.44	2	0.8387	157	167
(R)EFSGLGNcLVK(I)	95%	2.774996	0.38119036		1,223.32	2	0.7209	157	167
(R)EFSGLGNcLVK(I)	95%	2.7242005	0.3748197	1.19382	1,223.21	2	0.6095	157	167
(R)EFSGLGNcLVK(I)	95%	2.6361654	0.44102716	0.7695511	1,223.44	2	0.8399	157	167
(R)EFSGLGNcLVK(I)	95%	2.7669003	0.42998073		1,223.15	2	0.5502	157	167
(R)EFSGLGNcLVK(I)	95%	2.8202922	0.41967493	0.6382721	1,223.33	2	0.7237	157	167
(R)EFSGLGNcLVK(I)	95%	3.0817535	0.40304202		1,223.04	2	0.4397	157	167
(K)EQGVLSFWR(G)	93%	2.658475	0.096862555	0.0809219	1,122.57	2	2.007	68	76
(K)EQGVLSFWR(G)	95%	2.1738067	0.151346		1,120.78	1	0.213	68	76
(K)EQGVLSFWR(G)	95%	2.529674	0.11888237	0.4089354	1,121.32	2	0.7512	68	76
(K)EQGVLSFWR(G)	95%	2.5322783	0.1260045		1,121.37	2	0.801	68	76
(K)EQGVLSFWR(G)	95%	2.6923537	0.102029815	0.4685211	1,121.33	2	0.7646	68	76
(K)EQGVLSFWR(G)	95%	2.6106446	0.13410443		1,120.72	2	0.1564	68	76
(K)EQGVLSFWR(G)	95%	2.661075	0.10779724	0.5086383	1,121.21	2	0.6442	68	76
(K)EQGVLSFWR(G)	95%	2.6112947	0.22593291	-0.1139433	1,120.88	2	0.3085	68	76
(K)EQGVLSFWR(G)	95%	2.2872205	0.13702147	-0.2787536	1,121.05	1	0.4793	68	76
(K)GALSNVFR(G)	83%	1.8086149	0.20672633		862.5901	1	0.124	275	282
(K)GALSNVFR(G)	95%	2.6429932	0.10181974		863.255	2	0.7889	275	282

(K)GALSNVFR(G)	95%	2.81162	0.12392415		864.1367	2	1.671	275	282
(K)GALSNVFR(G)	95%	2.548284	0.12547417	0	863.0659	2	0.5998	275	282
(K)GALSNVFR(G)	95%	2.56936	0.13799196	0.3187588	863.1432	2	0.6771	275	282
(K)GALSNVFR(G)	95%	2.6523054	0.13574567	0.0087739	863.267	2	0.8009	275	282
(K)GALSNVFR(G)	95%	2.5608668	0.14994067		863.0999	2	0.6339	275	282
(K)GALSNVFR(G)	95%	2.6939197	0.15141977		862.5191	2	0.05301	275	282
(K)GALSNVFR(G)	95%	2.6045823	0.16231522	0.69897	862.6186	2	0.1526	275	282
(K)GALSNVFR(G)	95%	2.6235168	0.18645547	0	862.4581	2	-0.00796	275	282
(K)GALSNVFR(G)	95%	2.6228812	0.18703611		862.6007	2	0.1346	275	282
(K)GALSNVFR(G)	95%	2.7197933	0.15743707	-0.3802112	862.6671	2	0.201	275	282
(K)GALSNVFR(G)	95%	2.7317407	0.16573699		862.5787	2	0.1126	275	282
(K)GALSNVFR(G)	95%	2.842262	0.14799267	0.4436974	863.159	2	0.693	275	282
(K)GALSNVFR(G)	95%	2.6872866	0.17449296		863.0924	2	0.6263	275	282
(K)GALSNVFR(G)	95%	2.5414143	0.19645442	0.4089354	863.0448	2	0.5788	275	282
(K)GALSNVFR(G)	95%	2.848084	0.20970137		863.3968	2	0.9307	275	282
(K)GALSNVFR(G)	95%	2.9460216	0.20673741		863.0426	2	0.5766	275	282
(K)GIIDcFVR(I)	95%	2.532745	0.3545571	0.6777807	978.8095	2	0.3137	57	64
(K)GIIDcFVR(I)	95%	2.5152135	0.3762936	0.30103	978.6173	2	0.1214	57	64
(K)GIIDcFVR(I)	95%	2.7488642	0.36850032		980.2661	2	1.77	57	64
(K)GIIDcFVR(I)	95%	2.5333138	0.36670178		979.0503	2	0.5545	57	64
(K)GIIDcFVR(I)	95%	2.5057592	0.3894979	0.5228787	979.5418	2	1.046	57	64
(K)GIIDcFVR(I)	95%	2.525171	0.39049137		979.2109	2	0.715	57	64
(K)GIIDcFVR(I)	95%	2.5670257	0.3943214	0.2441251	979.2271	2	0.7313	57	64
(K)GIIDcFVR(I)	95%	2.5759797	0.41577283	-0.0791812	979.1234	2	0.6276	57	64
(K)GIIDcFVR(I)	95%	2.7746177	0.40109944		978.8969	2	0.4011	57	64
(R)GNLANVIR(Y)	95%	2.5511174	0.304253	1.1079054	856.3589	2	0.8662	77	84
(R)GTGGALVLVFYDELK (V)	95%	2.593949	0.38939688		1,582.72	2	1.878	283	297

(R)GTGGALVLVFYDELK (V)	95%	2.7102118	0.36137348		1,582.18	2	1.332	283	297
(R)GTGGALVLVFYDELK (V)	95%	3.330484	0.39333206		1,581.20	2	0.3553	283	297
(R)GTGGALVLVFYDELK (V)	95%	2.5367422	0.524334	0.5086383	1,582.82	2	1.978	283	297
(R)GTGGALVLVFYDELK (V)	95%	3.6636374	0.45014498		1,580.93	2	0.08866	283	297
(K)GTLDcWAK(I)	95%	2.5487409	0.37785608	0.2924298	950.1459	2	0.7129	255	262
(K)IFKSDGLTGLYR(G)	95%	3.422279	0.38107288		1,369.71	2	0.9714	168	179
(R)IPKEQGVLSFWR(G)	95%	3.6892684	0.09406028	0.0043648 01	1,460.11	3	1.31	65	76
(R)IPKEQGVLSFWR(G)	95%	3.558448	0.118006125	-0.30103	1,460.07	3	1.27	65	76
(R)IPKEQGVLSFWR(G)	95%	3.3576636	0.08260018	3.0604808	1,460.28	2	1.486	65	76
(R)IPKEQGVLSFWR(G)	95%	3.3806689	0.10023529	2.2006595	1,459.24	2	0.4423	65	76
(K)IYKSEGGGAFFK(G)	95%	3.2847567	0.2928096		1,303.44	2	0.7759	263	274
(K)KQADPLSFLK(D)	95%	2.5220578	0.1885909		1,146.44	2	0.7917	5	14
(K)KQADPLSFLK(D)	95%	2.5975099	0.18346627	0.4948500	1,146.50	2	0.8586	5	14
(K)KQADPLSFLK(D)	95%	2.5877845	0.2018341		1,146.15	2	0.5014	5	14
(K)KQADPLSFLK(D)	95%	2.5454721	0.24138416		1,146.28	2	0.6334	5	14
(K)KQADPLSFLK(D)	95%	2.563853	0.24489212		1,146.38	2	0.732	5	14
(K)KQADPLSFLK(D)	95%	2.8048806	0.27782482		1,146.32	2	0.6705	5	14
(K)KQADPLSFLK(D)	95%	2.8728914	0.29667163		1,146.11	2	0.4686	5	14
(K)KQADPLSFLK(D)	95%	2.588355	0.37909856		1,146.47	2	0.8256	5	14
(K)KQADPLSFLK(D)	95%	2.933542	0.34772336	0.7695511	1,146.31	2	0.663	5	14
(K)KQADPLSFLKDFmA GGISAAVSK(T)	95%	3.5408514	0.43353292		2,396.51	3	0.2665	5	27
(K)KQADPLSFLKDFmA GGISAAVSK(T)	95%	3.6680741	0.48746336		2,396.51	3	0.2723	5	27
(K)KQADPLSFLKDFmA	95%	4.704733	0.5356906		2,398.42	3	2.174	5	27

GGISAAVSK(T)									
(K)LLLQVQHVSK(Q)	95%	3.1366827	0.27438703		1,164.34	2	0.6345	38	47
(K)LLLQVQHVSK(Q)	95%	3.219312	0.30568317		1,163.84	2	0.1372	38	47
(K)LLLQVQHVSK(Q)	95%	3.125982	0.31548345	0.7695511	1,164.00	2	0.3017	38	47
(K)LLLQVQHVSK(Q)	95%	3.1803634	0.36482602		1,165.32	2	1.614	38	47
(K)LLLQVQHVSK(Q)	95%	3.3461647	0.31279135		1,164.46	2	0.7557	38	47
(K)LLLQVQHVSK(Q)	95%	3.1265345	0.35580513	1.79588	1,164.39	2	0.686	38	47
(K)LLLQVQHVSK(Q)	95%	3.272523	0.3437851	0.5850267	1,164.44	2	0.733	38	47
(K)LLLQVQHVSK(Q)	95%	3.2829	0.40624413		1,165.42	2	1.721	38	47
(K)LLLQVQHVSK(Q)	95%	3.1878014	0.38261396	0.5228787	1,164.26	2	0.5564	38	47
(K)LLLQVQHVSK(Q)	95%	3.2791066	0.37909675		1,164.60	2	0.8995	38	47
(K)LLLQVQHVSK(Q)	95%	3.2946937	0.38550127		1,164.78	2	1.077	38	47
(K)LLLQVQHVSK(Q)	95%	3.505244	0.37092602		1,163.85	2	0.1508	38	47
(K)LLLQVQHVSK(Q)	95%	3.164018	0.39230648		1,164.46	2	0.7548	38	47
(K)LLLQVQHVSK(Q)	95%	3.2480884	0.38667923		1,164.52	2	0.8189	38	47
(K)LLLQVQHVSK(Q)	95%	3.3231742	0.39570394		1,164.02	2	0.3145	38	47
(K)LLLQVQHVSK(Q)	95%	3.640755	0.36250198	0.6197887	1,164.02	2	0.3189	38	47
(K)LLLQVQHVSK(Q)	95%	3.4948707	0.36405468	0.7695511	1,164.41	2	0.7027	38	47
(K)LLLQVQHVSK(Q)	95%	3.2286525	0.40699512	0.5686362	1,164.66	2	0.9577	38	47
(K)LLLQVQHVSK(Q)	95%	3.4898293	0.37955716		1,164.56	2	0.8591	38	47
(K)LLLQVQHVSK(Q)	95%	3.5360453	0.38694605		1,163.97	2	0.2656	38	47
(K)LLLQVQHVSK(Q)	95%	3.5283	0.38855198	0.7212464	1,164.67	2	0.9641	38	47
(K)LLLQVQHVSK(Q)	95%	3.0591092	0.43856674		1,164.25	2	0.5512	38	47
(K)LLLQVQHVSK(Q)	95%	3.4435585	0.39266387		1,164.29	2	0.5866	38	47
(K)LLLQVQHVSK(Q)	95%	3.3141518	0.41241238		1,164.62	2	0.9134	38	47
(K)LLLQVQHVSK(Q)	95%	3.3819497	0.44041383		1,165.40	2	1.697	38	47
(K)LLLQVQHVSK(Q)	95%	3.3762107	0.4140155	0.6197887	1,164.17	2	0.4694	38	47
(K)LLLQVQHVSK(Q)	95%	3.6514509	0.4036014		1,163.74	2	0.03353	38	47

(K)LLLQVQHVSK(Q)	95%	3.518861	0.43572745		1,165.36	2	1.658	38	47
(K)LLLQVQHVSK(Q)	95%	3.561691	0.39501834	0.8860566	1,164.48	2	0.7755	38	47
(K)LLLQVQHVSK(Q)	95%	3.31907	0.42146474		1,164.36	2	0.6579	38	47
(K)LLLQVQHVSK(Q)	95%	3.5146668	0.40438372		1,164.45	2	0.7502	38	47
(K)LLLQVQHVSK(Q)	95%	3.4308236	0.41247585		1,164.42	2	0.7131	38	47
(K)LLLQVQHVSK(Q)	95%	3.6235392	0.42179525		1,163.76	2	0.05892	38	47
(K)LLLQVQHVSK(Q)	95%	3.3965235	0.43350142		1,164.10	2	0.3963	38	47
(K)LLLQVQHVSK(Q)	95%	3.2383254	0.44101298		1,164.36	2	0.6546	38	47
(K)LLLQVQHVSK(Q)	95%	3.5536065	0.41685903		1,164.47	2	0.7685	38	47
(K)LLLQVQHVSK(Q)	95%	3.5811365	0.41690752		1,164.51	2	0.8048	38	47
(K)LLLQVQHVSK(Q)	95%	3.3454645	0.441406		1,164.46	2	0.7595	38	47
(K)LLLQVQHVSK(Q)	95%	3.2827845	0.45592102		1,164.19	2	0.4881	38	47
(K)QADPLSFLKDFmAG GISAAVSK(T)	95%	2.6063857	0.42231137		2,269.40	2	1.254	6	27
(K)QADPLSFLKDFmAG GISAAVSK(T)	95%	3.0323544	0.37392104	1	2,268.38	2	0.2354	6	27
(K)QADPLSFLKDFmAG GISAAVSK(T)	95%	2.82057	0.50790966	1.251812	2,270.62	2	2.473	6	27
(K)QADPLSFLKDFmAG GISAAVSK(T)	95%	2.9177656	0.51765805		2,269.50	2	1.357	6	27
(K)QADPLSFLKDFmAG GISAAVSK(T)	95%	3.385476	0.5311633		2,269.87	2	1.728	6	27
(K)QADPLSFLKDFmAG GISAAVSK(T)	95%	3.3123834	0.56926006	1.236572	2,270.07	2	1.92	6	27
(K)QADPLSFLKDFmAG GISAAVSK(T)	95%	3.5857403	0.57544094	0.39794	2,269.03	2	0.8807	6	27
(K)QADPLSFLKDFmAG GISAAVSK(T)	95%	4.2112765	0.45611915		2,269.44	3	1.292	6	27
(K)QADPLSFLKDFmAG GISAAVSK(T)	95%	3.5066478	0.5066593		2,269.59	3	1.442	6	27
(K)QADPLSFLKDFmAG	95%	3.640904	0.52587146	1.69897	2,269.01	3	0.8597	6	27

GISAAVSK(T)									
(K)QADPLSFLKDFmAG GISAAVSK(T)	95%	3.5269868	0.53934526	0.9208187	2,268.89	3	0.7454	6	27
(K)QADPLSFLKDFmAG GISAAVSK(T)	95%	4.0143476	0.55547357		2,270.23	3	2.081	6	27
(K)QADPLSFLKDFmAG GISAAVSK(T)	95%	4.348121	0.56682104	0.3565473	2,270.37	3	2.225	6	27
(K)QADPLSFLKDFmAG GISAAVSK(T)	95%	4.198876	0.59479445		2,269.35	3	1.205	6	27
(K)QADPLSFLKDFmAG GISAAVSK(T)	95%	4.1538167	0.63102794		2,269.12	3	0.9767	6	27
(K)QIFmSGVDKK(T)	94%	2.5651858	0.117768064	0.1674910	1,167.79	2	0.1954	101	110
(K)SDGLTGLYR(G)	95%	2.8969731	0.366679	0.6575773	981.21	2	0.7171	171	179
(K)SDGLTGLYR(G)	95%	2.9623404	0.3725846	1.7212464	980.6267	2	0.1338	171	179
(K)SDGLTGLYR(G)	95%	3.2402637	0.32974854	2.09691	980.9974	2	0.5045	171	179
(K)SDGLTGLYR(G)	95%	3.1708739	0.34630764	1.79588	981.1648	2	0.6719	171	179
(K)SDGLTGLYR(G)	95%	3.236954	0.40901288		982.1716	2	1.679	171	179
(K)SDGLTGLYR(G)	95%	2.8853073	0.40152636	2.6777806	981.2542	2	0.7614	171	179
(K)SDGLTGLYR(G)	95%	3.0402927	0.39576077		980.6615	2	0.1687	171	179
(K)SDGLTGLYR(G)	95%	3.2444963	0.441903		982.1988	2	1.706	171	179
(K)SDGLTGLYR(G)	95%	3.126982	0.44131815		982.2372	2	1.744	171	179
(K)SDGLTGLYR(G)	95%	3.142921	0.40189195		981.141	2	0.6481	171	179
(K)SDGLTGLYR(G)	95%	3.2400322	0.40544719		981.1007	2	0.6078	171	179
(K)SDGLTGLYR(G)	95%	2.9411483	0.46037742		981.1952	2	0.7023	171	179
(K)SEGGGAFFK(G)	95%	2.5827186	0.18178935		898.9518	2	0.5332	266	274
(K)SEGGGAFFK(G)	95%	2.5991867	0.3347471		900.1838	2	1.765	266	274
(K)SEGGGAFFK(G)	95%	2.790689	0.2804622		899.1005	2	0.6819	266	274
(K)SEGGGAFFK(G)	95%	2.665983	0.32090628	0.2218487 3	898.6534	2	0.2348	266	274
(R)VKLLLQVQHVSK(Q)	95%	2.5701318	0.41371086		1,391.57	2	0.704	36	47

(R)VKLLLQVQHVSK(Q)	95%	3.384818	0.39104614	0.5850267	1,391.64	2	0.7777	36	47
(R)VKLLLQVQHVSK(Q)	95%	3.77958	0.32521456		1,391.53	3	0.6614	36	47
(R)VKLLLQVQHVSK(Q)	95%	3.5348885	0.44647592		1,391.38	2	0.5106	36	47
(R)VKLLLQVQHVSK(Q)	95%	3.5035737	0.46026847		1,391.35	2	0.4859	36	47
(R)VKLLLQVQHVSK(Q)	95%	3.9679744	0.43618923		1,391.52	2	0.6506	36	47
(R)VKLLLQVQHVSK(Q)	95%	4.3181944	0.43332735	1.5850266	1,390.90	2	0.03041	36	47
(R)VKLLLQVQHVSK(Q)	95%	4.032534	0.46206108	2.481486	1,392.59	2	1.724	36	47
(R)VKLLLQVQHVSK(Q)	95%	4.1387563	0.4186946	1.6382722	1,391.64	2	0.7781	36	47
(R)VKLLLQVQHVSK(Q)	95%	4.2510667	0.42959258		1,391.59	2	0.7233	36	47
(R)VKLLLQVQHVSK(Q)	95%	3.858033	0.4669999		1,391.80	2	0.9293	36	47
(R)VKLLLQVQHVSK(Q)	95%	3.726174	0.5035865	1.7212464	1,391.10	2	0.2338	36	47
(R)VKLLLQVQHVSK(Q)	95%	4.2975645	0.35626587		1,392.24	3	1.371	36	47
(R)VKLLLQVQHVSK(Q)	95%	4.5688443	0.47948417	3.0177288	1,392.48	2	1.613	36	47
(R)YFPTQALNFAFK(D)	95%	2.8733675	0.46242183	3.1870866	1,446.37	2	0.631	85	96
(R)YFPTQALNFAFK(D)	95%	3.1086001	0.453375	1.49485	1,447.16	2	1.422	85	96
(R)YFPTQALNFAFK(D)	95%	3.0242739	0.48888284	1.9208188	1,447.63	2	1.897	85	96
(R)YFPTQALNFAFK(D)	95%	3.223816	0.4478169	1.0409586	1,446.27	2	0.5348	85	96
(R)YFPTQALNFAFK(D)	95%	3.3965654	0.48711	2.8860567	1,446.77	2	1.035	85	96
(R)YFPTQALNFAFK(D)	95%	3.5790408	0.48655546	1.69897	1,446.18	2	0.4418	85	96
(R)YFPTQALNFAFK(D)	95%	3.391943	0.53939795	3.481486	1,445.95	2	0.217	85	96
(R)YFPTQALNFAFK(D)	95%	3.6611073	0.5709731	2.9208188	1,446.35	2	0.6141	85	96
(R)YFPTQALNFAFK(D)	95%	3.9719906	0.59835225	3.4317982	1,446.44	2	0.7076	85	96

c denotes Cys alkylation by iodoacetamide during trypsin digestion
m denotes Met oxidation
* As determined by Peptide Profit Algorithm embedded within Scaffold v 3.6.1

ArAAC peptides identified by LC-MS/MS on the LTQ Orbtrap Velos from gel bands of *Artemia franciscana* purified mitochondria in water and cleaned up using GE Healthcare 2D clean-up kit, as described in Materials and Methods

**Sequence**	**Prob***	**SEQUEST XCorr**	**SEQUEST deltaCn**	**Actual Mass**	**Charge**	**Delta PPM**	**Start**	**Stop**
(R)AAYFGFYDTVR(G)	95%	3.26823	0.45417878	1,308.62	2	1.954	193	203
(R)AAYFGFYDTVR(G)	95%	3.0094147	0.4891194	1,308.62	2	2.793	193	203
(R)AAYFGFYDTVR(G)	95%	3.3156369	0.58982927	1,308.62	2	2.327	193	203
(R)AKGDMmYK(G)	95%	1.9211068	0.26581827	958.4282	2	2.93	247	254
(R)AKGDMMYK(G)	95%	1.8451474	0.31497076	942.4327	2	2.377	247	254
(R)AKGDMmYK(G)	95%	1.7946675	0.38849276	958.4262	2	0.8945	247	254
(R)AKGDMMYK(G)	95%	1.8674057	0.39184296	942.4313	2	0.8895	247	254
(R)AKGDMmYK(G)	95%	1.8829086	0.37644872	958.4283	2	3.057	247	254
(R)AKGDMmYK(G)	95%	2.0713277	0.36094368	958.4271	2	1.785	247	254
(R)AKGDMmYK(G)	95%	1.72602	0.48351812	958.427	2	1.658	247	254
(R)AKGDMmYK(G)	95%	1.8137853	0.5217775	958.4267	2	1.403	247	254
(R)AKGDMMYK(G)	95%	2.1305318	0.46428525	942.4332	2	2.895	247	254
(R)AKGDMMYK(G)	95%	2.180959	0.51305044	942.4329	2	2.572	247	254
(R)AKGDMMYK(G)	95%	2.2479572	0.5352471	942.4339	2	3.671	247	254
(R)AKGDMMYK(G)	95%	2.816026	0.46700516	942.4332	2	2.895	247	254
(R)AKGDmmYKGTLDcWAK(I)	95%	2.3830447	0.42086238	1,905.85	3	2.853	247	262
(R)AKGDmmYKGTLDcWAK(I)	95%	2.5690813	0.41426766	1,905.84	3	-0.2202	247	262
(K)DFMAGGISAAVSK(T)	95%	3.0285628	0.18560374	1,252.62	2	2.733	15	27
(K)DFMAGGISAAVSK(T)	95%	2.208252	0.22325093	1,252.62	1	3.377	15	27
(K)DFmAGGISAAVSK(T)	95%	2.0335934	0.47916403	1,268.61	2	0.9351	15	27
(K)DFMAGGISAAVSK(T)	95%	3.1525285	0.31471935	1,252.61	2	2.052	15	27
(K)DFMAGGISAAVSK(T)	95%	2.741033	0.38999486	1,252.62	2	2.928	15	27
(K)DFMAGGISAAVSK(T)	95%	2.5937834	0.44466135	1,252.62	2	2.831	15	27
(K)DFmAGGISAAVSK(T)	95%	3.6343977	0.5587252	1,268.61	2	2.762	15	27
(K)DFmAGGISAAVSK(T)	95%	3.3478868	0.5891732	1,268.61	2	2.954	15	27
(K)DFMAGGISAAVSK(T)	95%	4.7033234	0.4973968	1,252.62	2	3.902	15	27
(K)DFMAGGISAAVSK(T)	95%	4.547151	0.5134883	1,252.62	2	2.344	15	27

(K)DFmAGGISAAVSK(T)	95%	4.4085135	0.5378743	1,268.61	2	2.858	15	27
(K)DFmAGGISAAVSK(T)	95%	4.7505674	0.5149047	1,268.61	2	1.608	15	27
(K)DFMAGGISAAVSK(T)	95%	4.7500825	0.5184115	1,252.62	2	2.636	15	27
(K)DFMAGGISAAVSK(T)	95%	4.9151773	0.5232863	1,252.62	2	3.025	15	27
(K)DFMAGGISAAVSK(T)	95%	5.002527	0.524624	1,252.62	2	2.928	15	27
(K)DFMAGGISAAVSK(T)	95%	4.864373	0.53463864	1,252.62	2	3.804	15	27
(K)DFMAGGISAAVSK(T)	95%	4.8977137	0.5347841	1,252.61	2	2.052	15	27
(K)DFmAGGISAAVSK(T)	95%	3.9687943	0.6134534	1,268.61	2	1.512	15	27
(K)DFmAGGISAAVSK(T)	95%	4.131709	0.60393167	1,268.61	2	4.396	15	27
(K)DFmAGGISAAVSK(T)	95%	4.3896956	0.59531057	1,268.61	2	4.492	15	27
(K)DFmAGGISAAVSK(T)	95%	4.3896956	0.59531057	1,268.61	2	4.492	15	27
(K)DFMAGGISAAVSK(T)	95%	4.491513	0.5962132	1,252.62	2	4.291	15	27
(K)DFmAGGISAAVSK(T)	95%	4.300345	0.61192673	1,268.61	2	0.8389	15	27
(K)DFmAGGISAAVSK(T)	95%	4.923465	0.57016426	1,268.61	2	2.57	15	27
(K)DFMAGGISAAVSK(T)	95%	4.899767	0.5732567	1,252.62	2	2.538	15	27
(K)DFMAGGISAAVSK(T)	95%	5.1283994	0.5655499	1,252.62	2	4.096	15	27
(K)DFmAGGISAAVSK(T)	95%	4.6409497	0.6051252	1,268.61	2	3.339	15	27
(K)DFmAGGISAAVSK(T)	95%	4.5820694	0.64478874	1,268.61	2	3.435	15	27
(K)DFmAGGISAAVSK(T)	95%	4.519508	0.65887827	1,268.61	2	2.473	15	27
(R)EFSGLGNcLVK(I)	95%	2.3094225	0.2648979	1,222.61	2	4.626	157	167
(R)EFSGLGNcLVK(I)	95%	1.9417251	0.2632014	1,222.61	1	3.49	157	167
(R)EFSGLGNcLVK(I)	95%	2.2424855	0.30642232	1,222.60	2	2.232	157	167
(R)EFSGLGNcLVK(I)	95%	2.41107	0.26345742	1,222.61	1	3.19	157	167
(R)EFSGLGNcLVK(I)	95%	2.5839791	0.2939407	1,222.60	2	1.733	157	167
(R)EFSGLGNcLVK(I)	95%	2.2982903	0.34547597	1,222.61	2	2.631	157	167
(R)EFSGLGNcLVK(I)	95%	2.4961128	0.31770596	1,222.61	2	3.329	157	167
(R)EFSGLGNcLVK(I)	95%	2.6569166	0.32130277	1,222.61	2	2.631	157	167
(R)EFSGLGNcLVK(I)	95%	2.503578	0.3455889	1,222.61	2	3.529	157	167
(R)EFSGLGNcLVK(I)	95%	3.1671617	0.35583425	1,222.61	1	3.29	157	167
(R)EFSGLGNcLVK(I)	95%	3.1242487	0.32257771	1,222.60	2	2.132	157	167
(R)EFSGLGNcLVK(I)	95%	3.0423028	0.36529338	1,222.61	2	4.825	157	167
(R)EFSGLGNcLVK(I)	95%	3.0423028	0.36529338	1,222.61	2	4.825	157	167

(R)EFSGLGNcLVK(I)	95%	2.8806245	0.3843126	1,222.60	2	1.633	157	167
(R)EFSGLGNcLVK(I)	95%	2.8237932	0.39029184	1,222.61	2	3.628	157	167
(R)EFSGLGNcLVK(I)	95%	3.0274894	0.40028307	1,222.60	2	0.9347	157	167
(R)EFSGLGNcLVK(I)	95%	3.1721356	0.3722158	1,222.61	2	4.526	157	167
(R)EFSGLGNcLVK(I)	95%	2.750312	0.41270375	1,222.60	2	2.531	157	167
(K)EQGVLSFWR(G)	95%	1.8654482	0.112292066	1,120.57	1	3.162	68	76
(K)GAAEREFSGLGNcLVK(I)	95%	2.4452436	0.16612883	1,706.85	3	3.259	152	167
(K)GAAEREFSGLGNcLVK(I)	95%	1.8016258	0.27719223	1,706.85	3	2.294	152	167
(K)GAAEREFSGLGNcLVK(I)	95%	2.2530408	0.22174977	1,706.85	3	4.224	152	167
(K)GAAEREFSGLGNcLVK(I)	95%	2.9818437	0.28099748	1,706.85	2	5.304	152	167
(K)GAAEREFSGLGNcLVK(I)	95%	2.9818437	0.28099748	1,706.85	2	5.304	152	167
(K)GAAEREFSGLGNcLVK(I)	95%	3.2633643	0.23109525	1,706.85	2	2.802	152	167
(K)GAAEREFSGLGNcLVK(I)	95%	4.038093	0.24638593	1,706.85	2	4.803	152	167
(K)GAAEREFSGLGNcLVK(I)	95%	4.0890465	0.27665582	1,706.85	2	4.661	152	167
(K)GAAEREFSGLGNcLVK(I)	95%	3.9745476	0.32194015	1,706.85	2	5.661	152	167
(K)GAAEREFSGLGNcLVK(I)	95%	3.7119896	0.306741	1,706.85	2	2.945	152	167
(K)GAAEREFSGLGNcLVK(I)	95%	3.9294262	0.32975015	1,706.85	2	4.303	152	167
(K)GAAEREFSGLGNcLVK(I)	95%	3.4440057	0.23859963	1,706.84	3	2.187	152	167
(K)GAAEREFSGLGNcLVK(I)	95%	3.1083264	0.28095347	1,706.85	3	4.653	152	167
(K)GAAEREFSGLGNcLVK(I)	95%	3.7687159	0.24731869	1,706.85	3	3.902	152	167
(K)GAAEREFSGLGNcLVK(I)	95%	4.466998	0.34037334	1,706.85	2	3.731	152	167
(K)GAAEREFSGLGNcLVK(I)	95%	2.8828528	0.31725103	1,706.85	3	4.117	152	167
(K)GAAEREFSGLGNcLVK(I)	95%	2.7014334	0.33140495	1,706.85	3	3.688	152	167
(K)GAAEREFSGLGNcLVK(I)	95%	2.6393564	0.3445151	1,706.85	3	2.508	152	167
(K)GAAEREFSGLGNcLVK(I)	95%	4.37305	0.39014897	1,706.85	2	3.374	152	167
(K)GAAEREFSGLGNcLVK(I)	95%	4.6190414	0.41634113	1,706.85	2	3.303	152	167
(K)GAAEREFSGLGNcLVK(I)	95%	3.106388	0.36980227	1,706.85	3	4.117	152	167
(K)GAAEREFSGLGNcLVK(I)	95%	3.654122	0.34653243	1,706.85	3	3.044	152	167
(K)GAAEREFSGLGNcLVK(I)	95%	3.3931608	0.39171672	1,706.84	3	2.187	152	167
(K)GAAEREFSGLGNcLVK(I)	95%	4.405793	0.37918937	1,706.85	3	2.401	152	167
(K)GAAEREFSGLGNcLVK(I)	95%	3.742601	0.45500302	1,706.85	3	4.331	152	167
(K)GAAEREFSGLGNcLVK(I)	95%	3.742601	0.45500302	1,706.85	3	4.331	152	167

(K)GALSNVFR(G)	95%	2.0569773	0.15554087	862.4685	1	2.806	275	282
(K)GALSNVFR(G)	95%	2.2451332	0.1273196	862.4685	1	2.806	275	282
(K)GALSNVFR(G)	95%	2.7619631	0.14919955	862.4693	2	3.709	275	282
(K)GALSNVFR(G)	95%	2.7598317	0.18478316	862.4673	2	1.448	275	282
(K)GALSNVFR(G)	95%	2.7359982	0.19995934	862.4675	2	1.66	275	282
(K)GALSNVFR(G)	95%	2.9344275	0.17512454	862.4696	2	4.063	275	282
(K)GALSNVFR(G)	95%	2.9344275	0.17512454	862.4696	2	4.063	275	282
(K)GALSNVFR(G)	95%	2.903832	0.19128443	862.47	2	4.558	275	282
(K)GALSNVFR(G)	95%	2.8629856	0.21644993	862.4695	2	3.922	275	282
(K)GALSNVFR(G)	95%	3.1006622	0.191205	862.4689	2	3.285	275	282
(K)GALSNVFR(G)	95%	3.1221	0.21405682	862.4681	2	2.296	275	282
(K)GALSNVFR(G)	95%	3.1510289	0.2340776	862.4685	2	2.791	275	282
(K)GDmmYKGTLDcWAK(I)	95%	2.309674	0.29713976	1,706.72	2	5.85	249	262
(K)GDmmYKGTLDcWAK(I)	95%	2.0057523	0.38754568	1,706.72	2	6.707	249	262
(K)GDmmYKGTLDcWAK(I)	95%	1.5244569	0.4667915	1,706.72	2	5.85	249	262
(K)GIIDcFVR(I)	95%	1.6774883	0.18196248	978.4978	1	1.996	57	64
(K)GIIDcFVR(I)	95%	1.314811	0.3581929	978.4996	1	3.803	57	64
(K)GIIDcFVR(I)	95%	2.4261308	0.2597658	978.4978	2	2.044	57	64
(K)GIIDcFVR(I)	95%	2.1762996	0.3263364	978.4989	2	3.103	57	64
(K)GIIDcFVR(I)	95%	2.3461108	0.37580213	978.5012	2	5.471	57	64
(K)GIIDcFVR(I)	95%	2.3461108	0.37580213	978.5012	2	5.471	57	64
(K)GIIDcFVR(I)	95%	2.5480976	0.2916404	978.4989	2	3.103	57	64
(K)GIIDcFVR(I)	95%	2.6701772	0.2872222	978.4992	2	3.477	57	64
(K)GIIDcFVR(I)	95%	2.4503956	0.35172087	978.4971	2	1.296	57	64
(K)GIIDcFVR(I)	95%	2.3963287	0.33688694	978.4984	2	2.667	57	64
(K)GIIDcFVR(I)	95%	2.3007643	0.36415035	978.4981	2	2.356	57	64
(K)GIIDcFVR(I)	95%	2.6262243	0.32225734	978.5002	2	4.412	57	64
(K)GIIDcFVR(I)	95%	2.423071	0.3734267	978.5006	2	4.848	57	64
(K)GIIDcFVR(I)	95%	2.3984053	0.3673634	978.4997	2	3.976	57	64
(K)GIIDcFVR(I)	95%	2.594352	0.35173455	978.4997	2	3.976	57	64
(K)GIIDcFVR(I)	95%	2.5249243	0.36517152	978.4986	2	2.792	57	64
(K)GIIDcFVR(I)	95%	2.4299047	0.39321563	978.5001	2	4.35	57	64

(K)GIIDcFVR(I)	95%	2.5750647	0.37968788	978.5003	2	4.599	57	64
(K)GIIDcFVR(I)	95%	2.4704792	0.3828673	978.499	2	3.228	57	64
(K)GIIDcFVR(I)	95%	2.7791889	0.37310302	978.5004	2	4.661	57	64
(K)GIIDcFVRIPK(E)	95%	2.0167637	0.30197158	1,316.73	2	1.684	57	67
(K)GIIDcFVRIPK(E)	95%	2.191051	0.31351706	1,316.73	2	2.703	57	67
(K)GIIDcFVRIPK(E)	95%	2.6524675	0.2698085	1,316.73	2	2.518	57	67
(K)GIIDcFVRIPK(E)	95%	3.1362457	0.2865892	1,316.73	2	4	57	67
(K)GIIDcFVRIPK(E)	95%	2.8010502	0.33530104	1,316.73	2	2.147	57	67
(K)GIIDcFVRIPK(E)	95%	2.984104	0.42571586	1,316.73	2	1.591	57	67
(K)GTLDcWAK(I)	95%	1.7667359	0.094155475	949.4361	1	3.376	255	262
(K)GTLDcWAK(I)	95%	2.0179818	0.2246657	949.4368	1	4.019	255	262
(K)GTLDcWAK(I)	95%	1.933182	0.22428532	949.4349	1	2.092	255	262
(K)GTLDcWAK(I)	95%	2.1378953	0.31169468	949.4359	1	3.12	255	262
(K)GTLDcWAK(I)	95%	2.3859875	0.3604208	949.4339	1	1.065	255	262
(K)GTLDcWAK(I)	95%	2.173585	0.4688415	949.433	2	0.02297	255	262
(K)GTLDcWAK(I)	95%	2.2945356	0.46333486	949.435	2	2.142	255	262
(K)GTLDcWAK(I)	95%	2.1662462	0.49840236	949.4353	2	2.463	255	262
(K)GTLDcWAK(I)	95%	2.1052341	0.5134004	949.4352	2	2.335	255	262
(K)GTLDcWAK(I)	95%	2.0924954	0.5327359	949.4344	2	1.5	255	262
(K)GTLDcWAK(I)	95%	2.1787388	0.5366245	949.4333	2	0.4083	255	262
(K)GTLDcWAK(I)	95%	2.3309963	0.53160614	949.4352	2	2.335	255	262
(K)GTLDcWAK(I)	95%	2.5280652	0.52930516	949.4331	2	0.2156	255	262
(K)GTLDcWAK(I)	95%	2.4259562	0.5393483	949.4364	2	3.619	255	262
(K)IFKSDGLTGLYR(G)	95%	2.045166	0.33726427	1,368.75	2	5.051	168	179
(K)IFKSDGLTGLYR(G)	95%	2.5054395	0.30796134	1,368.75	2	5.496	168	179
(K)IFKSDGLTGLYR(G)	95%	3.2333763	0.31008896	1,368.75	2	5.764	168	179
(K)IFKSDGLTGLYR(G)	95%	3.1533499	0.38375488	1,368.75	2	4.516	168	179
(K)IFKSDGLTGLYR(G)	95%	2.7940428	0.44829574	1,368.75	2	3.714	168	179
(K)IFKSDGLTGLYR(G)	95%	3.407968	0.38567567	1,368.75	2	3.536	168	179
(K)IFKSDGLTGLYR(G)	95%	3.008157	0.48479965	1,368.75	2	4.516	168	179
(K)IFKSDGLTGLYR(G)	95%	2.974032	0.5059611	1,368.75	2	3.625	168	179
(K)IFKSDGLTGLYR(G)	95%	2.8443847	0.4654012	1,368.74	3	2.256	168	179

(K)IFKSDGLTGLYR(G)	95%	2.3889668	0.5184917	1,368.75	3	3.86	168	179
(K)IFKSDGLTGLYR(G)	95%	3.956665	0.5373698	1,368.75	2	8.615	168	179
(K)IFKSDGLTGLYR(G)	95%	2.8778927	0.57866186	1,368.74	3	2.256	168	179
(K)IFKSDGLTGLYR(G)	95%	2.75096	0.61127317	1,368.74	3	2.991	168	179
(R)IPKEQGVLSFWR(G)	95%	2.3549488	0.14946355	1,458.80	3	2.58	65	76
(R)IPKEQGVLSFWR(G)	95%	3.6030285	0.051916674	1,458.80	2	3.154	65	76
(R)IPKEQGVLSFWR(G)	95%	2.933357	0.11587025	1,458.80	3	3.27	65	76
(R)IPKEQGVLSFWR(G)	95%	3.7588222	0.082399935	1,458.80	2	3.07	65	76
(R)IPKEQGVLSFWR(G)	95%	2.5263133	0.27898532	1,458.80	3	4.399	65	76
(K)IYKSEGGGAFFK(G)	95%	2.9561553	0.2213851	1,302.66	2	1.322	263	274
(K)IYKSEGGGAFFK(G)	95%	2.8419456	0.28342336	1,302.67	2	3.195	263	274
(K)IYKSEGGGAFFK(G)	95%	2.6974444	0.33752418	1,302.66	2	1.416	263	274
(K)IYKSEGGGAFFK(G)	95%	3.2959764	0.31134334	1,302.66	2	2.258	263	274
(K)IYKSEGGGAFFK(G)	95%	3.4907157	0.33288547	1,302.66	2	2.165	263	274
(K)IYKSEGGGAFFK(G)	95%	3.4279222	0.4181414	1,302.66	2	2.071	263	274
(K)IYKSEGGGAFFK(G)	95%	3.830049	0.44120666	1,302.66	2	2.165	263	274
(K)IYKSEGGGAFFK(G)	95%	2.4588618	0.422285	1,302.67	3	3.512	263	274
(K)IYKSEGGGAFFK(G)	95%	2.2718322	0.46781632	1,302.67	3	3.512	263	274
(K)KQADPLSFLK(D)	95%	2.2871552	0.24584484	1,145.65	2	3.05	5	14
(K)KQADPLSFLK(D)	95%	2.2940228	0.29611313	1,145.65	2	1.453	5	14
(K)KQADPLSFLK(D)	95%	2.2394838	0.29857105	1,145.65	2	2.198	5	14
(K)KQADPLSFLK(D)	95%	2.5649965	0.28087142	1,145.65	2	3.689	5	14
(K)KQADPLSFLK(D)	95%	2.1871092	0.36399248	1,145.65	2	1.666	5	14
(K)KQADPLSFLK(D)	95%	2.5497673	0.35555696	1,145.65	2	4.327	5	14
(K)KQADPLSFLK(D)	95%	2.4141061	0.37281722	1,145.65	2	2.198	5	14
(K)KQADPLSFLK(D)	95%	2.5307174	0.38284633	1,145.65	2	2.943	5	14
(K)KQADPLSFLK(D)	95%	3.0961785	0.43755153	1,145.65	2	3.369	5	14
(R)LAADTGKGAAER(E)	95%	2.1459796	0.33633643	1,158.60	2	3.361	145	156
(R)LAADTGKGAAER(E)	95%	2.4736407	0.33292714	1,158.60	2	0.7292	145	156
(R)LAADTGKGAAER(E)	95%	2.6638038	0.21805754	1,158.60	3	3.928	145	156
(R)LAADTGKGAAER(E)	95%	2.3243814	0.27730533	1,158.61	3	5.665	145	156
(R)LAADTGKGAAER(E)	95%	3.313622	0.33629468	1,158.60	2	1.992	145	156

(R)LAADTGKGAAER(E)	95%	3.4835083	0.34627733	1,158.60	2	1.361	145	156
(R)LAADTGKGAAER(E)	95%	2.384451	0.48397687	1,158.60	2	2.203	145	156
(R)LAADTGKGAAER(E)	95%	2.451476	0.51506406	1,158.60	2	2.203	145	156
(R)LAADTGKGAAER(E)	95%	4.02198	0.39855826	1,158.60	2	3.571	145	156
(R)LAADTGKGAAER(E)	95%	3.3746064	0.4900578	1,158.60	2	2.519	145	156
(R)LAADTGKGAAER(E)	95%	4.0418143	0.4274756	1,158.60	2	4.308	145	156
(R)LAADTGKGAAER(E)	95%	3.1276994	0.5359148	1,158.60	2	2.413	145	156
(R)LAADTGKGAAER(E)	95%	3.9730606	0.48260874	1,158.60	2	2.835	145	156
(R)LAADTGKGAAER(E)	95%	3.0509624	0.6071746	1,158.60	2	3.782	145	156
(R)LAADTGKGAAER(E)	95%	3.0509624	0.6071746	1,158.60	2	3.782	145	156
(R)LAADTGKGAAEREFSGLGNcLVK(I)	95%	2.2280962	0.26013467	2,363.20	2	2.845	145	167
(R)LAADTGKGAAEREFSGLGNcLVK(I)	95%	1.8092961	0.253279	2,363.20	4	2.989	145	167
(R)LAADTGKGAAEREFSGLGNcLVK(I)	95%	2.142276	0.2842935	2,363.20	4	4.641	145	167
(R)LAADTGKGAAEREFSGLGNcLVK(I)	95%	2.142276	0.2842935	2,363.20	4	4.641	145	167
(R)LAADTGKGAAEREFSGLGNcLVK(I)	95%	2.554701	0.24390517	2,363.20	3	2.168	145	167
(R)LAADTGKGAAEREFSGLGNcLVK(I)	95%	2.5594816	0.4541273	2,363.20	2	4.394	145	167
(R)LAADTGKGAAEREFSGLGNcLVK(I)	95%	2.3093734	0.47607094	2,363.20	2	3.052	145	167
(R)LAADTGKGAAEREFSGLGNcLVK(I)	95%	2.2879262	0.366729	2,363.20	3	5.034	145	167
(R)LAADTGKGAAEREFSGLGNcLVK(I)	95%	3.5366793	0.30859312	2,363.20	3	4.182	145	167
(R)LAADTGKGAAEREFSGLGNcLVK(I)	95%	2.816116	0.35801607	2,363.20	3	4.182	145	167
(R)LAADTGKGAAEREFSGLGNcLVK(I)	95%	2.6819198	0.3773429	2,363.19	3	1.781	145	167
(R)LAADTGKGAAEREFSGLGNcLVK(I)	95%	2.7156243	0.37603253	2,363.20	3	3.795	145	167
(R)LAADTGKGAAEREFSGLGNcLVK(I)	95%	3.8271255	0.50397456	2,363.20	2	5.014	145	167
(R)LAADTGKGAAEREFSGLGNcLVK(I)	95%	4.224577	0.5031056	2,363.20	2	4.497	145	167
(R)LAADTGKGAAEREFSGLGNcLVK(I)	95%	3.118948	0.39387918	2,363.20	3	4.956	145	167
(R)LAADTGKGAAEREFSGLGNcLVK(I)	95%	3.367704	0.4122039	2,363.20	3	4.724	145	167
(R)LAADTGKGAAEREFSGLGNcLVK(I)	95%	3.6019084	0.44552204	2,363.20	3	2.71	145	167
(R)LAADTGKGAAEREFSGLGNcLVK(I)	95%	2.8965204	0.50790864	2,363.20	3	2.4	145	167
(R)LAADTGKGAAEREFSGLGNcLVK(I)	95%	3.798461	0.4633279	2,363.20	3	3.64	145	167
(R)LAADTGKGAAEREFSGLGNcLVK(I)	95%	3.7291594	0.4955372	2,363.20	3	3.02	145	167
(R)LAADTGKGAAEREFSGLGNcLVK(I)	95%	4.195635	0.54356146	2,363.20	3	5.266	145	167
(R)LAADTGKGAAEREFSGLGNcLVK(I)	95%	4.195635	0.54356146	2,363.20	3	5.266	145	167

(K)LLLQVQHVSK(Q)	95%	1.8167666	0.24581501	1,163.71	3	4.175	38	47
(K)LLLQVQHVSK(Q)	95%	1.6204171	0.32111925	1,163.71	3	2.052	38	47
(K)LLLQVQHVSK(Q)	95%	2.7458613	0.3434574	1,163.70	2	0.2822	38	47
(K)LLLQVQHVSK(Q)	95%	2.2597134	0.47055694	1,163.70	2	0.9111	38	47
(K)LLLQVQHVSK(Q)	95%	1.691212	0.4025477	1,163.71	3	3.467	38	47
(K)LLLQVQHVSK(Q)	95%	3.0365105	0.45008957	1,163.70	2	0.4919	38	47
(K)LLLQVQHVSK(Q)	95%	3.107173	0.46666703	1,163.70	2	0.3871	38	47
(K)LLLQVQHVSK(Q)	95%	2.6608198	0.500888	1,163.71	2	1.959	38	47
(K)LLLQVQHVSK(Q)	95%	3.0267396	0.50527006	1,163.70	2	0.9111	38	47
(K)LLLQVQHVSK(Q)	95%	3.106171	0.4987399	1,163.71	2	2.378	38	47
(K)LLLQVQHVSK(Q)	95%	3.4728572	0.46019492	1,163.71	2	1.959	38	47
(K)LLLQVQHVSK(Q)	95%	3.4728572	0.46019492	1,163.71	2	1.959	38	47
(K)LLLQVQHVSK(Q)	95%	3.0013657	0.53167766	1,163.70	2	0.4919	38	47
(K)LLLQVQHVSK(Q)	95%	3.0295057	0.550219	1,163.71	2	2.483	38	47
(K)LLLQVQHVSK(Q)	95%	3.4380267	0.5046062	1,163.71	2	2.483	38	47
(K)LLLQVQHVSK(Q)	95%	3.5652928	0.5123133	1,163.71	2	2.902	38	47
(K)LLLQVQHVSK(Q)	95%	2.9855285	0.58790326	1,163.70	2	0.5967	38	47
(R)MMMQSGR(A)	95%	2.151073	0.28708908	839.3489	2	4.334	240	246
(R)MMMQSGR(A)	95%	2.0529437	0.3124344	839.3484	2	3.753	240	246
(R)MMMQSGR(A)	95%	1.6186733	0.39348954	839.3481	1	3.333	240	246
(R)MMMQSGR(A)	95%	2.088213	0.35354787	839.3482	2	3.462	240	246
(R)MMMQSGR(A)	95%	2.2012458	0.34781867	839.3485	2	3.825	240	246
(R)MMMQSGR(A)	95%	2.1801987	0.353712	839.3482	2	3.462	240	246
(K)QADPLSFLK(D)	95%	2.2525914	0.21058534	1,017.55	2	4.301	6	14
(K)QADPLSFLK(D)	95%	1.5208822	0.18504305	1,017.55	1	2.756	6	14
(K)QADPLSFLK(D)	95%	1.8567784	0.2855325	1,017.55	2	2.443	6	14
(K)QADPLSFLK(D)	95%	2.254757	0.20878536	1,017.55	2	2.563	6	14
(K)QADPLSFLK(D)	95%	1.9788125	0.31745973	1,017.55	2	1.904	6	14
(K)QIAVDKQYK(G)	95%	1.9295788	0.3809406	1,091.60	2	0.0751	48	56
(K)QIFMSGVDKK(T)	95%	2.2138839	0.29513454	1,151.60	2	1.146	101	110
(K)QIFMSGVDKK(T)	95%	2.473241	0.31495303	1,151.60	2	0.934	101	110
(R)RMMMQSGR(A)	95%	1.8329575	0.28735536	995.449	2	2.649	239	246

(R)RMMMQSGR(A)	95%	2.433125	0.29860568	995.45	2	3.629	239	246
(R)RMMMQSGR(A)	95%	2.6532092	0.30708092	995.4496	2	3.2	239	246
(K)SDGLTGLYR(G)	95%	1.5380224	0.45109108	980.4947	1	1.903	171	179
(K)SDGLTGLYR(G)	95%	1.6980176	0.39907396	980.4955	1	2.712	171	179
(K)SDGLTGLYR(G)	95%	2.1088145	0.52104914	980.4961	1	3.334	171	179
(K)SDGLTGLYR(G)	95%	2.756315	0.36312503	980.4959	2	3.133	171	179
(K)SDGLTGLYR(G)	95%	3.0709243	0.35889238	980.4943	2	1.454	171	179
(K)SDGLTGLYR(G)	95%	3.1738179	0.36172277	980.4966	2	3.817	171	179
(K)SDGLTGLYR(G)	95%	3.0923774	0.44380572	980.4956	2	2.76	171	179
(K)SDGLTGLYR(G)	95%	3.5502076	0.41070652	980.497	2	4.253	171	179
(K)SDGLTGLYR(G)	95%	3.3145063	0.4476359	980.4953	2	2.449	171	179
(K)SDGLTGLYR(G)	95%	3.278986	0.46226698	980.4958	2	3.009	171	179
(K)SEGGGAFFK(G)	95%	2.0938313	0.25031886	898.4222	2	3.985	266	274
(K)SEGGGAFFK(G)	95%	2.3153079	0.20134392	898.4219	1	3.66	266	274
(K)SEGGGAFFK(G)	95%	2.2265038	0.23105419	898.4222	1	4	266	274
(K)SEGGGAFFK(G)	95%	2.1720958	0.28768495	898.4205	2	2.152	266	274
(K)SEGGGAFFK(G)	95%	2.2598393	0.27298042	898.4212	2	2.967	266	274
(K)SEGGGAFFK(G)	95%	2.7065449	0.2325076	898.4223	2	4.12	266	274
(K)SEGGGAFFK(G)	95%	2.7065449	0.2325076	898.4223	2	4.12	266	274
(K)SEGGGAFFK(G)	95%	3.0042176	0.19144022	898.4206	2	2.22	266	274
(K)SEGGGAFFK(G)	95%	2.2872677	0.30964255	898.4219	2	3.713	266	274
(K)SEGGGAFFK(G)	95%	2.5403407	0.32200748	898.4222	2	4.053	266	274
(K)SEGGGAFFK(G)	95%	2.5459583	0.3543918	898.4206	2	2.22	266	274
(K)SEGGGAFFK(G)	95%	2.9883082	0.33217207	898.4204	2	2.017	266	274
(K)SEGGGAFFK(G)	95%	2.9301395	0.3579876	898.4218	2	3.577	266	274
(K)TAVAPIER(V)	95%	1.6545573	0.2474457	855.4838	1	2.735	28	35
(K)TAVAPIER(V)	95%	2.0701282	0.28895158	855.4855	1	4.659	28	35
(K)TAVAPIER(V)	95%	2.0389013	0.2652514	855.4844	1	3.377	28	35
(K)TAVAPIERVKLLLQVQHVSK(Q)	95%	1.3533212	0.43896043	2,228.33	4	-4.693	28	47
(K)TQFWR(W)	95%	1.5729802	0.12227875	736.3679	1	3.034	111	115
(R)TRLAADTGK(G)	95%	2.45689	0.29494327	931.511	2	2.241	143	151
(R)TRLAADTGKGAAER(E)	95%	2.6850135	0.342153	1,415.75	2	3.529	143	156

(R)VKLLLQVQHVSK(Q)	95%	2.152081	0.34723023	1,390.87	2	3.431	36	47
(R)VKLLLQVQHVSK(Q)	95%	1.890763	0.41674367	1,390.87	2	2.378	36	47
(R)VKLLLQVQHVSK(Q)	95%	2.2835886	0.37392658	1,390.87	2	2.642	36	47
(R)VKLLLQVQHVSK(Q)	95%	2.3371384	0.40215757	1,390.87	2	2.291	36	47
(R)VKLLLQVQHVSK(Q)	95%	2.1071303	0.3438931	1,390.86	3	-1.249	36	47
(R)VKLLLQVQHVSK(Q)	95%	2.7994046	0.51491934	1,390.87	2	1.852	36	47
(R)VKLLLQVQHVSK(Q)	95%	3.80194	0.5557729	1,390.87	2	1.589	36	47
(R)VKLLLQVQHVSK(Q)	95%	2.9625564	0.52748966	1,390.87	3	1.448	36	47
(R)VKLLLQVQHVSK(Q)	95%	2.7630346	0.61255574	1,390.87	3	1.448	36	47
(R)VKLLLQVQHVSKQIAVDK(Q)	95%	0.5979448	1	2,045.26	4	9.618	36	53
(R)YFPTQALNFAFK(D)	95%	1.7680225	0.42183793	1,445.74	2	4.204	85	96
(R)YFPTQALNFAFK(D)	95%	2.0042102	0.46596962	1,445.74	2	3.529	85	96
(R)YFPTQALNFAFK(D)	95%	2.1511598	0.455163	1,445.74	2	4.035	85	96
(R)YFPTQALNFAFK(D)	95%	2.168924	0.51016927	1,445.74	2	4.373	85	96
(R)YFPTQALNFAFK(D)	95%	3.1327176	0.46331552	1,445.74	2	3.782	85	96
(R)YFPTQALNFAFK(D)	95%	3.3638873	0.50901103	1,445.74	2	2.685	85	96

c denotes Cys alkylation by iodoacetamide during trypsin digestion

m denotes Met oxidation

* As determined by Peptide Profit Algorithm embedded within Scaffold v 3.6.1

ArAAC peptides identified by LC-MS/MS on the LTQ Orbtrap Velos from gel bands of *Artemia franciscana* purified mitochondria in water and delipitated, as described in Materials and Methods

Sequence	Prob*	SEQUEST XCorr	SEQUEST deltaCn	Actual Mass	Charge	Delta PPM	Start	Stop
(R)AAYFGFYDTVR(G)	95%	3.142321	0.3652899	1,308.62	2	1.581	193	203
(R)AAYFGFYDTVR(G)	95%	3.0237992	0.3856338	1,308.62	2	2.42	193	203
(R)AAYFGFYDTVR(G)	95%	2.976618	0.43218172	1,308.61	2	0.6489	193	203
(R)AAYFGFYDTVR(G)	95%	3.4770906	0.45511138	1,308.62	2	1.488	193	203
(R)AAYFGFYDTVR(G)	95%	3.6218169	0.48661083	1,308.62	2	2.047	193	203
(R)AKGDMMYK(G)	95%	1.916933	0.3521649	942.4307	2	0.2426	247	254
(R)AKGDmMYK(G)	95%	2.1680138	0.29125282	958.4262	2	0.8945	247	254
(R)AKGDmMYK(G)	95%	2.2290268	0.28240758	958.4262	2	0.8945	247	254
(R)AKGDmMYK(G)	95%	2.3197799	0.27856952	958.4259	2	0.5129	247	254
(R)AKGDMMYK(G)	95%	2.032211	0.36416814	942.4308	2	0.3072	247	254
(R)AKGDMMYK(G)	95%	2.260559	0.40514705	942.4309	2	0.5013	247	254
(R)AKGDmMYK(G)	95%	2.352393	0.46846193	958.4257	2	0.322	247	254
(R)AKGDMMYK(G)	95%	2.3523147	0.48942053	942.4309	2	0.5013	247	254
(K)DFMAGGISAAVSK(T)	95%	4.0876694	0.39492133	1,252.62	1	3.766	15	27
(K)DFMAGGISAAVSK(T)	95%	3.2890177	0.28036663	1,252.62	2	3.22	15	27
(K)DFMAGGISAAVSK(T)	95%	3.9011097	0.48895344	1,252.61	1	0.9425	15	27
(K)DFmAGGISAAVSK(T)	95%	3.4255054	0.32523695	1,268.61	2	2.954	15	27
(K)DFmAGGISAAVSK(T)	95%	4.16477	0.5329157	1,268.61	1	2.532	15	27
(K)DFmAGGISAAVSK(T)	95%	4.187181	0.6145832	1,268.61	1	-0.0639	15	27
(K)DFmAGGISAAVSK(T)	95%	3.9654665	0.40859368	1,268.61	2	-1.757	15	27
(K)DFMAGGISAAVSK(T)	95%	4.388675	0.37888634	1,252.61	2	1.078	15	27
(K)DFMAGGISAAVSK(T)	95%	3.9886804	0.44895563	1,252.62	2	2.441	15	27
(K)DFmAGGISAAVSK(T)	95%	3.983812	0.46050292	1,268.61	2	1.031	15	27
(K)DFmAGGISAAVSK(T)	95%	4.2260995	0.44544157	1,268.61	2	0.8389	15	27
(K)DFMAGGISAAVSK(T)	95%	4.2685075	0.46570957	1,252.61	2	1.175	15	27
(K)DFMAGGISAAVSK(T)	95%	4.948319	0.43075645	1,252.61	2	1.37	15	27
(K)DFMAGGISAAVSK(T)	95%	4.6102905	0.51401997	1,252.61	2	0.2015	15	27

(K)DFMAGGISAAVSK(T)	95%	4.8085003	0.5026541	1,252.61	2	0.7857	15	27
(K)DFMAGGISAAVSK(T)	95%	4.1995254	0.5520639	1,252.61	2	1.565	15	27
(K)DFMAGGISAAVSK(T)	95%	4.539711	0.5308657	1,252.61	2	0.1041	15	27
(K)DFMAGGISAAVSK(T)	95%	4.653815	0.5302974	1,252.61	2	1.857	15	27
(K)DFMAGGISAAVSK(T)	95%	4.7567506	0.52413464	1,252.61	2	0.591	15	27
(K)DFMAGGISAAVSK(T)	95%	4.8735843	0.5264426	1,252.61	2	1.175	15	27
(K)DFMAGGISAAVSK(T)	95%	4.9335	0.5252787	1,252.61	2	0.6883	15	27
(K)DFMAGGISAAVSK(T)	95%	4.803438	0.5372895	1,252.61	2	1.467	15	27
(K)DFMAGGISAAVSK(T)	95%	4.850909	0.53546125	1,252.61	2	1.857	15	27
(K)DFMAGGISAAVSK(T)	95%	4.6977086	0.5490968	1,252.61	2	1.954	15	27
(K)DFmAGGISAAVSK(T)	95%	4.4158077	0.5715615	1,268.61	2	0.6466	15	27
(K)DFmAGGISAAVSK(T)	95%	4.4818187	0.5723353	1,268.60	2	-5.795	15	27
(K)DFmAGGISAAVSK(T)	95%	4.368699	0.5828252	1,268.60	2	-3.584	15	27
(K)DFMAGGISAAVSK(T)	95%	4.8892856	0.54828846	1,252.61	2	1.175	15	27
(K)DFMAGGISAAVSK(T)	95%	4.8243093	0.56760615	1,252.62	2	2.636	15	27
(K)DFmAGGISAAVSK(T)	95%	4.5043316	0.5927453	1,268.61	2	0.1659	15	27
(K)DFmAGGISAAVSK(T)	95%	4.692536	0.59013534	1,268.60	2	-5.507	15	27
(K)DFmAGGISAAVSK(T)	95%	4.866507	0.58791935	1,268.60	2	-2.238	15	27
(K)DFMAGGISAAVSK(T)	95%	5.0974545	0.5751012	1,252.61	2	1.759	15	27
(K)DFmAGGISAAVSK(T)	95%	4.7347026	0.60534495	1,268.60	2	-4.545	15	27
(K)DFmAGGISAAVSK(T)	95%	4.327834	0.6384741	1,268.61	2	-0.2187	15	27
(K)DFmAGGISAAVSK(T)	95%	5.1158805	0.58673954	1,268.61	2	1.993	15	27
(K)DFmAGGISAAVSK(T)	95%	4.7176847	0.6163727	1,268.60	2	-3.968	15	27
(K)DFmAGGISAAVSK(T)	95%	4.5816345	0.63164115	1,268.61	2	3.05	15	27
(K)DFmAGGISAAVSK(T)	95%	5.161056	0.5906102	1,268.60	2	-5.411	15	27
(K)DFmAGGISAAVSK(T)	95%	4.6569333	0.6413838	1,268.61	2	4.781	15	27
(K)DFmAGGISAAVSK(T)	95%	4.7857924	0.63793445	1,268.61	2	0.4543	15	27
(K)DFmAGGISAAVSK(T)	95%	4.932018	0.6325917	1,268.60	2	-5.218	15	27
(K)DFmAGGISAAVSK(T)	95%	4.6726875	0.66222394	1,268.61	2	0.262	15	27
(K)DFmAGGISAAVSK(T)	95%	4.762873	0.6656983	1,268.61	2	0.5505	15	27
(R)EFSGLGNcLVK(I)	95%	3.121315	0.311595	1,222.61	1	4.487	157	167
(R)EFSGLGNcLVK(I)	95%	2.7053754	0.2569607	1,222.60	2	0.6354	157	167

(R)EFSGLGNcLVK(I)	95%	2.812137	0.26654297	1,222.60	2	0.03684	157	167
(R)EFSGLGNcLVK(I)	95%	2.6857755	0.2740516	1,222.60	2	1.334	157	167
(R)EFSGLGNcLVK(I)	95%	2.7740061	0.2663291	1,222.60	2	1.833	157	167
(R)EFSGLGNcLVK(I)	95%	3.3940778	0.3418788	1,222.60	1	2.093	157	167
(R)EFSGLGNcLVK(I)	95%	2.7258735	0.3185289	1,222.60	2	0.9347	157	167
(R)EFSGLGNcLVK(I)	95%	2.6505928	0.33650845	1,222.60	2	1.434	157	167
(R)EFSGLGNcLVK(I)	95%	3.1204188	0.33682892	1,222.60	2	-3.056	157	167
(R)EFSGLGNcLVK(I)	95%	2.8444908	0.35741818	1,222.60	2	0.03684	157	167
(R)EFSGLGNcLVK(I)	95%	2.7397132	0.35345438	1,222.60	2	1.833	157	167
(R)EFSGLGNcLVK(I)	95%	2.9380329	0.34267494	1,222.60	2	1.932	157	167
(R)EFSGLGNcLVK(I)	95%	2.7499018	0.38087815	1,222.60	2	1.833	157	167
(R)EFSGLGNcLVK(I)	95%	3.0213804	0.36550424	1,222.60	2	0.4359	157	167
(R)EFSGLGNcLVK(I)	95%	3.0816822	0.40048078	1,222.60	2	-1.559	157	167
(R)EFSGLGNcLVK(I)	95%	3.2102127	0.36982957	1,222.60	2	0.6354	157	167
(R)EFSGLGNcLVK(I)	95%	3.1280425	0.36017027	1,222.60	2	2.232	157	167
(R)EFSGLGNcLVK(I)	95%	3.145099	0.37430152	1,222.60	2	2.032	157	167
(K)EQGVLSFWR(G)	95%	2.972288	0.13765158	1,120.57	2	1.354	68	76
(K)EQGVLSFWR(G)	95%	2.5624962	0.1727371	1,120.57	1	1.965	68	76
(K)GAAEREFSGLGNcLVK(I)	95%	3.117694	0.14218155	1,706.84	3	1.007	152	167
(K)GAAEREFSGLGNcLVK(I)	95%	4.371152	0.3258528	1,706.84	2	1.015	152	167
(K)GAAEREFSGLGNcLVK(I)	95%	4.1150546	0.3415709	1,706.84	3	1.436	152	167
(K)GAAEREFSGLGNcLVK(I)	95%	4.321554	0.39282593	1,706.84	3	1.758	152	167
(K)GALSNVFR(G)	95%	2.2652428	0.16778201	862.4675	1	1.605	275	282
(K)GALSNVFR(G)	95%	3.1193125	0.14823686	862.4676	2	1.73	275	282
(K)GALSNVFR(G)	95%	2.7507272	0.20852236	862.4676	2	1.801	275	282
(K)GALSNVFR(G)	95%	2.916705	0.18834464	862.4682	2	2.437	275	282
(K)GALSNVFR(G)	95%	2.9837139	0.19678076	862.4668	2	0.8114	275	282
(K)GALSNVFR(G)	95%	2.9622808	0.18685102	862.4674	2	1.589	275	282
(K)GALSNVFR(G)	95%	3.044618	0.19123138	862.467	2	1.094	275	282
(K)GALSNVFR(G)	95%	2.9524312	0.2091366	862.4667	2	0.7407	275	282
(K)GALSNVFR(G)	95%	2.9587905	0.20247707	862.4671	2	1.236	275	282
(K)GALSNVFR(G)	95%	3.2561135	0.20221862	862.4663	2	0.2459	275	282

(K)GALSNVFR(G)	95%	3.0933275	0.19980891	862.4671	2	1.165	275	282
(K)GALSNVFR(G)	95%	3.1764905	0.19081004	862.4671	2	1.165	275	282
(K)GALSNVFR(G)	95%	2.7364523	0.2453014	862.4683	2	2.579	275	282
(K)GALSNVFR(G)	95%	3.0907497	0.21656276	862.4692	2	3.639	275	282
(K)GALSNVFR(G)	95%	3.0870585	0.21857943	862.467	2	1.094	275	282
(K)GALSNVFR(G)	95%	3.2306888	0.18732288	862.4676	2	1.73	275	282
(K)GALSNVFR(G)	95%	3.2216284	0.22048558	862.4674	2	1.589	275	282
(K)GALSNVFR(G)	95%	3.0877695	0.27805296	862.4674	2	1.518	275	282
(K)GDmmYKGTLDcWAK(I)	95%	2.5681708	0.32985047	1,706.72	2	6.707	249	262
(K)GDmmYKGTLDcWAK(I)	95%	2.7754817	0.40587592	1,706.72	2	5.278	249	262
(K)GIIDcFVR(I)	95%	2.3333035	0.2979941	978.4983	2	2.543	57	64
(K)GIIDcFVR(I)	95%	2.3837807	0.29539934	978.4978	2	2.044	57	64
(K)GIIDcFVR(I)	95%	2.335316	0.3278819	978.4972	2	1.359	57	64
(K)GIIDcFVR(I)	95%	2.5474901	0.293841	978.4979	2	2.106	57	64
(K)GIIDcFVR(I)	95%	2.5897956	0.38255268	978.5007	2	4.973	57	64
(K)GIIDcFVR(I)	95%	2.4889064	0.35739154	978.4973	2	1.546	57	64
(K)GIIDcFVR(I)	95%	2.5448563	0.40011635	978.5006	2	4.91	57	64
(K)GIIDcFVR(I)	95%	2.508252	0.36560836	978.4975	2	1.67	57	64
(K)GIIDcFVR(I)	95%	2.6079252	0.35538012	978.4972	2	1.359	57	64
(K)GIIDcFVR(I)	95%	2.7073588	0.34022114	978.4976	2	1.795	57	64
(K)GIIDcFVR(I)	95%	2.4789991	0.37440625	978.4978	2	2.044	57	64
(K)GIIDcFVR(I)	95%	2.6128633	0.36710134	978.4971	2	1.296	57	64
(K)GIIDcFVR(I)	95%	2.5847244	0.36402038	978.4981	2	2.356	57	64
(K)GIIDcFVR(I)	95%	2.6461055	0.3705744	978.4988	2	2.979	57	64
(K)GIIDcFVR(I)	95%	2.5451977	0.38597322	978.4981	2	2.356	57	64
(K)GIIDcFVR(I)	95%	2.5865407	0.38447744	978.4981	2	2.293	57	64
(K)GIIDcFVR(I)	95%	2.537931	0.40960562	978.4976	2	1.795	57	64
(R)GTGGALVLVFYDELK(V)	95%	5.0987554	0.519912	1,580.85	2	3.112	283	297
(R)GTGGALVLVFYDELK(V)	95%	5.046594	0.5406403	1,580.85	2	2.572	283	297
(R)GTGGALVLVFYDELK(V)	95%	5.1603694	0.59192175	1,580.85	2	1.955	283	297
(R)GTGGALVLVFYDELK(V)	95%	4.967757	0.63403934	1,580.85	2	2.804	283	297
(K)GTLDcWAK(I)	95%	2.260623	0.36779788	949.4317	2	-1.326	255	262

(K)GTLDcWAK(I)	95%	2.125865	0.45314205	949.4305	2	-2.61	255	262
(K)GTLDcWAK(I)	95%	2.2315283	0.46658674	949.4317	2	-1.326	255	262
(K)GTLDcWAK(I)	95%	2.4722204	0.46375155	949.4323	2	-0.6834	255	262
(K)GTLDcWAK(I)	95%	2.172934	0.5265687	949.4312	2	-1.775	255	262
(K)GTLDcWAK(I)	95%	2.6452315	0.4595383	949.4312	2	-1.775	255	262
(K)GTLDcWAK(I)	95%	2.2546694	0.5144871	949.4326	2	-0.3623	255	262
(K)GTLDcWAK(I)	95%	2.3230662	0.5430095	949.43	2	-3.124	255	262
(K)GTLDcWAK(I)	95%	2.6162786	0.5824131	949.4327	2	-0.2981	255	262
(K)IFKSDGLTGLYR(G)	95%	3.6841533	0.33690682	1,368.74	2	-3.059	168	179
(K)IFKSDGLTGLYR(G)	95%	4.106658	0.54274756	1,368.75	2	3.981	168	179
(K)IFKSDGLTGLYR(G)	95%	3.3452659	0.55274	1,368.74	3	-3.158	168	179
(R)IPKEQGVLSFWR(G)	95%	2.446274	0.22250299	1,458.80	3	2.141	65	76
(R)IPKEQGVLSFWR(G)	95%	3.6032717	0.2158072	1,458.80	3	0.6989	65	76
(R)IPKEQGVLSFWR(G)	95%	2.9941854	0.26616606	1,458.80	3	0.4481	65	76
(K)IYKSEGGGAFFK(G)	95%	3.2606273	0.40866867	1,302.66	2	0.292	263	274
(K)KQADPLSFLK(D)	95%	2.4536638	0.14967188	1,145.65	3	3.091	5	14
(K)KQADPLSFLK(D)	95%	2.5978756	0.31451797	1,145.65	2	0.4948	5	14
(K)KQADPLSFLK(D)	95%	2.7830558	0.35577706	1,145.65	2	0.4948	5	14
(K)KQADPLSFLK(D)	95%	2.6216283	0.39411473	1,145.65	2	1.559	5	14
(K)KQADPLSFLK(D)	95%	2.6567438	0.45525816	1,145.65	2	1.453	5	14
(K)KQADPLSFLK(D)	95%	3.0568607	0.421947	1,145.65	2	1.24	5	14
(K)KQADPLSFLKDFmAGGISAAVSK(T)	95%	2.606698	0.29773036	2,396.24	4	1.169	5	27
(K)KTQFWR(W)	95%	2.4145565	0.15945639	864.4618	2	1.337	110	115
(R)LAADTGKGAAER(E)	95%	2.5518398	0.27576932	1,158.60	2	0.9397	145	156
(R)LAADTGKGAAER(E)	95%	3.3051949	0.37090218	1,158.60	2	-0.113	145	156
(R)LAADTGKGAAER(E)	95%	3.4426582	0.40589872	1,158.60	2	0.9397	145	156
(K)LLLQVQHVSK(Q)	95%	1.4148202	0.3008418	1,163.70	3	-1.013	38	47
(K)LLLQVQHVSK(Q)	95%	2.3512015	0.3155458	1,163.70	1	0.6606	38	47
(K)LLLQVQHVSK(Q)	95%	1.8683746	0.27334926	1,163.70	3	-1.328	38	47
(K)LLLQVQHVSK(Q)	95%	2.850223	0.31007522	1,163.70	2	-2.548	38	47
(K)LLLQVQHVSK(Q)	95%	1.6511687	0.3093996	1,163.70	3	1.345	38	47
(K)LLLQVQHVSK(Q)	95%	2.8831468	0.349887	1,163.70	2	-2.548	38	47

(K)LLLQVQHVSK(Q)	95%	1.5936797	0.342301	1,163.70	3	1.109	38	47
(K)LLLQVQHVSK(Q)	95%	1.88618	0.33200145	1,163.70	3	-0.1485	38	47
(K)LLLQVQHVSK(Q)	95%	3.08753	0.38265842	1,163.70	2	-1.499	38	47
(K)LLLQVQHVSK(Q)	95%	2.4454544	0.28816006	1,163.70	3	-0.856	38	47
(K)LLLQVQHVSK(Q)	95%	3.3546255	0.37184572	1,163.70	2	-3.805	38	47
(K)LLLQVQHVSK(Q)	95%	3.4036531	0.3836543	1,163.70	2	-2.338	38	47
(K)LLLQVQHVSK(Q)	95%	2.909807	0.45963377	1,163.70	2	-2.338	38	47
(K)LLLQVQHVSK(Q)	95%	3.2881522	0.4158023	1,163.70	2	-2.233	38	47
(K)LLLQVQHVSK(Q)	95%	3.3162446	0.41500974	1,163.70	2	-2.338	38	47
(K)LLLQVQHVSK(Q)	95%	3.1468353	0.43301967	1,163.70	2	-0.8706	38	47
(K)LLLQVQHVSK(Q)	95%	3.333601	0.4339041	1,163.70	2	-2.128	38	47
(K)LLLQVQHVSK(Q)	95%	3.6781704	0.41440842	1,163.70	2	-1.919	38	47
(K)LLLQVQHVSK(Q)	95%	3.416121	0.45738307	1,163.70	2	-2.757	38	47
(K)LLLQVQHVSK(Q)	95%	3.3624067	0.46860835	1,163.70	2	-2.652	38	47
(K)LLLQVQHVSK(Q)	95%	3.6343434	0.44891092	1,163.70	2	-2.338	38	47
(K)LLLQVQHVSK(Q)	95%	3.249681	0.500636	1,163.70	2	-2.338	38	47
(K)LLLQVQHVSK(Q)	95%	3.361763	0.5137398	1,163.71	2	2.798	38	47
(K)LLLQVQHVSK(Q)	95%	2.2725613	0.45429626	1,163.71	3	2.524	38	47
(K)LLLQVQHVSK(Q)	95%	3.75647	0.5266346	1,163.71	2	7.619	38	47
(R)MmMQSGR(A)	95%	2.3592134	0.3722652	855.3413	2	1.353	240	246
(K)QADPLSFLK(D)	95%	2.1255314	0.3030262	1,017.55	2	0.9454	6	14
(K)QADPLSFLK(D)	95%	2.2182477	0.31506196	1,017.55	2	0.6458	6	14
(K)QADPLSFLKDFMAGGISAAVSK(T)	95%	1.8523489	0.4220227	2,252.15	2	1.342	6	27
(K)QADPLSFLKDFmAGGISAAVSK(T)	95%	2.2805061	0.29976067	2,268.15	3	1.281	6	27
(K)QADPLSFLKDFmAGGISAAVSK(T)	95%	3.04432	0.3609895	2,268.15	3	3.298	6	27
(K)QADPLSFLKDFmAGGISAAVSK(T)	95%	3.3490088	0.36378607	2,268.15	3	2.572	6	27
(K)QADPLSFLKDFmAGGISAAVSK(T)	95%	3.542175	0.3793795	2,268.15	3	3.621	6	27
(K)QADPLSFLKDFmAGGISAAVSK(T)	95%	3.6670575	0.38949203	2,268.14	3	-2.431	6	27
(K)QADPLSFLKDFMAGGISAAVSK(T)	95%	3.4718678	0.48577818	2,252.15	3	0.6854	6	27
(K)QADPLSFLKDFMAGGISAAVSK(T)	95%	3.5556023	0.5006016	2,252.15	3	0.848	6	27
(K)QADPLSFLKDFMAGGISAAVSK(T)	95%	3.537211	0.51000637	2,252.16	3	2.555	6	27
(K)QIAVDKQYK(G)	95%	2.2164557	0.31657526	1,091.60	2	1.304	48	56

(K)QIFmSGVDK(K)	95%	1.98146	0.28932846	1,039.50	2	1.257	101	109
(K)QIFmSGVDKK(T)	95%	2.0544732	0.3172998	1,167.60	2	0.1541	101	110
(K)QIFMSGVDKK(T)	95%	2.4907587	0.31306824	1,151.60	2	-0.2309	101	110
(K)QIFmSGVDKK(T)	95%	1.9856675	0.23431659	1,167.60	3	1.396	101	110
(K)QIFmSGVDKK(T)	95%	2.0270536	0.28691277	1,167.60	3	1.788	101	110
(K)QIFMSGVDKK(T)	95%	2.553006	0.34393206	1,151.61	3	3.411	101	110
(K)SDGLTGLYR(G)	95%	1.880261	0.21997535	980.4944	1	1.53	171	179
(K)SDGLTGLYR(G)	95%	1.8210571	0.24711521	980.4943	1	1.468	171	179
(K)SDGLTGLYR(G)	95%	1.8264146	0.28441495	980.4953	1	2.463	171	179
(K)SDGLTGLYR(G)	95%	1.5245644	0.4386785	980.4938	1	0.9706	171	179
(K)SDGLTGLYR(G)	95%	1.9603931	0.38031584	980.4939	1	1.095	171	179
(K)SDGLTGLYR(G)	95%	2.3220716	0.44886506	980.4947	1	1.903	171	179
(K)SDGLTGLYR(G)	95%	3.1578383	0.2795435	980.4928	2	-0.1003	171	179
(K)SDGLTGLYR(G)	95%	2.8586264	0.34637457	980.494	2	1.143	171	179
(K)SDGLTGLYR(G)	95%	2.8162847	0.37501678	980.4932	2	0.335	171	179
(K)SDGLTGLYR(G)	95%	3.0422966	0.34132105	980.4937	2	0.8947	171	179
(K)SDGLTGLYR(G)	95%	2.986974	0.3651216	980.4936	2	0.7703	171	179
(K)SDGLTGLYR(G)	95%	3.091876	0.37340096	980.4934	2	0.5838	171	179
(K)SDGLTGLYR(G)	95%	3.134792	0.35829547	980.4947	2	1.89	171	179
(K)SDGLTGLYR(G)	95%	3.2381048	0.3652233	980.4932	2	0.335	171	179
(K)SDGLTGLYR(G)	95%	2.8499088	0.4081226	980.4941	2	1.206	171	179
(K)SDGLTGLYR(G)	95%	3.0536397	0.4027787	980.4935	2	0.646	171	179
(K)SDGLTGLYR(G)	95%	3.2668247	0.37272188	980.4948	2	2.014	171	179
(K)SDGLTGLYR(G)	95%	3.0267122	0.42235512	980.4941	2	1.206	171	179
(K)SDGLTGLYR(G)	95%	3.1397917	0.42806375	980.4936	2	0.7081	171	179
(K)SDGLTGLYR(G)	95%	3.3946438	0.40079433	980.4943	2	1.454	171	179
(K)SDGLTGLYR(G)	95%	3.184258	0.4413017	980.4934	2	0.5216	171	179
(K)SDGLTGLYR(G)	95%	3.2316856	0.436572	980.4941	2	1.268	171	179
(K)SDGLTGLYR(G)	95%	3.1526546	0.4956615	980.4934	2	0.5838	171	179
(K)SEGGGAFFK(G)	95%	2.3248131	0.185305	898.4196	1	1.149	266	274
(K)SEGGGAFFK(G)	95%	2.2700572	0.23245499	898.4197	1	1.285	266	274
(K)SEGGGAFFK(G)	95%	2.687862	0.24823195	898.4189	2	0.3881	266	274

(K)SEGGGAFFK(G)	95%	2.7001462	0.27707043	898.4181	2	-0.4941	266	274
(K)SEGGGAFFK(G)	95%	2.750596	0.25941497	898.4186	2	0.04878	266	274
(K)SEGGGAFFK(G)	95%	2.6905785	0.26209325	898.4191	2	0.5917	266	274
(K)SEGGGAFFK(G)	95%	2.8115501	0.22881848	898.4196	2	1.135	266	274
(K)SEGGGAFFK(G)	95%	2.9540677	0.22563	898.4193	2	0.7952	266	274
(K)SEGGGAFFK(G)	95%	2.9930015	0.2796686	898.4186	2	0.04878	266	274
(K)SEGGGAFFK(G)	95%	2.8562844	0.30174038	898.4193	2	0.7952	266	274
(K)SEGGGAFFK(G)	95%	2.9149928	0.30327287	898.4192	2	0.7274	266	274
(K)SEGGGAFFK(G)	95%	3.163952	0.31624758	898.4183	2	-0.2905	266	274
(K)SEGGGAFFK(G)	95%	2.912985	0.36457014	898.4186	2	-0.01908	266	274
(K)SEGGGAFFK(G)	95%	2.906483	0.36988732	898.4212	2	2.899	266	274
(K)SEGGGAFFK(G)	95%	3.1099555	0.3577726	898.4197	2	1.27	266	274
(K)SEGGGAFFK(G)	95%	3.4074824	0.34493962	898.4189	2	0.3202	266	274
(K)TAVAPIER(V)	95%	2.1574175	0.2920231	855.4815	2	0.01177	28	35
(K)TAVAPIER(V)	95%	2.1429062	0.25972563	855.4831	1	1.88	28	35
(K)TAVAPIER(V)	95%	2.3030756	0.32470867	855.4816	2	0.1543	28	35
(K)TAVAPIER(V)	95%	2.2833104	0.33677164	855.4827	2	1.437	28	35
(K)TAVAPIER(V)	95%	2.3247323	0.34652075	855.4819	2	0.5106	28	35
(R)VKLLLQVQHVSK(Q)	95%	2.179251	0.30061564	1,390.87	3	0.264	36	47
(R)VKLLLQVQHVSK(Q)	95%	2.655842	0.2722635	1,390.87	3	0.1982	36	47
(R)VKLLLQVQHVSK(Q)	95%	3.0014176	0.38703468	1,390.87	2	2.992	36	47
(R)VKLLLQVQHVSK(Q)	95%	3.153056	0.40550944	1,390.87	2	-0.2526	36	47
(R)VKLLLQVQHVSK(Q)	95%	2.051459	0.38551554	1,390.87	3	-0.4596	36	47
(R)VKLLLQVQHVSK(Q)	95%	2.1333966	0.39432186	1,390.87	3	2.961	36	47
(R)VKLLLQVQHVSK(Q)	95%	2.9184582	0.41186535	1,390.87	3	0.0008825	36	47
(R)VKLLLQVQHVSK(Q)	95%	3.2571995	0.41858232	1,390.86	3	-1.183	36	47
(R)VKLLLQVQHVSK(Q)	95%	3.4422417	0.5389501	1,390.87	2	0.2736	36	47
(R)VKLLLQVQHVSK(Q)	95%	4.083135	0.5617138	1,390.87	2	-0.5157	36	47
(R)VKLLLQVQHVSK(Q)	95%	3.0820923	0.56501836	1,390.87	3	0.5271	36	47
(R)VKLLLQVQHVSK(Q)	95%	3.4069476	0.5688973	1,390.86	3	-0.9858	36	47
(R)VKLLLQVQHVSK(Q)	95%	3.9013708	0.5769867	1,390.88	3	9.604	36	47
(R)VKLLLQVQHVSK(Q)	95%	3.4998226	0.62335086	1,390.87	3	2.237	36	47

(R)VKLLLQVQHVSK(Q)	95%	3.7518728	0.6857377	1,390.87	3	-0.06489	36	47
(R)VKLLLQVQHVSKQIAVDK(Q)	95%	0.8036435	0.45463	2,045.25	4	8.544	36	53
(R)VKLLLQVQHVSKQIAVDK(Q)	95%	0.92126507	0.62703127	2,045.26	3	9.594	36	53
(R)VKLLLQVQHVSKQIAVDK(Q)	95%	0.8807414	0.6660496	2,045.26	4	9.379	36	53
(R)VKLLLQVQHVSKQIAVDK(Q)	95%	0.40054134	0.8302498	2,045.26	4	9.498	36	53
(R)VKLLLQVQHVSKQIAVDK(Q)	95%	0.76105696	0.7009821	2,045.25	4	9.021	36	53
(R)VKLLLQVQHVSKQIAVDK(Q)	95%	1.1490124	0.65760213	2,045.22	4	-6.967	36	53
(R)VKLLLQVQHVSKQIAVDK(Q)	95%	0.70557165	0.9609883	2,045.26	4	9.737	36	53
(R)VKLLLQVQHVSKQIAVDK(Q)	95%	0.9497886	1	2,045.25	4	9.021	36	53
(R)YFPTQALNFAFK(D)	95%	2.8201163	0.48946512	1,445.74	2	0.5759	85	96
(R)YFPTQALNFAFK(D)	95%	2.6086118	0.5033696	1,445.74	2	2.432	85	96
(R)YFPTQALNFAFK(D)	95%	2.7542555	0.50936455	1,445.74	2	1.251	85	96
(R)YFPTQALNFAFK(D)	95%	3.225715	0.49907044	1,445.73	2	-0.4366	85	96
(R)YFPTQALNFAFK(D)	95%	3.4541345	0.48623183	1,445.74	2	1.757	85	96
(R)YFPTQALNFAFK(D)	95%	3.337242	0.5191894	1,445.74	2	3.782	85	96
(R)YFPTQALNFAFK(D)	95%	3.3848813	0.5071025	1,445.74	2	2.854	85	96
(R)YFPTQALNFAFK(D)	95%	3.150586	0.568909	1,445.74	2	1.335	85	96
(R)YFPTQALNFAFK(D)	95%	3.60827	0.5662611	1,445.74	2	1.335	85	96
(R)YFPTQALNFAFK(D)	95%	3.2332635	0.6202611	1,445.73	2	-1.449	85	96
(R)YFPTQALNFAFK(D)	95%	3.4156973	0.60110724	1,445.73	2	-0.1834	85	96
(R)YFPTQALNFAFK(D)	95%	3.6885333	0.58319676	1,445.74	2	1.757	85	96
(R)YFPTQALNFAFK(D)	95%	3.7916903	0.57486385	1,445.74	2	1.926	85	96
(R)YFPTQALNFAFK(D)	95%	3.7431715	0.6034738	1,445.74	2	0.8291	85	96
(R)YFPTQALNFAFK(D)	95%	3.301192	0.64950514	1,445.74	2	2.01	85	96
(R)YFPTQALNFAFK(D)	95%	4.1681695	0.5691759	1,445.74	2	1.504	85	96
(R)YFPTQALNFAFK(D)	95%	3.891076	0.5938897	1,445.74	2	1.504	85	96

c denotes Cys alkylation by iodoacetamide during trypsin digestion
m denotes Met oxidation
* As determined by Peptide Profit Algorithm embedded within Scaffold v 3.6.1

ArAAC peptides identified by LC-MS/MS on the LTQ Orbtrap Velos from gel bands of the *Artemia franciscana* purified mitochondria pellet fraction, as described in Materials and Methods

Sequence	Prob*	SEQUEST XCorr	SEQUEST deltaCn	Actual Mass	Charge	Delta PPM	Start	Stop
(R)AAYFGFYDTVR(G)	95%	2.1370268	0.39900824	1,308.62	2	1.581	193	203
(R)AAYFGFYDTVR(G)	95%	2.2858422	0.47185743	1,308.62	2	5.403	193	203
(R)AAYFGFYDTVR(G)	95%	2.6155977	0.44864914	1,308.62	2	1.767	193	203
(R)AAYFGFYDTVR(G)	95%	2.3381364	0.48354843	1,308.62	2	2.233	193	203
(R)AAYFGFYDTVR(G)	95%	2.7012398	0.4803028	1,308.62	2	2.233	193	203
(R)AAYFGFYDTVR(G)	95%	2.3985193	0.52267396	1,308.62	2	3.632	193	203
(R)AAYFGFYDTVR(G)	95%	2.7308807	0.5345037	1,308.62	2	4.937	193	203
(R)AAYFGFYDTVR(G)	95%	2.6654744	0.55798995	1,308.62	2	2.233	193	203
(R)AKGDMmYK(G)	95%	1.662502	0.3644897	958.4281	2	2.803	247	254
(R)AKGDmMYK(G)	95%	1.5910258	0.38777354	958.4262	2	0.8945	247	254
(R)AKGDmMYK(G)	95%	1.5076816	0.45353422	958.4286	2	3.376	247	254
(R)AKGDmMYK(G)	95%	1.9905857	0.4013709	958.427	2	1.658	247	254
(R)AKGDMMYK(G)	95%	1.8867285	0.4621917	942.4303	2	-0.1456	247	254
(R)AKGDMMYK(G)	95%	2.1473274	0.42375162	942.4308	2	0.3719	247	254
(R)AKGDMMYK(G)	95%	1.7335829	0.51826364	942.4336	2	3.283	247	254
(R)AKGDmMYK(G)	95%	1.9368025	0.49802142	958.4277	2	2.421	247	254
(R)AKGDmMYK(G)	95%	1.9590964	0.5594227	958.4254	2	0.06754	247	254
(R)AKGDmMYK(G)	95%	2.0032697	0.6025577	958.4261	2	0.7673	247	254
(R)AKGDMmYK(G)	95%	2.0740533	0.59760547	958.4258	2	0.3856	247	254
(K)DFMAGGISAAVSK(T)	95%	1.8763276	0.32971606	1,252.61	2	0.9805	15	27
(K)DFMAGGISAAVSK(T)	95%	1.9523928	0.30912316	1,252.62	2	4.583	15	27
(K)DFMAGGISAAVSK(T)	95%	2.3350546	0.3243467	1,252.61	2	1.273	15	27
(K)DFmAGGISAAVSK(T)	95%	2.2915282	0.57816434	1,268.61	1	0.9937	15	27
(K)DFMAGGISAAVSK(T)	95%	2.9484818	0.50086373	1,252.61	1	1.527	15	27
(K)DFmAGGISAAVSK(T)	95%	2.4518595	0.56994253	1,268.61	1	3.686	15	27
(K)DFMAGGISAAVSK(T)	95%	2.9307647	0.5517602	1,252.62	1	4.545	15	27
(K)DFMAGGISAAVSK(T)	95%	2.4731262	0.4251667	1,252.62	2	2.246	15	27

(K)DFMAGGISAAVSK(T)	95%	2.3407416	0.48214042	1,252.61	2	0.9805	15	27
(K)DFmAGGISAAVSK(T)	95%	3.1587133	0.62874216	1,268.61	1	1.378	15	27
(K)DFmAGGISAAVSK(T)	95%	3.3566976	0.50369537	1,268.61	2	1.993	15	27
(K)DFMAGGISAAVSK(T)	95%	3.708786	0.5489481	1,252.62	2	4.194	15	27
(K)DFMAGGISAAVSK(T)	95%	3.7568662	0.54647917	1,252.61	2	1.467	15	27
(K)DFMAGGISAAVSK(T)	95%	3.7118073	0.5546191	1,252.62	2	4.681	15	27
(K)DFMAGGISAAVSK(T)	95%	3.3879836	0.6010476	1,252.62	2	4.681	15	27
(K)DFmAGGISAAVSK(T)	95%	3.456355	0.6062018	1,268.61	2	3.435	15	27
(K)DFmAGGISAAVSK(T)	95%	3.7784045	0.5859484	1,268.61	2	3.146	15	27
(K)DFmAGGISAAVSK(T)	95%	3.9930189	0.58327764	1,268.61	2	1.8	15	27
(K)DFmAGGISAAVSK(T)	95%	4.1232047	0.5745562	1,268.61	2	4.492	15	27
(K)DFMAGGISAAVSK(T)	95%	4.3825126	0.55741715	1,252.61	2	2.052	15	27
(K)DFMAGGISAAVSK(T)	95%	4.2479177	0.5858264	1,252.62	2	4.291	15	27
(K)DFmAGGISAAVSK(T)	95%	4.0095453	0.6168837	1,268.61	2	1.704	15	27
(K)DFmAGGISAAVSK(T)	95%	4.0444345	0.6163444	1,268.61	2	1.224	15	27
(K)DFMAGGISAAVSK(T)	95%	4.1871886	0.6079693	1,252.61	2	2.052	15	27
(K)DFmAGGISAAVSK(T)	95%	3.9829338	0.63099384	1,268.60	2	-3.392	15	27
(K)DFmAGGISAAVSK(T)	95%	4.1196337	0.619259	1,268.60	2	-3.103	15	27
(K)DFmAGGISAAVSK(T)	95%	4.0220265	0.64488846	1,268.61	2	-1.469	15	27
(K)DFMAGGISAAVSK(T)	95%	4.240115	0.63085735	1,252.62	2	2.344	15	27
(K)DFMAGGISAAVSK(T)	95%	4.4659686	0.61639094	1,252.61	2	2.052	15	27
(K)DFmAGGISAAVSK(T)	95%	4.189992	0.6386473	1,268.61	2	0.7428	15	27
(K)DFmAGGISAAVSK(T)	95%	3.843778	0.6705383	1,268.61	2	1.993	15	27
(K)DFmAGGISAAVSK(T)	95%	4.280241	0.6389991	1,268.61	2	2.762	15	27
(K)DFmAGGISAAVSK(T)	95%	4.06334	0.6575932	1,268.60	2	-2.238	15	27
(K)DFmAGGISAAVSK(T)	95%	4.1355853	0.6528297	1,268.61	2	2.57	15	27
(K)DFmAGGISAAVSK(T)	95%	4.532929	0.6228564	1,268.60	2	-2.815	15	27
(K)DFmAGGISAAVSK(T)	95%	4.3063407	0.6425778	1,268.60	2	-1.853	15	27
(K)DFmAGGISAAVSK(T)	95%	4.9966097	0.6394864	1,268.61	2	0.9351	15	27
(K)DFmAGGISAAVSK(T)	95%	4.5066066	0.68266165	1,268.60	2	-2.046	15	27
(K)DVFKQIFmSGVDKK(T)	95%	2.7772472	0.39039108	1,656.86	3	1.051	97	110
(K)DVFKQIFMSGVDKK(T)	95%	3.3500333	0.43561304	1,640.86	3	0.7896	97	110

(R)EFSGLGNcLVK(I)	95%	2.3043942	0.25349402	1,222.60	1	1.195	157	167
(R)EFSGLGNcLVK(I)	95%	1.769603	0.2867627	1,222.61	2	2.83	157	167
(R)EFSGLGNcLVK(I)	95%	1.973433	0.25177735	1,222.61	2	4.127	157	167
(R)EFSGLGNcLVK(I)	95%	2.179437	0.24787192	1,222.60	2	2.431	157	167
(R)EFSGLGNcLVK(I)	95%	1.8761427	0.33639878	1,222.60	1	2.592	157	167
(R)EFSGLGNcLVK(I)	95%	2.0888026	0.29341504	1,222.60	2	2.032	157	167
(R)EFSGLGNcLVK(I)	95%	2.1778834	0.37407213	1,222.61	1	4.986	157	167
(R)EFSGLGNcLVK(I)	95%	2.1235266	0.3177052	1,222.61	2	2.631	157	167
(R)EFSGLGNcLVK(I)	95%	2.21024	0.3006864	1,222.61	2	3.03	157	167
(R)EFSGLGNcLVK(I)	95%	2.2232416	0.30954507	1,222.61	2	3.928	157	167
(R)EFSGLGNcLVK(I)	95%	2.2623522	0.31903675	1,222.61	2	3.329	157	167
(R)EFSGLGNcLVK(I)	95%	2.195453	0.35196847	1,222.61	2	3.429	157	167
(R)EFSGLGNcLVK(I)	95%	2.3035815	0.37891838	1,222.61	2	2.73	157	167
(R)EFSGLGNcLVK(I)	95%	2.1211221	0.42312488	1,222.60	2	2.132	157	167
(R)EFSGLGNcLVK(I)	95%	2.619608	0.35353643	1,222.60	2	2.431	157	167
(R)EFSGLGNcLVK(I)	95%	2.6687667	0.35119054	1,222.61	2	4.526	157	167
(R)EFSGLGNcLVK(I)	95%	2.4826813	0.37334645	1,222.61	2	3.229	157	167
(R)EFSGLGNcLVK(I)	95%	2.5126684	0.40762645	1,222.60	2	1.733	157	167
(R)EFSGLGNcLVK(I)	95%	2.6800644	0.39283636	1,222.61	2	3.529	157	167
(R)EFSGLGNcLVK(I)	95%	2.8527653	0.39546868	1,222.61	2	4.526	157	167
(R)EFSGLGNcLVK(I)	95%	2.5335135	0.44290036	1,222.60	2	2.032	157	167
(R)EFSGLGNcLVK(I)	95%	2.5491369	0.4434005	1,222.60	2	2.232	157	167
(R)EFSGLGNcLVK(I)	95%	2.7156026	0.43734035	1,222.60	2	1.533	157	167
(R)EFSGLGNcLVK(I)	95%	2.8955097	0.4124557	1,222.60	2	2.132	157	167
(R)EFSGLGNcLVK(I)	95%	2.473862	0.47625124	1,222.60	2	2.132	157	167
(R)EFSGLGNcLVK(I)	95%	2.7330139	0.47154102	1,222.60	2	0.7352	157	167
(K)EQGVLSFWR(G)	95%	2.1191726	0.11336668	1,120.57	1	3.38	68	76
(K)GAAEREFSGLGNcLVK(I)	95%	2.9936745	0.2913408	1,706.85	2	6.019	152	167
(K)GAAEREFSGLGNcLVK(I)	95%	2.6276677	0.31922302	1,706.85	2	4.303	152	167
(K)GAAEREFSGLGNcLVK(I)	95%	2.6534796	0.26842478	1,706.84	3	1.972	152	167
(K)GAAEREFSGLGNcLVK(I)	95%	2.3140447	0.3071723	1,706.85	3	4.224	152	167
(K)GAAEREFSGLGNcLVK(I)	95%	3.512129	0.35014325	1,706.84	2	2.016	152	167

(K)GAAEREFSGLGNcLVK(I)	95%	3.063011	0.40237036	1,706.85	2	2.874	152	167
(K)GAAEREFSGLGNcLVK(I)	95%	2.7459204	0.37829602	1,706.84	3	1.436	152	167
(K)GAAEREFSGLGNcLVK(I)	95%	3.0604439	0.4893606	1,706.84	3	1.115	152	167
(K)GAAEREFSGLGNcLVK(I)	95%	3.3881116	0.47780395	1,706.85	3	4.438	152	167
(K)GALSNVFR(G)	95%	1.8292701	0.23741466	862.4682	2	2.437	275	282
(K)GALSNVFR(G)	95%	1.772857	0.27306044	862.4694	2	3.851	275	282
(K)GALSNVFR(G)	95%	3.0495768	0.14147235	862.4663	2	0.3166	275	282
(K)GALSNVFR(G)	95%	2.2474809	0.22862542	862.4672	2	1.306	275	282
(K)GALSNVFR(G)	95%	2.1977525	0.21120857	862.469	2	3.427	275	282
(K)GALSNVFR(G)	95%	2.6134791	0.15818512	862.4693	2	3.78	275	282
(K)GALSNVFR(G)	95%	2.4762938	0.24398148	862.4677	2	1.942	275	282
(K)GALSNVFR(G)	95%	2.652611	0.24531944	862.4674	2	1.589	275	282
(K)GALSNVFR(G)	95%	2.7882535	0.2304667	862.4701	2	4.699	275	282
(K)GALSNVFR(G)	95%	2.7163706	0.24157645	862.4677	2	1.872	275	282
(K)GALSNVFR(G)	95%	2.381309	0.28647086	862.4692	2	3.568	275	282
(K)GALSNVFR(G)	95%	2.74967	0.2370778	862.4688	2	3.144	275	282
(K)GALSNVFR(G)	95%	2.8456547	0.24478027	862.4683	2	2.579	275	282
(K)GALSNVFR(G)	95%	2.9140115	0.26327828	862.4668	2	0.8821	275	282
(K)GALSNVFR(G)	95%	3.004509	0.24132554	862.4674	2	1.589	275	282
(K)GALSNVFR(G)	95%	2.5297189	0.29859227	862.4688	2	3.215	275	282
(K)GALSNVFR(G)	95%	2.9208057	0.2768232	862.467	2	1.094	275	282
(K)GALSNVFR(G)	95%	2.5894063	0.3002715	862.4692	2	3.639	275	282
(K)GALSNVFR(G)	95%	2.734149	0.2743962	862.4688	2	3.144	275	282
(K)GALSNVFR(G)	95%	3.02431	0.24921623	862.4684	2	2.649	275	282
(K)GALSNVFR(G)	95%	2.9465072	0.26540977	862.4687	2	3.073	275	282
(K)GDmMYK(G)	95%	1.5406417	0.43748587	759.2939	1	0.8516	249	254
(K)GDMmYK(G)	95%	1.0954076	0.60577106	759.2944	1	1.494	249	254
(K)GDMmYK(G)	95%	1.3820013	0.6387976	759.2948	2	2.038	249	254
(K)GDmMYK(G)	95%	1.7895014	0.5456015	759.295	2	2.199	249	254
(K)GDmMYK(G)	95%	1.906612	0.5268315	759.2946	2	1.717	249	254
(K)GDMmYK(G)	95%	1.6436832	0.69012564	759.2946	2	1.717	249	254
(K)GDmmYKGTLDcWAK(I)	95%	1.9841977	0.33570033	1,706.72	2	6.779	249	262

(K)GDmmYKGTLDcWAK(I)	95%	1.9645487	0.3084779	1,706.72	2	5.778	249	262
(K)GDmmYKGTLDcWAK(I)	95%	2.1364875	0.35261774	1,706.72	2	6.064	249	262
(K)GDmmYKGTLDcWAK(I)	95%	1.772044	0.3405563	1,706.72	2	3.491	249	262
(K)GDmmYKGTLDcWAK(I)	95%	2.2483532	0.31746516	1,706.72	2	5.421	249	262
(K)GDmmYKGTLDcWAK(I)	95%	2.1748834	0.3422772	1,706.72	2	5.778	249	262
(K)GDmmYKGTLDcWAK(I)	95%	2.2191687	0.38705936	1,706.72	2	7.923	249	262
(K)GDmmYKGTLDcWAK(I)	95%	2.3003848	0.4227083	1,706.72	2	6.35	249	262
(K)GDmmYKGTLDcWAK(I)	95%	2.397353	0.43364865	1,706.72	2	7.065	249	262
(K)GDmmYKGTLDcWAK(I)	95%	2.3749466	0.48153585	1,706.73	2	8.995	249	262
(K)GDmmYKGTLDcWAK(I)	95%	2.414114	0.5180476	1,706.72	2	6.493	249	262
(K)GDmmYKGTLDcWAK(I)	95%	2.3230174	0.49548587	1,706.72	2	5.421	249	262
(K)GDmmYKGTLDcWAK(I)	95%	2.5755682	0.46959987	1,706.72	2	5.564	249	262
(K)GIIDcFVR(I)	95%	2.0138838	0.21124661	978.4979	2	2.106	57	64
(K)GIIDcFVR(I)	95%	1.938655	0.24047329	978.4971	2	1.296	57	64
(K)GIIDcFVR(I)	95%	1.9371768	0.2793106	978.4962	2	0.3617	57	64
(K)GIIDcFVR(I)	95%	1.7684301	0.37349957	978.4965	2	0.6732	57	64
(K)GIIDcFVR(I)	95%	1.9237759	0.37517408	978.501	2	5.222	57	64
(K)GIIDcFVR(I)	95%	2.1374862	0.3793017	978.5007	2	4.973	57	64
(K)GIIDcFVR(I)	95%	2.1154277	0.38634923	978.5009	2	5.16	57	64
(K)GIIDcFVR(I)	95%	2.1947098	0.38325647	978.4969	2	1.047	57	64
(K)GIIDcFVR(I)	95%	2.1679337	0.38598508	978.4994	2	3.602	57	64
(K)GIIDcFVR(I)	95%	1.8642646	0.46378067	978.4977	2	1.919	57	64
(K)GIIDcFVR(I)	95%	2.258576	0.4031844	978.4968	2	0.9848	57	64
(K)GIIDcFVR(I)	95%	2.2556872	0.39940548	978.5006	2	4.848	57	64
(K)GIIDcFVR(I)	95%	2.235617	0.41388467	978.4975	2	1.67	57	64
(K)GIIDcFVR(I)	95%	2.6266332	0.3415809	978.498	2	2.169	57	64
(K)GIIDcFVR(I)	95%	2.0067189	0.44226563	978.4988	2	3.041	57	64
(K)GIIDcFVR(I)	95%	2.2514029	0.41120481	978.4981	2	2.356	57	64
(K)GIIDcFVR(I)	95%	2.3929076	0.40900686	978.497	2	1.172	57	64
(K)GIIDcFVR(I)	95%	2.372154	0.38926396	978.4989	2	3.103	57	64
(K)GIIDcFVR(I)	95%	1.8081877	0.5089011	978.4986	2	2.792	57	64
(K)GIIDcFVR(I)	95%	2.2260408	0.47662508	978.4987	2	2.916	57	64

(R)GTGGALVLVFYDELK(V)	95%	3.2250285	0.51320547	1,580.85	2	4.81	283	297
(K)GTLDcWAK(I)	95%	1.9241146	0.34048036	949.4325	1	-0.4766	255	262
(K)GTLDcWAK(I)	95%	1.8695213	0.31884605	949.4346	1	1.771	255	262
(K)GTLDcWAK(I)	95%	1.8207401	0.34722832	949.4345	1	1.643	255	262
(K)GTLDcWAK(I)	95%	1.3970182	0.33394805	949.4358	1	3.055	255	262
(K)GTLDcWAK(I)	95%	1.7331655	0.3134504	949.4357	1	2.927	255	262
(K)GTLDcWAK(I)	95%	1.7870952	0.3737616	949.4342	2	1.372	255	262
(K)GTLDcWAK(I)	95%	1.9978639	0.35616523	949.4334	2	0.4725	255	262
(K)GTLDcWAK(I)	95%	1.8940556	0.3464941	949.4361	2	3.362	255	262
(K)GTLDcWAK(I)	95%	1.9563673	0.43260893	949.4331	2	0.1514	255	262
(K)GTLDcWAK(I)	95%	1.934305	0.4971748	949.4303	2	-2.803	255	262
(K)GTLDcWAK(I)	95%	2.1246314	0.49439204	949.4301	2	-2.931	255	262
(K)GTLDcWAK(I)	95%	1.9686269	0.5367341	949.4307	2	-2.353	255	262
(K)GTLDcWAK(I)	95%	2.1342936	0.4973944	949.4325	2	-0.4266	255	262
(K)GTLDcWAK(I)	95%	2.0604186	0.5302006	949.4316	2	-1.454	255	262
(K)GTLDcWAK(I)	95%	2.3300173	0.5017376	949.4325	2	-0.4908	255	262
(K)GTLDcWAK(I)	95%	2.0632975	0.5697961	949.4338	2	0.8578	255	262
(K)GTLDcWAK(I)	95%	2.277217	0.54968524	949.4342	2	1.307	255	262
(K)GTLDcWAK(I)	95%	2.169422	0.5751339	949.4341	2	1.179	255	262
(K)GTLDcWAK(I)	95%	2.4117815	0.6233497	949.4343	2	1.436	255	262
(K)IFKSDGLTGLYR(G)	95%	1.5556957	0.32962245	1,368.74	3	2.323	168	179
(K)IFKSDGLTGLYR(G)	95%	2.4002752	0.41989553	1,368.75	3	3.86	168	179
(K)IFKSDGLTGLYR(G)	95%	3.6448846	0.53213453	1,368.74	2	3.268	168	179
(K)IFKSDGLTGLYR(G)	95%	3.3275843	0.5701224	1,368.74	2	2.912	168	179
(K)IFKSDGLTGLYR(G)	95%	3.2100449	0.5601406	1,368.74	3	1.521	168	179
(R)IPKEQGVLSFWR(G)	95%	3.3035588	0.08124945	1,458.81	2	4.576	65	76
(R)IPKEQGVLSFWR(G)	95%	2.4447412	0.14107575	1,458.80	3	4.148	65	76
(R)IPKEQGVLSFWR(G)	95%	3.1246552	0.10282941	1,458.80	3	2.392	65	76
(R)IPKEQGVLSFWR(G)	95%	3.7823527	0.05516165	1,458.80	2	3.907	65	76
(R)IPKEQGVLSFWR(G)	95%	2.920827	0.15712707	1,458.80	3	1.452	65	76
(R)IPKEQGVLSFWR(G)	95%	3.151161	0.16300704	1,458.80	3	0.3226	65	76
(R)IPKEQGVLSFWR(G)	95%	3.2114928	0.18610099	1,458.81	3	4.587	65	76

(R)IPKEQGVLSFWR(G)	95%	2.7810705	0.24215478	1,458.80	3	1.138	65	76
(K)IYKSEGGGAFFK(G)	95%	2.0957077	0.18398613	1,302.66	3	0.984	263	274
(K)IYKSEGGGAFFK(G)	95%	2.2347488	0.30807146	1,302.66	2	1.041	263	274
(K)IYKSEGGGAFFK(G)	95%	2.5116973	0.2120539	1,302.66	3	2.037	263	274
(K)KQADPLSFLK(D)	95%	2.5251694	0.36569667	1,145.65	1	1.731	5	14
(K)KQADPLSFLK(D)	95%	2.6284776	0.27908492	1,145.64	2	-0.4633	5	14
(K)KQADPLSFLK(D)	95%	2.365125	0.323372	1,145.65	2	2.624	5	14
(K)KQADPLSFLK(D)	95%	2.4744878	0.3585689	1,145.65	2	0.4948	5	14
(K)KQADPLSFLK(D)	95%	2.5526931	0.32355857	1,145.65	2	2.518	5	14
(K)KQADPLSFLK(D)	95%	2.176507	0.48383492	1,145.65	2	1.347	5	14
(K)KQADPLSFLK(D)	95%	2.6373646	0.39878726	1,145.65	2	3.05	5	14
(K)KQADPLSFLK(D)	95%	2.59199	0.45494607	1,145.65	2	3.369	5	14
(K)KQADPLSFLK(D)	95%	2.7338874	0.4540196	1,145.65	2	2.198	5	14
(K)KQADPLSFLK(D)	95%	2.8427708	0.44885656	1,145.65	2	2.305	5	14
(K)KQADPLSFLK(D)	95%	2.6542485	0.48408473	1,145.65	2	4.221	5	14
(K)KQADPLSFLK(D)	95%	2.8155074	0.4566467	1,145.65	2	3.369	5	14
(K)KQADPLSFLK(D)	95%	2.7900116	0.48689392	1,145.65	2	0.9207	5	14
(K)KQADPLSFLK(D)	95%	2.7370095	0.5105253	1,145.65	2	1.559	5	14
(K)KQADPLSFLK(D)	95%	2.9773622	0.56345874	1,145.65	2	0.8142	5	14
(K)KQADPLSFLKDFmAGGISAAVSK(T)	95%	2.939341	0.38632742	2,396.25	3	5.299	5	27
(K)KQADPLSFLKDFmAGGISAAVSK(T)	95%	4.1735487	0.58555603	2,396.25	3	4.765	5	27
(R)LAADTGKGAAER(E)	95%	2.1768897	0.31045118	1,158.60	2	0.3081	145	156
(R)LAADTGKGAAER(E)	95%	1.6127443	0.29645368	1,158.60	3	1.718	145	156
(R)LAADTGKGAAER(E)	95%	2.5259275	0.41619775	1,158.60	2	0.3081	145	156
(R)LAADTGKGAAER(E)	95%	3.0669043	0.43513188	1,158.60	2	0.6239	145	156
(R)LAADTGKGAAER(E)	95%	3.2191055	0.46986222	1,158.60	2	0.2028	145	156
(R)LAADTGKGAAER(E)	95%	3.4876764	0.4664684	1,158.60	2	0.8344	145	156
(R)LAADTGKGAAEREFSGLGNcLVK(I)	95%	2.5675778	0.32260853	2,363.19	3	0.5416	145	167
(R)LAADTGKGAAEREFSGLGNcLVK(I)	95%	2.542102	0.44614488	2,363.20	3	2.246	145	167
(K)LLLQVQHVSK(Q)	95%	1.5901186	0.27691534	1,163.70	3	0.008685	38	47
(K)LLLQVQHVSK(Q)	95%	2.5334406	0.41808575	1,163.70	2	-0.4514	38	47
(K)LLLQVQHVSK(Q)	95%	1.5087273	0.4015147	1,163.71	3	2.917	38	47

(K)LLLQVQHVSK(Q)	95%	2.8333876	0.40771532	1,163.70	2	1.33	38	47
(K)LLLQVQHVSK(Q)	95%	3.0532882	0.39996722	1,163.70	2	0.2822	38	47
(K)LLLQVQHVSK(Q)	95%	2.6157255	0.47296336	1,163.70	2	1.121	38	47
(K)LLLQVQHVSK(Q)	95%	2.8193011	0.4561086	1,163.70	2	0.1774	38	47
(K)LLLQVQHVSK(Q)	95%	2.9905267	0.44899842	1,163.70	2	-1.814	38	47
(K)LLLQVQHVSK(Q)	95%	2.9936495	0.469428	1,163.70	2	-0.5562	38	47
(K)LLLQVQHVSK(Q)	95%	2.9755974	0.45396397	1,163.70	2	1.435	38	47
(K)LLLQVQHVSK(Q)	95%	3.0123935	0.4750409	1,163.70	2	-0.7658	38	47
(K)LLLQVQHVSK(Q)	95%	3.0465937	0.49051106	1,163.70	2	-1.919	38	47
(K)LLLQVQHVSK(Q)	95%	3.2986517	0.46121264	1,163.70	2	-3.176	38	47
(K)LLLQVQHVSK(Q)	95%	3.2875726	0.4638762	1,163.70	2	-2.652	38	47
(K)LLLQVQHVSK(Q)	95%	3.4618406	0.44759285	1,163.70	2	-2.338	38	47
(K)LLLQVQHVSK(Q)	95%	3.1676018	0.44910446	1,163.70	2	1.54	38	47
(K)LLLQVQHVSK(Q)	95%	2.8573897	0.49653038	1,163.70	2	0.7015	38	47
(K)LLLQVQHVSK(Q)	95%	3.0011368	0.47840476	1,163.70	2	1.645	38	47
(K)LLLQVQHVSK(Q)	95%	3.249717	0.4794547	1,163.70	2	1.435	38	47
(K)LLLQVQHVSK(Q)	95%	3.308077	0.4753742	1,163.70	2	-0.3466	38	47
(K)LLLQVQHVSK(Q)	95%	3.0594735	0.5288851	1,163.70	2	1.435	38	47
(K)LLLQVQHVSK(Q)	95%	3.2971466	0.5069682	1,163.70	2	1.435	38	47
(K)LLLQVQHVSK(Q)	95%	3.1957507	0.52902335	1,163.70	2	1.435	38	47
(R)MMmQSGR(A)	95%	1.8776658	0.3160126	855.3407	2	0.5687	240	246
(R)MMMQSGR(A)	95%	2.1152053	0.2942393	839.3465	2	1.428	240	246
(R)MmMQSGR(A)	95%	1.6528468	0.4599823	855.3416	2	1.709	240	246
(R)MmMQSGR(A)	95%	2.297251	0.3467267	855.3404	2	0.2836	240	246
(R)mMMQSGR(A)	95%	2.0446062	0.46987337	855.3405	2	0.3548	240	246
(K)QADPLSFLK(D)	95%	1.9259033	0.25262633	1,017.55	2	1.605	6	14
(K)QADPLSFLK(D)	95%	1.8893678	0.30845997	1,017.55	2	2.563	6	14
(K)QADPLSFLK(D)	95%	2.276983	0.25217527	1,017.55	2	2.204	6	14
(K)QADPLSFLK(D)	95%	2.0505404	0.30586988	1,017.55	2	4.061	6	14
(K)QADPLSFLK(D)	95%	2.159403	0.28834817	1,017.55	2	2.443	6	14
(K)QADPLSFLK(D)	95%	2.2055247	0.398548	1,017.55	2	0.9454	6	14
(K)QADPLSFLKDFmAGGISAAVSK(T)	95%	2.6304297	0.44374156	2,268.16	3	5.477	6	27

(K)QADPLSFLKDFmAGGISAAVSK(T)	95%	2.7990313	0.44209725	2,268.16	3	4.105	6	27
(K)QIAVDK(Q)	95%	1.8714768	0.18828385	672.3828	1	3.014	48	53
(K)QIAVDKQYK(G)	95%	2.035889	0.29010987	1,091.60	2	2.645	48	56
(K)QIAVDKQYK(G)	95%	1.8088694	0.44093454	1,091.60	2	-1.154	48	56
(K)QIFmSGVDK(K)	95%	1.7043972	0.3412273	1,039.50	2	3.486	101	109
(K)QIFmSGVDK(K)	95%	1.9083537	0.35683703	1,039.50	2	1.492	101	109
(K)QIFMSGVDK(K)	95%	1.7102792	0.45887077	1,023.51	2	0.722	101	109
(K)QIFMSGVDKK(T)	95%	2.0790358	0.17194237	1,151.61	3	3.808	101	110
(K)QIFmSGVDKK(T)	95%	2.1314793	0.26430297	1,167.60	2	2.348	101	110
(K)QIFmSGVDKK(T)	95%	1.3911556	0.48816255	1,167.60	2	-0.786	101	110
(K)QIFmSGVDKK(T)	95%	1.8350586	0.34449223	1,167.60	2	2.87	101	110
(K)QIFmSGVDKK(T)	95%	1.4492801	0.31891558	1,167.60	3	2.571	101	110
(K)QIFmSGVDKK(T)	95%	1.7692106	0.28300023	1,167.60	3	1.631	101	110
(K)QIFmSGVDKK(T)	95%	1.8252232	0.4571634	1,167.60	2	0.04969	101	110
(K)SDGLTGLYR(G)	95%	1.5281078	0.29034853	980.4963	1	3.52	171	179
(K)SDGLTGLYR(G)	95%	1.6348329	0.45334458	980.4952	1	2.339	171	179
(K)SDGLTGLYR(G)	95%	1.7619443	0.39951956	980.4963	1	3.458	171	179
(K)SDGLTGLYR(G)	95%	2.2174218	0.36942285	980.4963	2	3.444	171	179
(K)SDGLTGLYR(G)	95%	2.782688	0.3189151	980.4942	2	1.33	171	179
(K)SDGLTGLYR(G)	95%	2.5241535	0.35022038	980.4951	2	2.263	171	179
(K)SDGLTGLYR(G)	95%	2.4595993	0.3969949	980.4947	2	1.827	171	179
(K)SDGLTGLYR(G)	95%	2.497673	0.39149997	980.4949	2	2.076	171	179
(K)SDGLTGLYR(G)	95%	2.6007926	0.36362606	980.4961	2	3.32	171	179
(K)SDGLTGLYR(G)	95%	2.5929601	0.39574736	980.4939	2	1.019	171	179
(K)SDGLTGLYR(G)	95%	2.8433194	0.34474623	980.4964	2	3.569	171	179
(K)SDGLTGLYR(G)	95%	2.7061496	0.4093908	980.4941	2	1.206	171	179
(K)SDGLTGLYR(G)	95%	2.5923047	0.43580195	980.4942	2	1.392	171	179
(K)SDGLTGLYR(G)	95%	2.720302	0.4220965	980.4961	2	3.32	171	179
(K)SDGLTGLYR(G)	95%	2.514751	0.483121	980.4941	2	1.206	171	179
(K)SDGLTGLYR(G)	95%	2.7036452	0.44945183	980.4942	2	1.33	171	179
(K)SDGLTGLYR(G)	95%	2.659927	0.4782238	980.4952	2	2.325	171	179
(K)SDGLTGLYR(G)	95%	3.010467	0.45124203	980.4938	2	0.9569	171	179

(K)SDGLTGLYR(G)	95%	3.1681356	0.44586194	980.4941	2	1.268	171	179
(K)SDGLTGLYR(G)	95%	2.8709862	0.4923846	980.4942	2	1.392	171	179
(K)SDGLTGLYR(G)	95%	2.9480307	0.4867455	980.4945	2	1.641	171	179
(K)SDGLTGLYR(G)	95%	3.1234145	0.44587952	980.4967	2	3.942	171	179
(K)SEGGGAFFK(G)	95%	1.5117924	0.20394787	898.4217	1	3.525	266	274
(K)SEGGGAFFK(G)	95%	2.021586	0.2834818	898.4186	2	0.04878	266	274
(K)SEGGGAFFK(G)	95%	2.2303674	0.20297597	898.421	2	2.695	266	274
(K)SEGGGAFFK(G)	95%	1.9288663	0.3431674	898.4189	2	0.3202	266	274
(K)SEGGGAFFK(G)	95%	2.2609901	0.30755046	898.4182	2	-0.3584	266	274
(K)SEGGGAFFK(G)	95%	2.1045525	0.31523016	898.4219	2	3.713	266	274
(K)SEGGGAFFK(G)	95%	2.1561081	0.3105195	898.4211	2	2.763	266	274
(K)SEGGGAFFK(G)	95%	2.0882883	0.35941148	898.4203	2	1.949	266	274
(K)SEGGGAFFK(G)	95%	2.8417013	0.27455518	898.4214	2	3.102	266	274
(K)SEGGGAFFK(G)	95%	2.6736033	0.375558	898.4191	2	0.5917	266	274
(K)SEGGGAFFK(G)	95%	2.7586138	0.33202204	898.4214	2	3.102	266	274
(K)SEGGGAFFK(G)	95%	2.347198	0.42620972	898.422	2	3.849	266	274
(K)SEGGGAFFK(G)	95%	2.8542151	0.4887665	898.4192	2	0.7274	266	274
(K)TAVAPIER(V)	95%	1.9064679	0.2293666	855.483	1	1.809	28	35
(K)TAVAPIER(V)	95%	2.0489295	0.27835175	855.4806	2	-0.9859	28	35
(K)TAVAPIER(V)	95%	2.1418114	0.31706524	855.4819	1	0.455	28	35
(K)TAVAPIER(V)	95%	1.5860076	0.23378287	855.4845	1	3.519	28	35
(K)TAVAPIER(V)	95%	1.9760915	0.3269701	855.4819	2	0.5106	28	35
(K)TAVAPIER(V)	95%	1.9886847	0.2848684	855.4838	2	2.72	28	35
(K)TAVAPIER(V)	95%	1.957856	0.3232131	855.4835	2	2.363	28	35
(K)TAVAPIER(V)	95%	2.0635586	0.30505985	855.4835	2	2.363	28	35
(K)TAVAPIER(V)	95%	2.193077	0.30198327	855.4841	2	3.076	28	35
(K)TAVAPIERVK(L)	95%	1.9560317	0.29981822	1,082.64	2	-0.2242	28	37
(R)TRLAADTGK(G)	95%	1.8763515	0.3017815	931.5085	2	-0.4422	143	151
(R)VKLLLQVQHVSK(Q)	95%	3.191474	0.29478362	1,390.87	2	3.431	36	47
(R)VKLLLQVQHVSK(Q)	95%	3.0681016	0.36719126	1,390.87	2	2.028	36	47
(R)VKLLLQVQHVSK(Q)	95%	2.8656914	0.43527558	1,390.87	2	2.028	36	47
(R)VKLLLQVQHVSK(Q)	95%	2.0614307	0.39312202	1,390.86	3	-1.709	36	47

(R)VKLLLQVQHVSK(Q)	95%	3.7457674	0.39333624	1,390.87	2	-0.1649	36	47
(R)VKLLLQVQHVSK(Q)	95%	2.1953516	0.36912352	1,390.87	3	3.684	36	47
(R)VKLLLQVQHVSK(Q)	95%	3.079258	0.4859793	1,390.87	2	0.6245	36	47
(R)VKLLLQVQHVSK(Q)	95%	3.4440315	0.46202114	1,390.87	2	4.571	36	47
(R)VKLLLQVQHVSK(Q)	95%	2.1489131	0.44433296	1,390.87	3	3.158	36	47
(R)VKLLLQVQHVSK(Q)	95%	2.8041406	0.5815428	1,390.87	2	1.238	36	47
(R)VKLLLQVQHVSK(Q)	95%	2.7990618	0.43687615	1,390.87	3	0.264	36	47
(R)VKLLLQVQHVSK(Q)	95%	3.6744637	0.5117442	1,390.87	2	1.589	36	47
(R)VKLLLQVQHVSK(Q)	95%	2.2721677	0.53152883	1,390.87	3	0.7244	36	47
(R)VKLLLQVQHVSK(Q)	95%	3.5774784	0.5902899	1,390.87	2	0.1859	36	47
(R)VKLLLQVQHVSK(Q)	95%	3.3684971	0.5503672	1,390.87	3	-0.06489	36	47
(R)YFPTQALNFAFK(D)	95%	2.1074216	0.3864726	1,445.73	2	-4.065	85	96
(R)YFPTQALNFAFK(D)	95%	1.7239901	0.47510207	1,445.74	2	6.904	85	96
(R)YFPTQALNFAFK(D)	95%	2.528651	0.2983028	1,445.74	2	1.673	85	96
(R)YFPTQALNFAFK(D)	95%	2.2993588	0.42318317	1,445.74	2	2.685	85	96
(R)YFPTQALNFAFK(D)	95%	2.9470322	0.50074303	1,445.74	2	2.854	85	96
(R)YFPTQALNFAFK(D)	95%	2.8187687	0.5434221	1,445.74	2	4.71	85	96
(R)YFPTQALNFAFK(D)	95%	3.0222247	0.56371707	1,445.74	2	3.529	85	96
(R)YFPTQALNFAFK(D)	95%	3.1642904	0.5666858	1,445.74	2	2.601	85	96
(R)YFPTQALNFAFK(D)	95%	3.0205104	0.61782914	1,445.74	2	2.01	85	96
(R)YFPTQALNFAFK(D)	95%	3.4586637	0.5781355	1,445.74	2	3.445	85	96
(R)YFPTQALNFAFK(D)	95%	3.467686	0.62427616	1,445.74	2	1.082	85	96
(R)YFPTQALNFAFK(D)	95%	3.7894998	0.5942812	1,445.74	2	2.854	85	96
(R)YFPTQALNFAFK(D)	95%	3.1329002	0.66264516	1,445.74	2	2.601	85	96
(R)YFPTQALNFAFK(D)	95%	3.3295178	0.6419884	1,445.74	2	3.529	85	96
(R)YFPTQALNFAFK(D)	95%	3.4310231	0.6523597	1,445.74	2	5.807	85	96
(R)YFPTQALNFAFK(D)	95%	3.7415855	0.6256677	1,445.74	2	1.588	85	96
(R)YFPTQALNFAFK(D)	95%	3.521916	0.65729415	1,445.74	2	3.023	85	96
(R)YFPTQALNFAFK(D)	95%	3.7122674	0.64246064	1,445.74	2	1.42	85	96
(R)YFPTQALNFAFK(D)	95%	3.6500506	0.6492905	1,445.74	2	5.638	85	96
(R)YFPTQALNFAFK(D)	95%	3.4949489	0.6767713	1,445.74	2	2.432	85	96

c denotes Cys alkylation by iodoacetamide during trypsin digestion

m denotes Met oxidation
* As determined by Peptide Profit Algorithm embedded within Scaffold v 3.6.1

Peptides identified by LC-MS/MS on the Waters Q-TOF instrument from ArAAC expressed in *Saccharomyces cerevisiae* mitochondria digested by different proteases and based on various Mascot searches, as described in Materials and Methods

Sequence	Prob	Mascot ion score	Actual Mass	Charge	Delta PPM	Start	Stop
(M)ATKKQADPL(S) + [+42.0106 at N-term A]	95%	17	507.2677	2	-34.01	2	10
(M)ATKKQADPLSF(L) + [+42.0470 at N-term A]	95%	42	624.3297	2	-37.92	2	12
(M)ATKKQADPLSF(L) + [+42.0470 at N-term A]	95%	22	624.3318	2	-34.55	2	12
(M)ATKKQADPLSFL(K) + [+42.0470 at N-term A]	95%	54	680.8795	2	-23.34	2	13
(K)KQADPLSFLK(D)	95%	28	573.8314	2	3.32	5	14
(K)KQADPLSFLK(D)	95%	42	596.3179	2	-44.16	5	14
(K)KQADPLSFLKDFMAGGISAAV(S)	95%	39	722.709	3	-6.42	5	25
(K)KQADPLSFLKDFMAGGISAAVSK(T)	95%	85	794.4096	3	-16.42	5	27
(K)KQADPLSFLKDFMAGGISAAVSK(T)	95%	99	794.4114	3	-14.15	5	27
(K)KQADPLSFLKDFMAGGISAAVSK(T)	95%	22	794.414	3	-10.87	5	27
(K)KQADPLSFLKDFmAGGISAAVSK(T)	95%	83	794.4171	3	-22.26	5	27
(K)QADPLSFLK(D)	95%	21	509.7815	2	-1.02	6	14
(K)QADPLSFLK(D)	95%	45	509.7849	2	5.66	6	14
(K)QADPLSFLK(D)	95%	21	509.7869	2	9.6	6	14
(K)QADPLSFLKDFMAGGISAAVSK(T)	95%	64	751.7126	3	-15.59	6	27
(K)QADPLSFLKDFMAGGISAAVSK(T)	95%	76	751.7133	3	-14.66	6	27
(K)QADPLSFLKDFMAGGISAAVSK(T)	95%	85	751.7164	3	-10.53	6	27
(K)QADPLSFLKDFMAGGISAAVSK(T)	95%	93	751.7167	3	-10.13	6	27
(K)QADPLSFLKDFMAGGISAAVSK(T)	95%	88	751.7172	3	-9.47	6	27
(K)QADPLSFLKDFMAGGISAAVSK(T)	95%	71	751.7188	3	-7.34	6	27
(K)QADPLSFLKDFMAGGISAAVSK(T)	95%	94	751.7198	3	-6	6	27
(K)TAVAPIER(V)	95%	27	428.7527	2	11	28	35
(K)TAVAPIER(V)	95%	66	428.7547	2	15.7	28	35

(A)VAPIERVKL(L)	95%	33	512.8362	2	13.5	30	38
(A)VAPIERVKL(L)	95%	29	512.8382	2	17.4	30	38
(A)VAPIERVKLL(L)	95%	23	569.3669	2	-7.79	30	39
(K)LLLQVQHVSK(Q)	95%	30	582.8522	2	-10.99	38	47
(K)LLLQVQHVSK(Q)	95%	47	582.8526	2	-10.3	38	47
(K)GIIDCFVR(I)	95%	38	490.2563	2	2.4	57	64
(K)GIIDCFVR(I)	95%	27	490.2565	2	2.81	57	64
(K)GIIDCFVR(I)	95%	21	490.2583	2	6.49	57	64
(K)GIIDCFVR(I)	95%	39	490.2619	2	13.8	57	64
(F)VRIPKEQGVL(S)	95%	53	569.8402	2	-18.58	63	72
(F)VRIPKEQGVL(S)	95%	41	569.8445	2	-11.02	63	72
(F)VRIPKEQGVL(S)	95%	41	569.8485	2	-3.98	63	72
(K)EQGVLSFWR(G)	95%	67	561.2722	2	-32.75	68	76
(K)EQGVLSFWR(G)	95%	56	561.276	2	-25.97	68	76
(K)EQGVLSFWR(G)	95%	56	561.2836	2	-12.4	68	76
(K)EQGVLSFWR(G)	95%	29	561.288	2	-4.55	68	76
(K)EQGVLSFWR(G)	95%	56	561.2881	2	-4.37	68	76
(K)EQGVLSFWR(G)	95%	34	561.2902	2	-0.62	68	76
(R)GNLANVIR(Y)	95%	39	856.4885	1	-13.33	77	84
(R)GNLANVIR(Y)	95%	41	856.4966	1	-3.86	77	84
(R)YFPTQALNFAFK(D)	95%	44	723.8729	2	-2.13	85	96
(R)YFPTQALNFAFK(D)	95%	40	723.8817	2	10	85	96
(R)YFPTQALNFAFKDVFK(Q)	95%	12	646.0041	3	-1.34	85	100
(K)QIFMSGVDKK(T)	95%	22	605.3027	2	-26.06	101	110
(F)MSGVDKKTQFW(R)	95%	28	663.8319	2	4.1	104	114
(A)TSLCFVYPLDFAR(T)	95%	51	794.8927	2	-2.97	130	142
(T)SLCFVYPLDFAR(T)	95%	58	744.3627	2	-11.45	131	142
(T)SLCFVYPLDFAR(T)	95%	38	744.3675	2	-5	131	142

(T)SLCFVYPLDFAR(T)	95%	43	744.3683	2	-3.92	131	142
(T)SLCFVYPLDFAR(T)	95%	54	744.3685	2	-3.65	131	142
(T)SLCFVYPLDFAR(T)	95%	54	744.3692	2	-2.71	131	142
(T)SLCFVYPLDFAR(T)	95%	54	744.3701	2	-1.5	131	142
(T)SLCFVYPLDFAR(T)	95%	55	744.3704	2	-1.09	131	142
(S)LCFVYPLDFAR(T)	95%	23	700.8844	2	41.7	132	142
(S)LCFVYPLDFAR(T)	95%	26	700.8845	2	41.9	132	142
(F)VYPLDF(A)	95%	21	753.3837	1	2.56	135	140
(L)AADTGK(G)	95%	21	562.2926	1	16.9	146	151
(L)AADTGKGAAEREF(S)	95%	71	661.8239	2	5.32	146	158
(R)EFSGLGNCLVK(I)	95%	37	612.2836	2	-40.05	157	167
(R)EFSGLGNCLVK(I)	95%	28	612.2897	2	-30.07	157	167
(R)EFSGLGNCLVK(I)	95%	46	612.2947	2	-21.89	157	167
(R)EFSGLGNCLVK(I)	95%	31	612.3068	2	-2.09	157	167
(R)EFSGLGNCLVK(I)	95%	28	612.312	2	6.41	157	167
(R)EFSGLGNCLVK(I)	95%	38	612.3123	2	6.9	157	167
(R)EFSGLGNCLVK(I)	95%	30	612.3124	2	7.07	157	167
(F)SGLGNCLVKIF(K)	95%	33	604.3283	2	-0.86	159	169
(F)SGLGNCLVKIF(K)	95%	28	604.3299	2	1.8	159	169
(F)SGLGNCLVKIF(K)	95%	34	604.3312	2	3.95	159	169
(K)SDGLTGLYR(G)	95%	46	491.2605	2	14	171	179
(K)SDGLTGLYR(G)	95%	44	491.2652	2	23.6	171	179
(V)SGIVSYPFDTVR(R)	95%	54	670.8389	2	-10.44	226	237
(V)SGIVSYPFDTVR(R)	95%	53	670.8402	2	-8.5	226	237
(V)SGIVSYPFDTVR(R)	95%	58	670.8416	2	-6.41	226	237
(V)SGIVSYPFDTVR(R)	95%	58	670.8442	2	-2.53	226	237
(V)SGIVSYPFDTVR(R)	95%	51	670.8458	2	-0.14	226	237
(V)SGIVSYPFDTVR(R)	95%	44	670.8467	2	1.21	226	237

(R)RmMMQSGRAK(G) [+62.0157 at C-term K]	95%	15	637.3216	2	31.2	239	248
(K)GTLDCWAK(I)	95%	16	950.4234	1	-17.52	255	262
(K)GTLDCWAK(I)	95%	15	950.4366	1	-3.61	255	262
(F)KGALSNVF(R)	95%	29	835.4623	1	-5.9	274	281

c denotes Cys alkylation by iodoacetamide during trypsin digestion
m denotes Met oxidation
