# Supplementary material for: The Suppressor of AAC2 Lethality SAL1 Modulates Sensitivity of Heterologously Expressed Artemia ADP/ATP Carrier to Bongkrekate in Yeast
Source: PLoS One. 2013 Sep 20;8(9):e74187. doi: 10.1371/journal.pone.0074187 (PMC3779231; doi:10.1371/journal.pone.0074187)
Supplement: Excel Sheet S1 — Mass spectrometric analysis of Artemia mitochondria. Homologues of identified Sal1p, SCaMC-2 (isoform 1) and SCaMC-3 are listed in six sheets, each representing the results of an independent experiment. (PDF) [file pone.0074187.s002.pdf]

Identified Proteins (38/1010)	Accession Number	Molecular Weight	Protein Grouping Ambiguity	Alternative names in Mus musculus
solute carrier family 25 (mitochondrial carrier, adenine nucleotide translocator), member 4	NP_031476	33 kDa	TRUE	
solute carrier family 25 (mitochondrial carrier, phosphate carrier), member 3	NP_598429	40 kDa		
PREDICTED: similar to ADP/ATP translocase 2 (Adenine nucleotide translocator 2) (ANT 2) (ADP,ATP carrier protein 2) (Solute carrier family 25 member 5) isoform 1	XP_485652	33 kDa	TRUE	
solute carrier family 25, member 1	NP_694790	34 kDa		
solute carrier family 25 (mitochondrial carrier oxoglutarate carrier), member 11	NP_077173	34 kDa		
solute carrier family 25 (mitochondrial carrier, Aralar), member 12	NP_766024	75 kDa		
solute carrier family 25, member 29	NP_851845	33 kDa		
solute carrier family 25, member 34	NP_001013802	34 kDa		
PREDICTED: solute carrier family 25 (mitochondrial carrier), member 18	XP_110620	34 kDa		
solute carrier family 25 (mitochondrial carrier, adenine nucleotide translocator), member 13	NP_056644	74 kDa		
solute carrier family 30 (zinc transporter), member 6	NP_659047	51 kDa		
solute carrier family 25 (mitochondrial carrier; adenine nucleotide translocator), member 31	NP_848473	35 kDa	TRUE	
mitochondrial Ca2+-dependent solute carrier	NP_666230	57 kDa		SCaMC-2 isoform 1
solute carrier family 16, member 1	NP_033222	53 kDa		
solute carrier family 25 (mitochondrial carrier, Graves disease autoantigen), member 16	NP_780403	36 kDa		
solute carrier family 25, member 37	NP_080607	38 kDa		
solute carrier family 25, member 35	NP_082324	33 kDa		
solute carrier family 25, member 39	NP_080818	39 kDa		
solute carrier family 25, member 27	NP_082987	36 kDa		
solute carrier family 25, member 46	NP_080441	46 kDa		
solute carrier family 25, member 41	NP_780542	35 kDa		
PREDICTED: similar to solute carrier family 25, member 5	XP_892944	19 kDa		
solute carrier family 22 member 4	NP_062661	62 kDa		
solute carrier family 25 (mitochondrial carrier, glutamate), member 22	NP_080922	35 kDa		
solute carrier family 25, member 36	NP_620095	34 kDa		
solute carrier family 25, member 28	NP_660138	39 kDa		
solute carrier family 25 (mitochondrial carrier, phosphate carrier), member 26	NP_080531	29 kDa		
solute carrier family 25, member 44	NP_848811	35 kDa		
solute carrier family 25 (mitochondrial carrier ornithine transporter), member 15	NP_851842	33 kDa		
PREDICTED: similar to mitochondrial solute carrier protein isoform 1	XP_110743	38 kDa		
solute carrier family 25, member 30	NP_080508	32 kDa		
solute carrier family 25 (mitochondrial deoxynucleotide carrier), member 19	NP_080347	36 kDa		
solute carrier family 25 (mitochondrial carrier, brain), member 14	NP_035528	36 kDa		
solute carrier family 25 (mitochondrial carrier, dicarboxylate transporter), member 10	NP_038798	32 kDa		
PREDICTED: similar to ADP/ATP translocase 2 (Adenine nucleotide translocator 2) (ANT 2) (ADP,ATP carrier protein 2) (Solute carrier family 25 member 5)	XP_484885	33 kDa	TRUE	
solute carrier family 25, member 42	NP_001007571	35 kDa		
solute carrier family 25, member 23	NP_080153	52 kDa		ScaMC-3, MCSC2
solute carrier family 25, member 5	NP_031477	33 kDa	TRUE	

Identified Proteins (16/548)	Accession Number	Molecular \	Protein Grouping Ambiguity	Alternative names in Mus musculus
solute carrier family 25, member 5	NP_031477	33 kDa	TRUE	
solute carrier family 25 (mitochondrial carrier, phosphate carrier), member 3	NP_598429	40 kDa		
solute carrier family 25 (mitochondrial carrier oxoglutarate carrier), member 11	NP_077173	34 kDa		
solute carrier family 25 (mitochondrial carrier, adenine nucleotide translocator), member 13	NP_056644	74 kDa	TRUE	
solute carrier family 25 (mitochondrial carrier, glutamate), member 22	NP_080922	35 kDa	TRUE	
PREDICTED: solute carrier family 25 (mitochondrial carrier), member 18	XP_110620-R	?	TRUE	
mitochondrial Ca2+-dependent solute carrier	NP_666230	57 kDa		SCaMC-2 isoform 1
PREDICTED: solute carrier family 25 (mitochondrial carrier), member 18	XP_110620	34 kDa	TRUE	
solute carrier family 25 (mitochondrial carrier, glutamate), member 22	NP_080922-R	?	TRUE	
solute carrier family 25, member 27	NP_082987-R	?		
solute carrier family 25, member 44	NP_848811	35 kDa		
solute carrier family 25, member 36	NP_620095-R	?		
solute carrier family 25, member 42	NP_001007571-R	?	TRUE	
solute carrier family 25, member 29	NP_851845	33 kDa		
solute carrier family 25, member 5	NP_031477-R (+1)	?	TRUE	
solute carrier family 25, member 1	NP_694790-R	?	TRUE	

Identified Proteins (5/195)	Accession Number	Molecular Weight	Protein Grouping Ambiguity
solute carrier family 25, member 5	NP_031477	33 kDa	
solute carrier family 25 (mitochondrial carrier oxoglutarate carrier), member 11	NP_077173	34 kDa	
solute carrier family 25 (mitochondrial carrier, phosphate carrier), member 3	NP_598429	40 kDa	
solute carrier family 25 (mitochondrial carrier, adenine nucleotide translocator), member 13	NP_056644	74 kDa	
solute carrier family 25, member 1	NP_694790	34 kDa	

Identified Proteins (5/195)	Accession Number	Molecular Weight	Protein Grouping Ambiguity
solute carrier family 25, member 5	NP_031477	33 kDa	
solute carrier family 25 (mitochondrial carrier oxoglutarate carrier), member 11	NP_077173	34 kDa	
solute carrier family 25 (mitochondrial carrier, phosphate carrier), member 3	NP_598429	40 kDa	
solute carrier family 25 (mitochondrial carrier, adenine nucleotide translocator), member 13	NP_056644	74 kDa	
solute carrier family 25, member 1	NP_694790	34 kDa	

Identified Proteins (5/124)	Accession Number	Molecular \	Protein Grouping Ambiguity
solute carrier family 25, member 5	NP_031477	33 kDa	TRUE
solute carrier family 25 (mitochondrial carrier oxoglutarate carrier), member 11	NP_077173	34 kDa	
PREDICTED: solute carrier family 25 (mitochondrial carrier), member 18	XP_110620	34 kDa	
solute carrier family 25 (mitochondrial carrier, Graves disease autoantigen), member 16	NP_780403	36 kDa	
solute carrier family 25, member 42	NP_001007571-R	?	

Identified Proteins (6/152)	Accession Number	Molecular \	Protein Grouping Ambiguity	Alternative names in Mus musculus
solute carrier family 25, member 5	NP_031477	33 kDa		
solute carrier family 25 (mitochondrial carrier, phosphate carrier), member 3	NP_598429	40 kDa		
solute carrier family 25 (mitochondrial carrier, glutamate), member 22	NP_080922	35 kDa		
mitochondrial Ca2+-dependent solute carrier	NP_666230	57 kDa		SCaMC-2 isoform 1
solute carrier family 25 (mitochondrial carrier; adenine nucleotide translocator), member 31	NP_848473-R	?	TRUE	
mitochondrial Ca2+-dependent solute carrier	NP_666230-R	?		
